# *In vitro* aging alters the gene expression and secretome composition of canine adipose-derived mesenchymal stem cells

**DOI:** 10.3389/fvets.2024.1387174

**Published:** 2024-03-28

**Authors:** Marina Prišlin, Ana Butorac, Rea Bertoša, Valentina Kunić, Ivana Ljolje, Petar Kostešić, Dunja Vlahović, Šimun Naletilić, Nenad Turk, Dragan Brnić

**Affiliations:** ^1^Virology Department, Croatian Veterinary Institute, Zagreb, Croatia; ^2^Bioanalytical Laboratory II—Proteomics, Bicro Biocentre Ltd., Zagreb, Croatia; ^3^Veterinary Clinic for Small Animals Buba, Zagreb, Croatia; ^4^Surgery, Orthopedics and Ophthalmology Clinic, Faculty of Veterinary Medicine, University of Zagreb, Zagreb, Croatia; ^5^Department for Pathological Morphology, Croatian Veterinary Institute, Zagreb, Croatia; ^6^Department of Microbiology and Infectious Diseases with Clinic, Faculty of Veterinary Medicine, University of Zagreb, Zagreb, Croatia

**Keywords:** canine adipose-derived mesenchymal stem cell, *in vitro*, gene expression, secretome, *in vitro* aging, long-term culture, veterinary regenerative medicine

## Abstract

**Introduction:**

Canine adipose-derived mesenchymal stem cells (cAD-MSCs) hold therapeutic promise due to their regenerative potential, particularly within their secretome. However, concerns arise regarding the impact of *in vitro* cultivation necessitated for storing therapeutic doses, prompting this study to comprehensively explore the impact of *in vitro* aging on gene expression and secretome composition.

**Methods:**

The study involved collecting abdominal adipose tissue samples from nine healthy female dogs, from which cAD-MSCs were extracted and cultured. Stem cells were validated through trilineage differentiation assays and flow cytometry immunophenotyping. Gene expression profiling using RT-qPCR array, and cAD-MSCs secretome LC-MS/MS analysis, were conducted at passages 3 and 6 to reveal gene expression and protein composition alterations during *in vitro* culture.

**Results and Discussion:**

The results demonstrate that the gene expression and secretome composition of cAD-MSCs were impacted by *in vitro* aging. Among many alterations in gene expression between two passages, two significant downregulations were noted in the MSC-associated PTPRC and IL10 genes. While the majority of proteins and their functional characteristics were shared between passages, the influence of cell aging on secretome composition is highlighted by 10% of proteins being distinctively expressed in each passage, along with 21 significant up- and downregulations. The functional attributes of proteins detected in passage 3 demonstrated a greater inclination towards supporting the regenerative capacity of cAD-MSCs. Moreover, proteins in passage 6 exhibited a noteworthy correlation with the blood coagulation pathway, suggesting an elevated likelihood of coagulation events. To the best of our knowledge, this study presents the first original perspective on the changes in secretome composition that occur when cAD-MSCs age *in vitro*. Furthermore, it contributes to broadening the currently restricted knowledge base concerning the secretome of cAD-MSCs. In conclusion, our findings show that the regenerative potential of cAD-MSCs, as well as their secretome, may be compromised by *in vitro* aging. Therefore, our study suggests a preference for earlier passages when considering these cells for therapeutic applications.

## 1 Background

New approaches in veterinary regenerative medicine instill hope by providing increasing evidence of advancements in treating previously incurable diseases through stem cells (1–3). The canine adipose-derived mesenchymal stem cells (cAD-MSCs) are a focal point of current research, given the abundance, ease of collection, and higher proliferation rate of AD-MSCs in contrast to alternative mesenchymal stem cell (MSCs) resources (4–6). The cAD-MSCs have been confirmed to possess regenerative capabilities facilitated by their multipotency and, notably, their ability to modulate the immune response (7–9).

Based on most recent studies, the prospect of cellular regeneration is found in the paracrine activity of cAD-MSCs, called secretome (10–12). The secretome encompasses soluble factors, including cytokines, growth factors, morphogens, chemokines, and non-soluble factors, known as extracellular vesicles containing proteins, lipids and RNAs (12). Formerly viewed as cellular debris, it is now established that these biomolecules play a pivotal role in modulating various biological processes such as cell proliferation, survival, differentiation, immunomodulation, anti-apoptosis, and angiogenesis (11). From the current point of view, cAD-MSCs secretome provides several advantages over cAD-MSCs cell therapy, such as a lower risk of thrombosis and tumor formation and easier manufacturing, handling, and storage. Furthermore, secretome could be mass-produced under controlled conditions, tailored for therapeutic effects, and stored for immediate use in acute conditions without the need for a donor or time-consuming cell expansion (10, 12, 13).

However, to achieve and store therapeutic doses of cAD-MSCs and their secretome, *in vitro* cultivation is needed. Throughout the long-term cultivation process, MSCs undergo replicative senescence, leading to a potential reduction or absence of positive therapeutic outcomes, as extensively reviewed in human research (14, 15). Long-term cultivated MSCs undergo enlargement and adopt a hypertrophic morphology, aligning with significant changes in biological features, gene expression profile, differentiation, and immunomodulatory potential (14, 16).

In contrast to human MSCs, there has been limited investigation into the impact of *in vitro* aging on gene expression profiles and secretome composition of cAD-MSCs. Our prior research has contributed to a better understanding of gene expression changes during *in vitro* passages, albeit on a limited scale (17, 18), indicating the need for further investigation. To our knowledge, no data has been published regarding alterations in the secretome composition during long-term culture of cAD-MSCs. Only two studies investigated cAD-MSCs’ secretome; the initial characterization of soluble factors and exosomes confirmed their immunomodulatory potential (5). Moreover, additional research reaffirmed the immunosuppressive effects of cAD-MSCs secretome exosomes *in vitro* (19).

Therefore, the primary objective of this study was to enhance the understanding of the effects of *in vitro* aging on the gene expression and secretome composition of cAD-MSCs.

## 2 Methods

### 2.1 Ethics approval and consent to participate

The present research was evaluated and approved by the Ethics Board of the Croatian Veterinary Institute, approval code Z-IV-4-2022/19, May 9, 2019, and Veterinary Ethics Committee at the Faculty of Veterinary Medicine, University of Zagreb, approval code 640-01/20-17/10, February 20, 2020, 640-01/20-17/55, September 28, 2020, and 640-01/22-02/07, April 20, 2022. Canine donor’s owners provided written informed consent before sampling.

### 2.2 Adipose tissue collection, cAD-MSCs extraction, and propagation

The research involved obtaining abdominal adipose tissue samples from nine clinically healthy female dogs (*Canis lupus familiaris*) who underwent elective ovariohysterectomy or ovariectomy surgical procedures. We decided to collect only female donors due to the abundance of adipose tissue at elective surgeries compared to male donors. The latter would need laparoscopic sampling to obtain the adequate quantity, which we considered to be against the 3R approach (20). Nevertheless, sex was not recognized as influential in cell surface marker expression, viability, proliferation, or differentiation potential (21). The acquisition of adipose tissue, cAD-MSCs extraction, and propagation adhered to the established protocol previously detailed in Krešić et al. (18). Briefly, 1–7 g of adipose tissue (Table 1), depending on the availability, was obtained from biomedical waste of ovarian mesostructure, placed into a sterile Falcon tube devoid of the medium, and stored at 4°C until transportation to the laboratory. Table 1 details the donors’ age, breed, and adipose tissue mass. The extraction of cAD-MSCs was carried out within a 2-h window from sample collection. This process involved rinsing and cutting the adipose tissue with a scalpel, followed by enzymatic digestion using 0.5% collagenase type 1 (Gibco, Cat. 17100017). The obtained stromal vascular fraction was subsequently placed into T25 cell culture flasks (Nunc, Thermo Fisher Scientific) and cultured in basal media comprising 79% Dulbecco’s Modified Eagle Medium with Low Glucose (DMEM Low Glucose) (Gibco, Cat. 31885049), 20% Fetal Bovine Serum (FBS) (Gibco; Cat. 1027010), and 1% Penicillin/Streptomycin (Sigma-Aldrich, Cat. P4333-100ML) at 37°*C. media* was replaced after 24 h, and when adherent cells reached 90% confluence, subculturing was performed using a solution of 0.05% Trypsin and 0.02% EDTA (Sigma-Aldrich, Cat. 59417C-500ML) until proliferation arrest was observed. All experiments conducted in this study utilized freshly isolated sterility-tested cAD-MSCs.

**Table 1:** Donor information.

Donor	Age (months)	Breed	Adipose tissue mass (g)
6/21	12	German Spaniel	7.0
9/21	12	Labrador Retriever	7.0
13/21	7	Toy Poodle	1.0
14/21	7	Toy Poodle	1.2
1/22	10	Jack Russell Terrier	1.0
2/22	6	Lagotto Romagnolo	1.2
3/22	12	Medium Poodle	1.0
6/22	60	Portuguese Water Dog	2.5
7/22	36	Mixed	4.1

### 2.3 Stem cell identification

#### 2.3.1 Differentiation capacity assay

In previous research (18), the multipotency of the extracted cAD-MSCs was evaluated through trilineage differentiation, encompassing adipogenic, osteogenic, and chondrogenic lineages. This assessment was conducted in the third passage (P3). In brief, cells were cultured in triplicate in a 24-well plate (Nunc, ThermoFisher Scientific) with a basal medium. Upon reaching confluence, the medium was replaced with StemMACS AdipoDiff media for adipogenic differentiation (Milteny, Cat. 130-091-677) or StemMACS OsteoDiff media for osteogenic differentiation (Milteny, Cat. 130-091-678). Control wells were maintained in basal media. Over 14 days, the media were changed every 48–72 h. Chondrogenic differentiation was induced in spheroid form within 15 mL conical polypropylene tubes using ChondroDiff Media (Milteny, Cat. 130-091-679), while a control tube was kept in basal media. The media were changed every 48–72 h during the 21-day differentiation period. All plates and tubes were incubated at 37°C with 5% CO_2_ and 95% humidity, with the tube lids ajar. Oil Red O (Sigma-Aldrich, Cat. 01391-250ML) staining was employed for the identification of lipid droplets, and Sigmafast BCIP/NBT substrate (Sigma-Aldrich, Cat. B5655-25TAB) was used for the detection of alkaline phosphatase activity. For chondrogenic differentiation, following dehydration and paraffin embedding of spheroids, 1% Alcian blue 8GX, pH 2,5 (Sigma-Aldrich, Cat. A3157-25G) was utilized to assess the presence of aggrecan. After staining, slides or cells were examined using an Axio Observer D1 inverted microscope, and photographs were captured with a camera (total magnification: 50x-200x, Zeiss).

#### 2.3.2 Flow cytometry immunophenotyping

To validate the immunophenotype of cAD-MSCs, undifferentiated cAD-MSCs at P3 were subjected to flow cytometric analysis. In our prior study (18), we systematically assessed the immunophenotypic traits of cAD-MSCs across passages, spanning P1–P6. Statistical analysis demonstrated no significant differences in the expression of positive and negative cluster of differentiation (CD) markers between early (P1, P2, and P3) and late passages (P4, P5, and P6). Since experimental conditions adhered to the previously established protocol (18), cAD-MSC immunophenotyping was specifically conducted only at P3 for stem cell identification. These experiments were executed on the BD FACSVerse instrument (BD Biosciences). In brief, daily performance QC was maintained using CS&T beads (BD Biosciences) with lot numbers 92,323 and 13,697 before the initiation of each experiment. For the cAD-MSCs immunophenotyping, a single-stain labeling approach was employed, with 3 μL of antibody per 10^5^ cells. The antibody panel used in the study is outlined in Table 2. Experimental conditions were calibrated using unstained cells, with identical conditions maintained across all test tubes during each experiment. Following the experiment, cell viability was assessed with a Propidium Iodide staining solution (BD Biosciences, Cat. 556,463). All data files were subjected to gating using the methodology previously described (18). FACSuite software was employed to analyze 10,000 collected events, with results presented regarding the median fluorescence intensity (MFI) and percent of positive cells (% positivity). Except for CD 105, where the MFI antibody was divided by the MFI of unstained cells due to a lack of adequate isotype control for canines. The MFI fold change was determined by dividing the MFI of the specific antibody by the median MFI of the corresponding isotype control. A fold change cutoff of 1.5 was applied for biological data interpretation. The statistical analysis and visualization of flow cytometry MFI and % positivity results were done using GraphPad Prism 10.1.2., represented as Mean ± Standard Error of the Mean (SEM) unless otherwise stated.

**Table 2:** Flow cytometry antibody panel used in this study.

Antigen	Clone	Host	Fluorophore	Reactivity	Clonality	Manufacturer/Cat.
CD90	YKIX337.217	Rat	APC	Canine	Monoclonal	Thermo Fisher Scientific/17-5900-42
Isotype IgG2b, kappa	eB149/10H5	Rat	APC	N/A	Monoclonal	Thermo Fisher Scientific/17-4031-82
CD44	MEM-263	Mouse	FITC	Canine, Human, Porcine	Monoclonal	Antibodies-online/ABIN452099
Isotype IgG1	VI-AP	Mouse	FITC	N/A	Monoclonal	Antibodies-online/ABIN1741583
CD105	SN6	Mouse	PE-Cy 7	Human, published species canine	Monoclonal	Thermo Fisher Scientific/25-1057-42
CD73	N/A	Rabbit	PE	Human, Mouse, Rat, Canine, Chicken	Polyclonal	Bioss antibodies/bs-4834R-PE
Isotype IgG	N/A	Rabbit	PE	N/A	Polyclonal	Antibodies-online/ABIN376422
CD29	MEM-101A	Mouse	FITC	Canine, Human, Porcine	Monoclonal	Antibodies-online/ABIN94056
Isotype IgG1	VI-AP	Mouse	FITC	N/A	Monoclonal	Antibodies-online/ABIN1741583
CD271	ME20.4	Mouse	APC	Canine, Human, Mouse, Non-human primate, Sheep, Pig, Rabbit, Rat	Monoclonal	Thermo Fisher Scientific/17-9400-42
Isotype IgG1, kappa	P3.6.2.8.1	Mouse	APC	N/A	Monoclonal	Thermo Fisher Scientific/17-4714-42
CD45	YKIX716.13	Rat	PE	Canine	Monoclonal	Bio-Rad/MCA1042PE
Isotype IgG2b	N/A	Rat	PE	N/A	Monoclonal	Bio-Rad/MCA600P6E
CD34	1H6	Mouse	PE	Canine	Monoclonal	BD Pharmingen/559369
Isotype IgG1 kappa	N/A	Mouse	PE	N/A	Monoclonal	BD Pharmingen/550617

N/A, Not available.

### 2.4 Total RNA extraction and gene expression profiling using RT-qPCR array analysis

Gene expression analysis using an RT-qPCR array was conducted following total RNA extraction at two time points during *in vitro* culture, P3 and P6. The experimental procedure followed the methodology outlined previously (17), incorporating certain modifications. The cAD-MSCs reached approximately 90% confluence in T75 cell culture flasks (Nunc, Thermo Fisher Scientific). Subsequently, the cells were detached using a cell scraper (Nunc, Thermo Fisher Scientific) and centrifuged at 235 × *g* for 10 min. Total RNA extraction was performed using the RNeasy Mini Kit (Qiagen, Cat. 74106) with 2 M dithiothreitol (Sigma-Aldrich, Cat. 43815-1G), following the manufacturer’s instructions for approximately 10^7^ cells. The extracted RNA was stored at −80°C until gene expression analysis.

Before commencing gene expression analysis, the RNA integrity score (RIS) and 28S:18S ratio were determined using the QIAxcel RNA QC Kit (Qiagen, Cat. 929104) on the QIAxcel Advanced capillary electrophoresis device (Qiagen) following manufacturer instructions. RIS number < 7 and 28S:18S <1 were considered low-quality RNA and excluded from further analysis.

Genomic DNA elimination and complementary DNA synthesis from 800 ng of RNA was accomplished using the RT2 First Strand kit (Qiagen, Cat. 330401) as instructed by the manufacturer. Subsequently, a commercially available RT^2^ Profiler™ PCR Array for Dog Mesenchymal Stem Cells (PAFD-082ZR, Qiagen) with SYBR Green-optimized primer assays (Qiagen, Cat. 330603) was applied, following the manufacturer’s instructions for Rotor-Gene Q (Qiagen). The array featured primers targeting 84 genes, categorized as stemness markers, MSC-specific, MSC-associated, and MSC-differentiation genes associated with osteogenesis, adipogenesis, chondrogenesis, myogenesis, and tenogenesis. Detailed information, including gene names, symbols, and NCBI sequences, was thoroughly documented in the Supplementary material 1.

Following data acquisition, normalization, and in-depth analysis were conducted using the specialized RT2 Profiler PCR Array Data Analysis software, accessible online at https://dataanalysis2.qiagen.com/pcr (accessed November 10, 2023). The software analyzes the data and performs statistical analysis, which is fully explained and provided in a detailed report (Supplementary material 1). The statistical significance was calculated based on a Student’s t-test of the replicate 2^ (− Delta CT) values for each gene in the control and treatment groups. The software automatically appointed a fold change cutoff of 2.0, equal to log_2_Fold Change ±1.0. The gene expression profiling data are publicly available on the NCBI Gene Expression Omnibus (GEO) database (Accession Number GSE255585). Data visualization was carried out using GraphPad Prism 10.1.2.

### 2.5 LC-MS/MS proteomic analysis of cAD-MSCs secretome

The alterations in the proteome composition of the cAD-MSCs secretome during *in vitro* culture were also assessed at two specific time points, P3 and P6, in randomly selected six donors (6/21, 9/21, 14/21, 1/22, 6/22, and 7/22). The cells were seeded in six replicates at 10^5^ cells per milliliter in 24-well plates (Nunc, Thermo Fisher Scientific). They were conditioned in basal media (with 20% FBS) at 37°C, 5% CO_2,_ and 95% humidity until they reached 90% confluence. Subsequently, the culture medium was aspirated, and the cells were rinsed with 100% DMEM Low Glucose before being incubated in 2 mL of 100% DMEM Low Glucose. After 48 h, the secretome was carefully collected, centrifuged at 2,100 × *g* for 10 min, filtered through a sterile 0.45 μm filter (Merck), and preserved at −80°C until proteomic analysis.

For sample preparation, the secretome proteins were first reduced with dithiothreitol (Sigma Aldrich, Cat. D0632-5G) at a final concentration of 10 mM at 57°C for 30 min and subsequently alkylated with iodoacetamide (Sigma, Cat. I6125-5G) at a final concentration of 25 mM for 30 min at room temperature. The extraction of secretome proteins from the culture medium was carried out using trichloroacetic acid (Merck, Cat. 1008070100) precipitation method as previously described (22). To summarize, 1,260 μL of the medium sample was combined with sodium deoxycholate (Sigma-Aldrich, Cat. D6750-100G) at a final concentration of 0.1%. Trichloroacetic acid was added to reach a final concentration of 7.5%, causing protein precipitation on ice for 2 h. Afterward, the samples were centrifuged for 10 min at 4°C and 10,000 × *g*. The supernatant was discarded, and the pellet underwent two washes with ice-cold tetrahydrofuran (Merck, Cat. 1081011000). Finally, the pellet was reconstituted in 50 μL of 50 mM triethylammonium bicarbonate buffer (Thermo Fisher Scientific, Cat. 90,114 1 M). Protein concentration was determined using the Bradford assay, and the concentrations of all samples were adjusted with 50 mM triethylammonium bicarbonate buffer.

The proteins were subjected to enzymatic digestion with trypsin (Promega, Cat. V5117) at a final 0.01 mg/mL concentration, carried out over 18 h at 37°C and 20 × g. Peptide separation was executed using the nanoLC EASY-nLC™ 1200 System (Thermo Fisher Scientific) on a 75 μm × 250 mm RPC column. The gradient length spanned 2 h with 0–80% acetonitrile (VWR Chemicals, Cat. 83639.320), and 0.1% formic acid (Merck, Cat. 1002641000) with a flow rate of 1 μL/min, and the injection volume was 2 μL. The nanoLC system was connected to the Q Exactive™ Plus Hybrid Quadrupole-Orbitrap™ Mass Spectrometer (Thermo Fisher Scientific). The mass spectrometer operated in a data-dependent mode, where MS1 spectra were recorded within the range of 350–1,800 m/z at a resolution of 70,000. The top 12 ions were selected for fragmentation (MS2) and recorded at a resolution of 17,500.

Raw data were analyzed using Scaffold Quant Q + S 5.3.0 utilizing protein sequence data of *Canis lupus familiaris* reference proteome obtained from the UniProt database Proteome ID UP000805418 (accessed on October 30, 2023, with a total of 20,991 entries). Search parameters included the allowance of a missed trypsin cleavage, carbamidomethylation as a fixed modification, and a precursor and fragment ion mass tolerance of 10 ppm. Peptide search results were subsequently analyzed with Scaffold Quant version 5.0.3, employing untargeted label-free quantification and statistical analysis based on the spectral counting method, utilizing a *t*-test to verify the results of secretome composition. The proteins were filtered to include only the ones with a minimum of two identified peptide sequences. Statistical probability was defined at the *p* < 0.05 unless otherwise stated. A fold change cutoff of 1.3 was appointed, equal to log_2_Fold Change ±0.3785. Gene Ontology (GO) Panther 18.0 was utilized in the research of cellular components, protein class, molecular function, biological process, and pathways analysis, using Fisher’s exact test type and false discovery rate correction; data presented as raw *p* value and false discovery rate (FDR). The mass spectrometry proteomics data have been deposited to the ProteomeXchange Consortium via the PRIDE (23) partner repository with the dataset identifier PXD049324 and 10.6019/PXD049324. Data visualization was carried out using GraphPad Prism 10.1.2.

## 3 Results

### 3.1 Prolonged *in vitro* cultivation results in morphological changes

The cAD-MSCs cultures from all donors were successfully established. The spindle-shaped isolated cells underwent *in vitro* cultivation, where morphological changes were observed with each passage, exhibiting an increase in both size and granularity, coupled with a decrease in cell population density attributable to senescence, i.e., *in vitro* aging. Proliferation arrest, on average, occurred at P8. Microscopic images of three representative donors in P3 and P6 were presented in Supplementary material 2.

### 3.2 cAD-MSCs multipotency and stem cell immunophenotype are preserved *in vitro*

**Figure 1:**
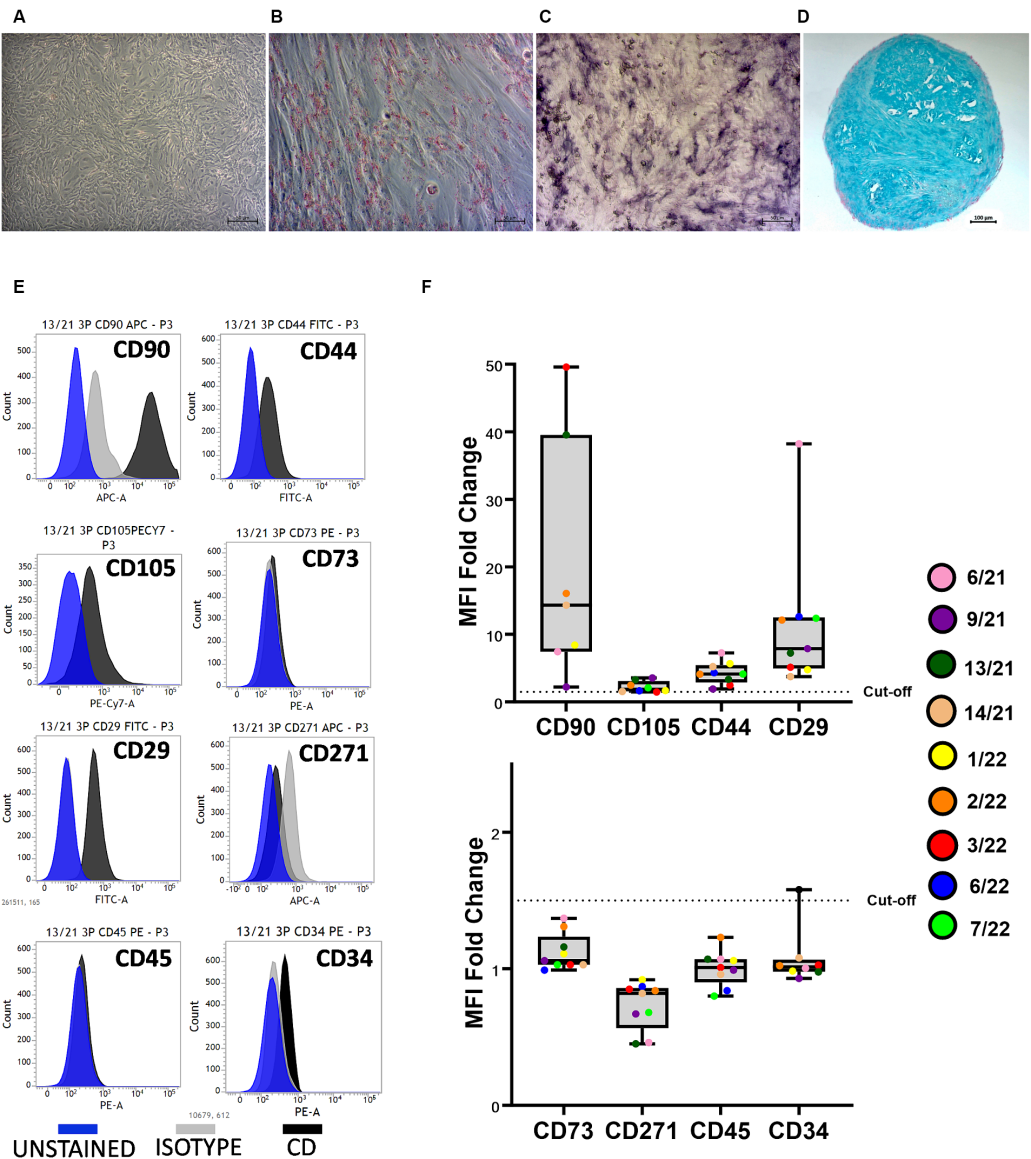
Canine adipose-derived mesenchymal stem cells (cAD-MSCs) retain multipotency and stem cell immunophenotype in passage 3 *in vitro*. Undifferentiated cAD-MSCs morphology **(A)**. Adipogenic differentiation: lipid droplets are red **(B)**. Osteogenic differentiation: The activity of alkaline phosphatase is purple **(C)**. Chondrogenic differentiation: stained with Alcian Blue (aggrecan is blue) **(D)**. Microscopic pictures were taken with an Axiovert camera on the Axio Observer D1 microscope (Zeiss). Scale bars: 50 μm **(A–C)**, 100 μm **(D)**. Representative flow cytometry histograms for each CD marker in comparison to isotype and unstained cells. Blue represents unstained, gray represents isotype, and black represents CD surface markers **(E)**. Whisker-box plot representation of Median Fluorescence Intensity (MFI) fold changes in cAD-MSCs for surface markers CD90, CD105, CD44, CD29 (box plot above), CD73, CD271, CD45, and CD34 (box plot below) in the third passage. The dotted line indicates the cutoff value of 1.5, and colorful dots with the legend on the right represent individual MFI fold change values for each donor **(F)**.

The trilineage differentiation capacity (adipogenic, osteogenic, and chondrogenic) of all isolated cells was preserved at P3, and the cAD-MSCs were confirmed to possess differentiation potential following the International Society for Cellular Therapy (ISCT) criteria (24). Microscopic images in Figure 1 depict undifferentiated cells (A), adipogenic (B), osteogenic (C), and chondrogenic (D) differentiation from a representative 6/21 donor experiment.

Isolated cAD-MSCs at P3 underwent flow cytometry analysis, revealing the presence of markers CD90 (87.64 ± 5.3%), CD105 (41.44 ± 11.64%), CD44 (74.32 ± 6.33%), and CD29 (97.93 ± 0.65%), confirming the cells’ stem cell status according to ISCT guidelines. Notably, the expression of CD73, for which a polyclonal antibody was utilized, was not observed. The analysis also showed the absence of expression for markers CD271 (1.61 ± 1.21%), CD45 (0%), and CD34 (4.01 ± 1.65%). Figure 1E represents CD surface marker expression histograms for donor 13/21 in comparison to isotype and unstained cells. Additionally, Whisker-box plots depict the MFI fold changes for all CD markers, with donor-specific data points represented by different-colored dots (Figure 1F).

### 3.3 Gene expression profiling of *in vitro* aged cAD-MSCs showed significant downregulation in MSC-associated genes

**Figure 2:**
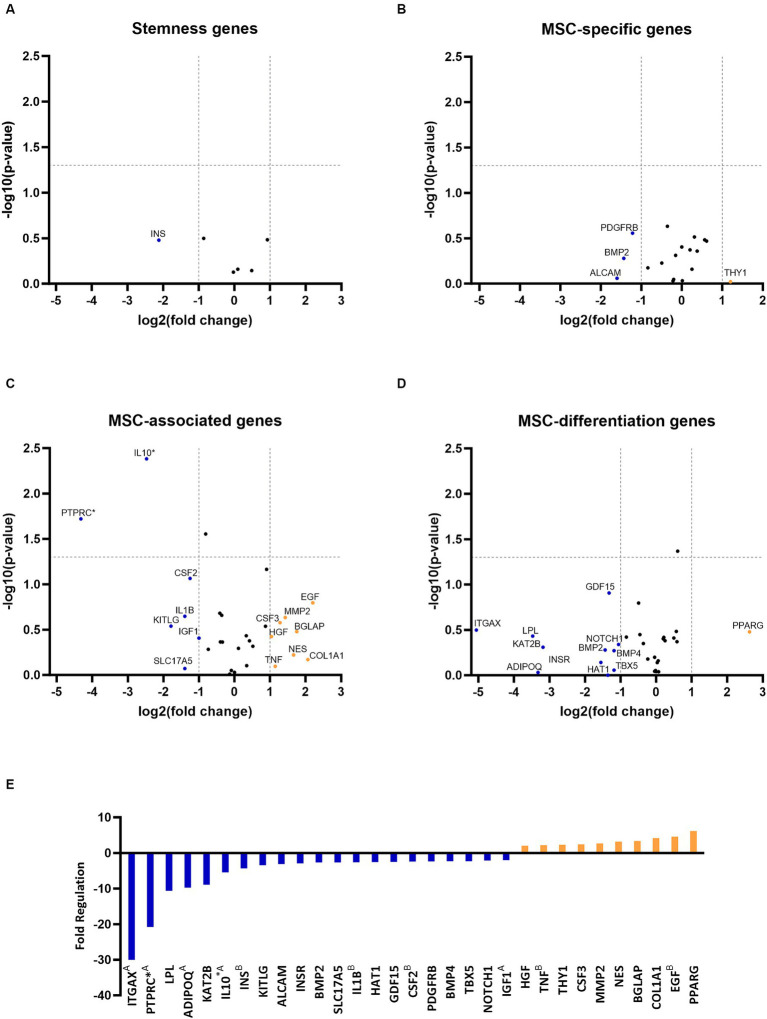
Gene expression profiling of canine adipose-derived mesenchymal stem cells showed significant downregulation due to *in vitro* aging. The volcano plots illustrate the expression profile of stemness **(A)** mesenchymal stem cell (MSC) specific **(B)** MSC-associated **(C)** and MSC-differentiation genes **(D)** following cultivation in the sixth passage (P6) in comparison to P3. Downregulated and upregulated genes were marked in blue and orange, respectively. ^*^p < 0.05 **(A–D)**. Fold regulation of up- and downregulated genes. Downregulated genes are marked in blue, while upregulated genes are marked in orange. ^A^Gene’s expression is relatively low (Ct > 30) in one sample and reasonably detected (Ct <30) in the other sample, suggesting that the actual fold change value is at least as large as the calculated and reported fold change result. ^B^This gene’s average threshold cycle is relatively high (> 30), meaning that its relative expression level is low in both control and test samples, and the *p* value for the fold change is either unavailable or relatively high (*p* > 0.05). ^*^p < 0.05 **(E)**.

RNA quality analysis confirmed that the RNA in every sample was of high quality (Table 3). Gene expression analysis revealed that the overall change in expression between P3 and P6 was significantly downregulated for MSC-associated genes IL10 and PTPRC. Nonsignificant downregulations and upregulations were observed in stemness, MCS-specific, MSC-associated, and MSC-differentiation genes, as indicated in Figures 2A–D. Figure 2E represents fold-regulation values for downregulated and upregulated genes. Fold-regulation represents fold-change results in a biologically meaningful way. The complete RT2 Profiler PCR Array Data Analysis software report is attached as a Supplementary material 1.

**Table 3:** Results of RNA QC analysis; RNA integrity score (RIS), and 28S:18S ratio for individual donors.

Donor	P3		P6	
	*RIS*	*28S:18S*	*RIS*	*28S:18S*
6–21	10	2,33	9,7	1,8
9–21	9,7	1,7	9,3	1,62
13–21	10	2,56	9,9	2,03
14–21	10	1,91	9,7	1,58
1–22	10	2,43	10	2,62
2–22	9,9	2,45	9,9	2,16
3–22	9,3	2,27	9,1	2,28
6–22	9	2,2	10	2,39
7–22	10	1,87	10	2,02

### 3.4 Proteomic analysis of cAD-MSCs secretome showed significant up- and downregulations

During the proteomic analysis of cAD-MSCs secretome, 1,187 proteins were detected in P3 and P6, with 90% proteins in common. A complete list of detected proteins, their gene symbols, *t*-test significance, and fold change results, is provided in the Supplementary material 3.

**Figure 3:**
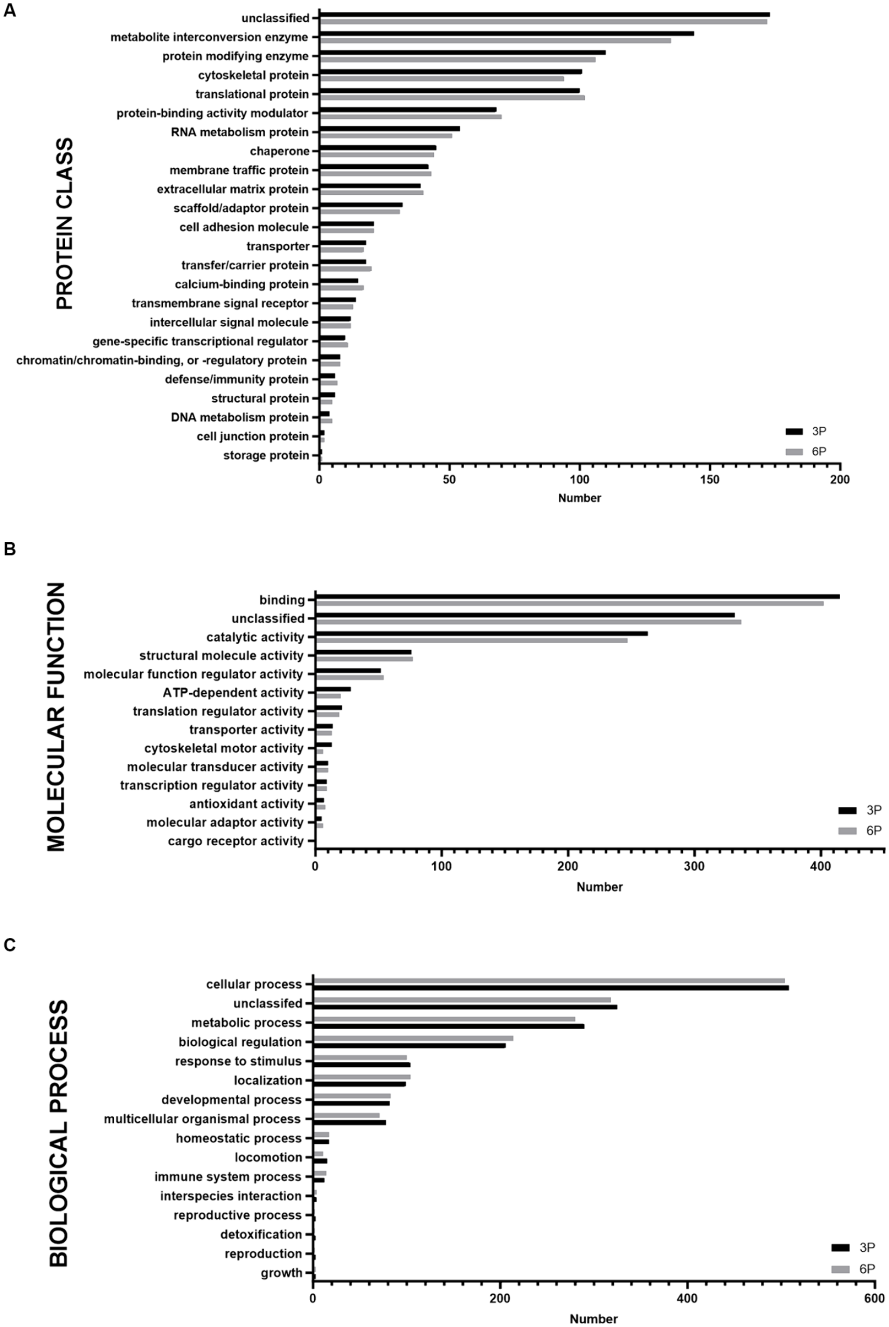
Graphical representation of functional Gene Ontology (GO) complete classification of proteins found in canine adipose-derived mesenchymal stem cells secretome comparing third passage (P3) and sixth passage (P6) by number of proteins detected. Functional GO complete classification by protein classes **(A)**. Functional GO complete classification by molecular functions **(B)**. Functional GO complete classification by biological processes **(C)**.

Proteins detected in P3 and P6 were compared by functional annotation to cellular components, protein class, molecular function, and biological process on GO complete analysis (Figure 3). Similar involvement in all detected functions was observed. Cellular component analysis revealed that ≈75% of protein belonged to cellular anatomical entities, 22.7% belonged to protein-containing complexes, and the rest were unassigned. More than 50% of cAD-MSCs secretome proteins comprised metabolite interconversion enzyme translational proteins, protein modifying enzymes, cytoskeletal proteins, translational proteins, and protein-binding activity modulators (Figure 3A). Their molecular functions were mainly binding and catalytic activity (Figure 3B), and they were predominantly involved in cellular and metabolic processes, biological regulation, response to stimulus, and localization (Figure 3C).

**Figure 4:**
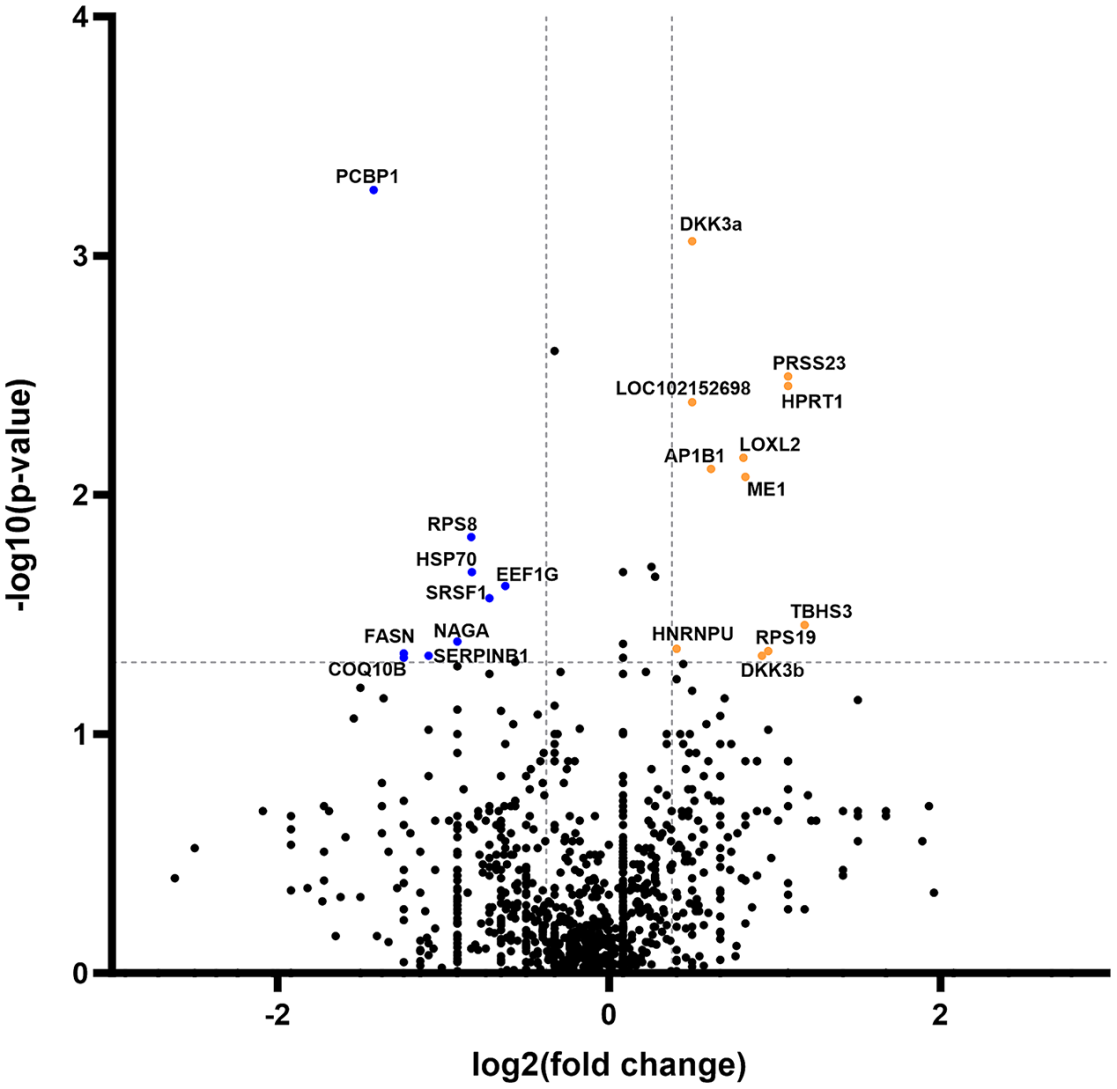
Volcano plot of commonly expressed canine adipose-derived mesenchymal stem cells secretome proteins comparing passage 6 to passage 3. In the graphical representation, blue dots signify proteins that have undergone statistically significant downregulation (*p* < 0.05) with a fold change exceeding 1.3. Conversely, orange dots denote proteins that exhibit statistical significance (*p* < 0.05) in upregulation, also with a fold change greater than 1.3. Both categories of dots are associated with protein corresponding specific gene symbol name.

Secretome analysis of commonly produced proteins showed downregulation in 648 proteins, while 302 proteins were upregulated (Figure 4). Correlation of statistically significant (*p* < 0.05) and fold change cutoff >1.3 proteins indicated nine biologically significant downregulated proteins—PCBP1, RPS8, HSP70, EEF1G, SRSF1, NAGA, FASN, SERPINB1, and COQ10B and 12 biologically significant upregulated proteins—DKK3a (isomer 1), PRSS23, HPRT1, LOC102152698, LOXL2, AP1B1, ME1, EIF3F, THBS3, HNRNPU, RPS19, and DKK3b (isomer 2) (Figure 4).

The 10% of explored proteins were distinctive for each passage; 63 and 52 proteins were expressed only in P3 and P6, respectively. Distinctive proteins expressed in P3 were grouped with biologically significant downregulated proteins (Group P3), while those expressed in P6 were grouped with biologically significant upregulated proteins (Group P6). Moreover, these two groups were analyzed through GO Panther Pathways analysis to see if any critical protein pathways were influenced by the *in vitro* aging of cAD-MSCs. Group P3 proteins showed significant involvement in cytoskeletal regulation by Rho GTPase (*p* = 7.45E^−12^, FDR = 1.2E^−9^), nicotinic acetylcholine receptor signaling pathway (*p* = 1.2E^−7^, FDR = 9.8E^−6^), inflammation mediated by chemokine and cytokine signaling pathway (*p* = 6.7E^−7^, FDR = 3.6E^−5^), Wnt signaling pathway (*p* = 7.3E^−5^, FDR = 0.00235), and CCKR signaling map (*p* = 0.000601, FDR = 0.0161). In group P6, the expressed proteins were significantly related to the xanthine and guanine salvage pathway (*p* = 0.000193, FDR = 0.031), adenine and hypoxanthine salvage pathway (*p* = 0.000377, FDR = 0.0202), and blood coagulation pathway (*p* = 0.00032, FDR = 0.0258).

## 4 Discussion

*In vitro* culture is necessary to produce cAD-MSCs or their secretome for therapeutic applications. However, long-term culture leads to *in vitro* cell aging, thereby potentially compromising the regenerative and immunomodulatory capabilities of cAD-MSCs. Thus, this research aimed to explore the influence of *in vitro* aging on the gene expression and secretome composition of cAD-MSCs. Our findings demonstrate alterations of cAD-MSC-expressed genes and the secretome proteomic profile of cAD-MSCs induced by *in vitro* aging.

Initially, the isolated cells conformed to the criteria stipulated by the ISCT for identifying human stem cells (24) (Figure 1). Given the absence of standardized criteria for verifying canine stem cells, this investigation builds upon our prior research (17, 18) to establish the reproducibility and standardization of cAD-MSCs’ immunophenotype and three-lineage multipotency. In addition to previously explored cAD-MSCs’ surface markers CD90, CD44, CD29, CD105, CD73, CD45, and CD271 (18), in this study, we investigated surface marker CD34, which exhibited a % positivity of less than 5%, aligning with similar observations reported elsewhere (21, 25).

*In vitro* aging influenced the gene expression of cAD-MSCs, as confirmed by the present study, which detected 21 downregulated and 10 upregulated genes (Figure 2). In comparison to our earlier study (17), similar findings were observed regarding the cAD-MSCs-expressed genes, but expansion of the donor pool and advancement to later passages demonstrated significant downregulation of MSC-associated *PTPRC* and *IL10* genes (Figure 2). *PTPRC* is a gene coding for CD45 surface marker protein expressed by all hematopoietic cells including hematopoietic stem cells (26). While it is considered a negative marker for human MSCs (24), the exact criteria for cAD-MSCs are yet to be established. Immunophenotyping revealed the absence of CD45 protein on the surface of cAD-MSCs in all donors during the P3 stage. However, it revealed 4% of extracted cells were positive for hematopoietic stem cell marker CD34, and these cells can express the gene for *PTPRC*/CD45 (27). On the other hand, *IL10* is a major immune regulatory cytokine with profound anti-inflammatory functions produced by various immune and non-immune cells (28). Co-expression of *IL10* in MSCs was proven beneficial, contributing to anti-inflammatory activity in humans and canines (29–34). Our findings suggest that the observed expression of *IL10* in earlier passages, such as P3, may confer beneficial effects regarding inflammation. However, we must interpret these results with caution since the expression of *PTPRC* and *IL10* genes in the early passages may originate from various cell types within the adipose-derived stromal vascular fraction (35). Therefore, the downregulation of these two genes in later passages, such as P6, may result from the absence of these cells.

The secretome composition of cAD-MSCs was also impacted by *in vitro* aging. To our knowledge, this study represents the first investigation into the impact of prolonged passages of cAD-MSCs on secretome composition. Furthermore, our study significantly contributes to expanding the limited database on the secretome of cAD-MSCs. Most detected proteins and their functional characteristics were shared between early and later passages (P3 and P6) (Figure 3). Similar observations were documented in the secretome and exosomes of feline AD-MSCs (36) and canine bone marrow MSCs when compared to cAD-MSCs (5).

However, a notable functional difference was observed for the group of distinctive proteins (10% of detected proteins) in each passage (Group P3 and Group P6). Firstly, P3 exhibited substantial involvement in cytoskeletal regulation by Rho GTPase which plays pivotal roles in diverse cellular processes, encompassing gene expression, cytoskeletal dynamics, survival, cell division, cell adhesion, polarity, and vesicle trafficking (37). As cells age, these events decrease, consistent with the cellular quiescence observed in a later passage (P6) of the present study. Group P3 proteins displayed a significant association with the nicotinic acetylcholine receptor, Wnt, and CCKR signaling pathways, which play crucial roles in MSC regenerative function (38), cell proliferation/apoptosis inhibition (39), and adipocyte differentiation (40), respectively. The last, but very important finding within Group P3, concerns proteins associated with inflammation mediated by the chemokine and cytokine signaling, regulating trafficking and migration of immune cells (41). Moreover, Group P6 proteins were significantly associated with the blood coagulation pathway. Given MSCs’ apparent pro-thrombotic role, it is imperative to emphasize the necessity for stringent control of culture passages before administration to prevent adverse coagulation events (42). Collectively, the results of the present study, coupled with corroborating evidence regarding human MSCs (14, 15), underscore the need for caution when extending cAD-MSCs to later passages.

In addition to groups of distinctive proteins detected in both passages, our investigation unveiled modifications in a subset of shared proteins across both passages, comprising precisely 21 entities that exhibited significant up- or downregulation (Figure 4). Notably, the downregulated proteins in P6 (HSP70, SRSF1, SERPINB1, and COQ10B) are intricately associated with stem cells’ regenerative pathways in the treatment of various diseases (43–47). Nevertheless, these findings warrant further research and confirmatory testing, which are presently constrained by the insufficient accessibility of canine-specific reagents (e.g., monoclonal antibodies).

In conclusion, our findings underscore significant alterations in the regenerative capacity of cAD-MSCs and their secretome due to *in vitro* aging. Thus, prioritizing earlier passages of these cells may be advisable to optimize their regenerative potential for therapeutic applications.

## Data availability statement

The original contributions presented in the study are publicly available. This data can be found at: https://www.ebi.ac.uk/pride/; PXD049324. The gene expression profiling data are publicly available on the NCBI Gene Expression Omnibus (GEO) database (Accession Number GSE255585) https://www.ncbi.nlm.nih.gov/geo/query/acc.cgi?acc=GSE255585.

## Ethics statement

The animal studies were approved by Ethics Board of the Croatian Veterinary Institute and Veterinary Ethics Committee at the Faculty of Veterinary Medicine, University of Zagreb. The studies were conducted in accordance with the local legislation and institutional requirements. Written informed consent was obtained from the owners for the participation of their animals in this study.

## Author contributions

MP: Writing – review & editing, Writing – original draft, Visualization, Validation, Software, Methodology, Investigation, Formal Analysis, Data curation, Conceptualization. AB: Writing – review & editing, Writing – original draft, Software, Methodology, Investigation, Data curation. RB: Writing – review & editing, Software, Methodology, Investigation, Data curation. VK: Writing – review & editing, Visualization, Methodology, Investigation. IL: Writing – review & editing, Resources. PK: Writing – review & editing, Resources. DV: Writing – review & editing, Methodology. ŠN: Writing – review & editing, Methodology, Investigation. NT: Writing – review & editing, Supervision. DB: Writing – review & editing, Writing – original draft, Supervision, Project administration, Funding acquisition, Conceptualization.
