# Supplementary material for: In vitro aging alters the gene expression and secretome composition of canine adipose-derived mesenchymal stem cells
Source: Front Vet Sci. 2024 Mar 28;11:1387174. doi: 10.3389/fvets.2024.1387174 (PMC11006985; doi:10.3389/fvets.2024.1387174)
Supplement: Supplementary file 1 [file Data_Sheet_1.PDF]

RT^2 Profiler PCR Array Gene Expression Analysis Report

Marina Prišlin

11-10-2023

Table of Contents

Introduction 3
Summary and workflow 4
Gene Table 5
Data analysis setup 8
Data quality control (QC) 9
Normalization analysis 10
Result 11
Fold regulation and p-value 11
Scatter Plot 12
Volcano Plot 15
Clustergram 18
Heat Map 19
What's next 20
Gene Expression 21

Introduction

Cataloged arrays

RT² Profiler PCR Arrays are highly reliable and sensitive gene expression profiling tools for analyzing focused panels of genes insignal transduction, biological processes or disease research pathways using real-time PCR. Each cataloged RT² Profiler PCR Array contains a list of the pathway-focused genes as well as five housekeeping (reference) genes on the array. In addition, eacharray contains a panel of proprietary controls to monitor genomic DNA contamination (GDC) as well as the first strand synthesis (RTC) and real-time PCR efficiency (PPC). The qPCR Assays used in PCR Arrays are laboratory-verified and optimized to workunder standard conditions enabling a large number of genes to be assayed simultaneously. Their specificity is guaranteed when RT² SYBR Green qPCR Master Mixes are used as part of the complete PCR Array System protocol.

In this study, 96 genes were profiled on 18 samples with the PAFD-082Z.

Summary and workflow

Cataloged arrays

1. Mature RNA was isolated using an RNA extraction kit according to the manufacturer’s instructions.
2. RNA quality was determined using a spectrophotometer and was reverse transcribed using a cDNA conversion kit.
3. The cDNA was used on the real-time RT² Profiler PCR Array (QIAGEN, Cat. no. PAFD-082Z) in combination with RT² SYBR® Green qPCR Mastermix (Cat. no. 330529).

C_T values were exported to an Excel file to create a table of C_T values. This table was then uploaded on to the data analysis web portal at http://www.qiagen.com/geneglobe. Samples were assigned to controls and test groups. C_T values were normalized based on a/an Manual Selection of reference genes.

The data analysis web portal calculates fold change/regulation using delta delta C_T method, in which delta C_T is calculated between gene of interest (GOI) and an average of reference genes (HKG), followed by delta-delta C_T calculations (delta C_T (Test Group)-delta C_T (Control Group)). Fold Change is then calculated using 2 ^ (-delta delta C_T) formula. The data analysis web portal also plots scatter plot, volcano plot, clustergram, and heat map.

This data analysis report was exported from the QIAGEN web portal at GeneGlobe.

Gene Table

Position	RefSeq Number	Symbol	Description
A01	NM_001003215	ABCB1	ATP-binding cassette, sub-family B (MDR/TAP), member 1
A02	XM_534781	ACTA2	Actin, alpha 2, smooth muscle, aorta
A03	NM_001006644	ADIPOQ	Adiponectin, C1Q and collagen domain containing
A04	NM_001313804	ALCAM	Activated leukocyte cell adhesion molecule
A05	NM_001146034	ANPEP	Alanyl (membrane) aminopeptidase
A06	XM_533303	ANXA5	Annexin A5
A07	NM_001002975	BDNF	Brain-derived neurotrophic factor
A08	XM_014115322	BGLAP	Bone gamma-carboxyglutamate (gla) protein
A09	XM_534351	BMP2	Bone morphogenetic protein 2
A10	NM_001287170	BMP4	Bone morphogenetic protein 4
A11	NM_001197052	BMP7	Bone morphogenetic protein 7
A12	NM_001003042	CASP3	Caspase 3, apoptosis-related cysteine peptidase
B01	NM_001197022	CD44	CD44 molecule (Indian blood group)
B02	XM_843188	CEBPD	CCAAT/enhancer binding protein (C/EBP), delta
B03	NM_001003090	COL1A1	Collagen, type I, alpha 1
B04	NM_001003245	CSF2	Colony stimulating factor 2 (granulocyte-macrophage)
B05	XM_005624543	CSF3	Colony stimulating factor 3 (granulocyte)
B06	NM_001137652	CTNNB1	Catenin (cadherin-associated protein), beta 1, 88kDa
B07	NM_001003094	EGF	Epidermal growth factor
B08	XM_005625330	ENG	Endoglin
B09	NM_001003217	ERBB2	V-erb-b2 erythroblastic leukemia viral oncogene homolog 2, neuro/glioblastoma derived oncogene homolog (avian)
B10	XM_005624145	FASN	Fatty acid synthase
B11	XM_005619337	FGF10	Fibroblast growth factor 10
B12	XM_003432481	FGF2	Fibroblast growth factor 2 (basic)
C01	XM_005616335	FUT1	Fucosyltransferase 1 (galactoside 2-alpha-L-fucosyltransferase, H blood group)
C02	XM_546927	FZD9	Frizzled family receptor 9
C03	XM_541938	GDF15	Growth differentiation factor 15
C04	XM_542974	GDF5	Growth differentiation factor 5
C05	XM_540103	GDF7	Growth differentiation factor 7
C06	XM_014107406	GTF3A	General transcription factor IIIA
C07	NM_001002964	HGF	Hepatocyte growth factor (hepapoietin A; scatter factor)
C08	XM_543429	HNF1A	HNF1 homeobox A
C09	NM_001003291	ICAM1	Intercellular adhesion molecule 1
C10	NM_001003174	IFNG	Interferon gamma

Position	RefSeq Number	Symbol	Description
C11	NM_001313855	IGF1	Insulin-like growth factor 1 (somatomedin C)
C12	NM_001003077	IL10	Interleukin 10
D01	NM_001037971	IL1B	Interleukin 1, beta
D02	NM_001003301	IL6	Interleukin 6 (interferon, beta 2)
D03	NM_001130093	INS	Insulin
D04	XM_005633222	INSR	Insulin receptor
D05	XM_014110784	ITGAV	Integrin, alpha V (vitronectin receptor, alpha polypeptide, antigen CD51)
D06	XM_547049	ITGAX	Integrin, alpha X (complement component 3 receptor 4 subunit)
D07	XM_005616948	ITGB1	Integrin, beta 1 (fibronectin receptor, beta polypeptide, antigen CD29 includes MDF2, MSK12)
D08	XM_005634783	JAG1	Jagged 1
D09	XM_014106497	KAT2B	K(lysine) acetyltransferase 2B
D10	NM_001048024	KDR	Kinase insert domain receptor (a type III receptor tyrosine kinase)
D11	NM_001012735	KITLG	KIT ligand
D12	NM_001197073	LIF	Leukemia inhibitory factor (cholinergic differentiation factor)
E01	XM_005640309	HAT1	Similar to histone aminotransferase 1 (predicted)
E02	XM_544435	HDAC1	Similar to histone deacetylase 1
E03	XM_005635734	LPL	Lipoprotein lipase
E04	XM_014113367	MCAM	Melanoma cell adhesion molecule
E05	NM_001003337	MITF	Microphthalmia-associated transcription factor
E06	XM_014109407	MMP2	Matrix metallopeptidase 2 (gelatinase A, 72kDa gelatinase, 72kDa type IV collagenase)
E07	XM_014115306	NES	Nestin
E08	XM_548191	NGFR	Nerve growth factor receptor
E09	XM_005625433	NOTCH1	Notch 1
E10	XM_532221	NT5E	5'-nucleotidase, ecto (CD73)
E11	XM_540961	NUDT6	Nudix (nucleoside diphosphate linked moiety X)-type motif 6
E12	NM_001003382	PDGFRB	Platelet-derived growth factor receptor, beta polypeptide
F01	XM_548290	PIGS	Phosphatidylinositol glycan anchor biosynthesis, class S
F02	XM_538830	POU5F1	POU class 5 homeobox 1
F03	NM_001024632	PPARG	Peroxisome proliferator-activated receptor gamma
F04	XM_005618555	PROM1	Prominin 1
F05	XM_005627993	PTK2	Protein tyrosine kinase 2
F06	XM_005622278	PTPRC	Protein tyrosine phosphatase, receptor type, C
F07	NM_001003273	RHOA	Ras homolog gene family, member A
F08	XM_005642335	RUNX2	Runt-related transcription factor 2
F09	XM_005627533	SLC17A5	Solute carrier family 17 (anion/sugar transporter), member 5
F10	XM_005615393	SMAD4	SMAD family member 4
F11	XM_005621177	SMURF1	SMAD specific E3 ubiquitin protein ligase 1

Position	RefSeq Number	Symbol	Description
F12	XM_537589	SMURF2	SMAD specific E3 ubiquitin protein ligase 2
G01	XM_005639752	SOX2	SRY (sex determining region Y)-box 2
G02	NM_001002978	SOX9	SRY (sex determining region Y)-box 9
G03	XM_548568	TBX5	T-box 5
G04	NM_001003309	TGFB1	Transforming growth factor, beta 1
G05	XM_849026	TGFB3	Transforming growth factor, beta 3
G06	NM_001287129	THY1	Thy-1 cell surface antigen
G07	NM_001003244	TNF	Tumor necrosis factor
G08	NM_001003298	VCAM1	Vascular cell adhesion molecule 1
G09	NM_001110502	VEGFA	Vascular endothelial growth factor A
G10	NM_001287023	VIM	Vimentin
G11	NM_001002932	VWF	Von Willebrand factor
G12	XM_539327	WNT3A	Wingless-type MMTV integration site family, member 3A
H01	NM_001195845	ACTB	Actin, beta
H02	XM_535458	B2M	Beta-2-microglobulin
H03	NM_001003357	HPRT1	Hypoxanthine phosphoribosyltransferase 1
H04	XM_844899	PGK1	Phosphoglycerate kinase 1
H05	NM_001313766	RPL13A	Ribosomal protein L13a
H06	SA_00130	FGDC	Dog Genomic DNA Contamination
H07	SA_00104	RTC	Reverse Transcription Control
H08	SA_00104	RTC	Reverse Transcription Control
H09	SA_00104	RTC	Reverse Transcription Control
H10	SA_00103	PPC	Positive PCR Control
H11	SA_00103	PPC	Positive PCR Control
H12	SA_00103	PPC	Positive PCR Control

Data analysis setup

Sample management

Sample ID	Sample Name	Group
1	6-21 3P	Control Group
2	9-21 3P	Control Group
3	13-21 3p	Control Group
4	14-21 3p	Control Group
5	1-22 3p	Control Group
6	2-22 3p	Control Group
7	3-22 3p	Control Group
8	6-22 3p	Control Group
9	7-22 3p	Control Group
10	6-21 6p	Group 1
11	9-21 6p	Group 1
12	13-21 6p	Group 1
13	14-21 6p	Group 1
14	1-22 6p	Group 1
15	2-22 6p	Group 1
16	3-22 6p	Group 1
17	6-22 6p	Group 1
18	7-22 6p	Group 1

Pre-amplification

A pre-amplification using the appropriate species- and pathway-specific RT^2 PreAMP Primer Mix was not performed and no corrections were made to C_T values during the data analysis procedure other than the use of the C_T cut-off value.

Lower limit of detection

The C_T cut-off was set to 35

Data quality control (QC)

Quality checks performed and results

Test Performed	Test Result
1. PCR Array Reproducibility	All Samples Passed
2. Reverse Transcription Efficiency	All Samples Passed
3. Genomic DNA Contamination	All Samples Passed

Normalization analysis

Manual Selection

Groups	Samples	ACTB	B2M	HPRT1	PGK1	RPL13A	Geometric Mean	Average Geometric Mean
Control Group	6-21 3P	13.66	16.25	13.40	16.45	14.34	14.76	14.84
Control Group	9-21 3P	12.74	16.91	13.41	15.90	14.35	14.58	
Control Group	13-21 3p	13.41	16.63	13.03	15.57	13.89	14.44	
Control Group	14-21 3p	13.62	16.16	13.35	15.94	14.21	14.61	
Control Group	1-22 3p	14.90	18.82	13.95	16.89	14.49	15.71	
Control Group	2-22 3p	15.36	17.86	13.32	15.46	14.57	15.24	
Control Group	3-22 3p	16.30	16.28	13.83	16.91	13.97	15.40	
Control Group	6-22 3p	14.24	16.34	13.67	16.06	13.73	14.76	
Control Group	7-22 3p	12.75	15.68	13.01	15.65	13.74	14.11	
Group 1	3-22 6p	14.28	15.86	13.14	16.04	13.80	14.58	14.65
Group 1	6-22 6p	13.28	17.37	12.85	15.18	14.28	14.51	
Group 1	6-21 6p	14.12	16.35	13.83	16.62	14.51	15.04	
Group 1	9-21 6p	13.54	17.19	13.36	15.45	14.28	14.70	
Group 1	13-21 6p	13.40	16.36	12.72	15.37	13.90	14.29	
Group 1	14-21 6p	14.15	15.31	13.76	16.42	14.22	14.74	
Group 1	1-22 6p	14.07	17.03	13.80	16.52	14.46	15.12	
Group 1	2-22 6p	13.70	17.52	13.35	15.39	14.25	14.77	
Group 1	7-22 6p	12.70	16.43	12.90	15.27	13.70	14.13	

In the Manual Selection method, the arithmetic or geometric means of the data from the assays for the housekeeping / reference genes listed in the table were used to normalize the raw data.

Result

Fold regulation and p-value

Test Group	Control Group	Fold Regulation Threshold	p-Value Threshold
Group 1	Control Group	2	0.05

Position	Gene Symbol	Fold Regulation	p-Value	Comments
C12	IL10	-5.46	0.004148	A
F06	PTPRC	-20.83	0.019001	A

Fold-Change (2^(-Delta Delta C_T)) is the normalized gene expression (2^(- Delta C_T)) in the Test Sample divided the normalized gene expression (2^(- Delta C_T)) in the Control Sample. Fold-Regulation represents fold-change results in a biologically meaningful way. Fold-change values greater than one indicates a positive- or an up-regulation, and the fold-regulation is equal to the fold-change. Fold-change values less than one indicate a negative or down-regulation, and the fold-regulation is the negative inverse of the fold-change.

The p values are calculated based on a Student's t-test of the replicate 2^(- Delta C_T) values for each gene in the control group and treatment groups, and p values less than 0.05 are indicated in red. The p-value calculation used is based on parametric, unpaired, two-sample equal variance, two-tailed distribution – a method widely accepted in scientific literature.

Scatter Plot

Test Group	Control Group	Fold Regulation Threshold
Group 1	Control Group	2

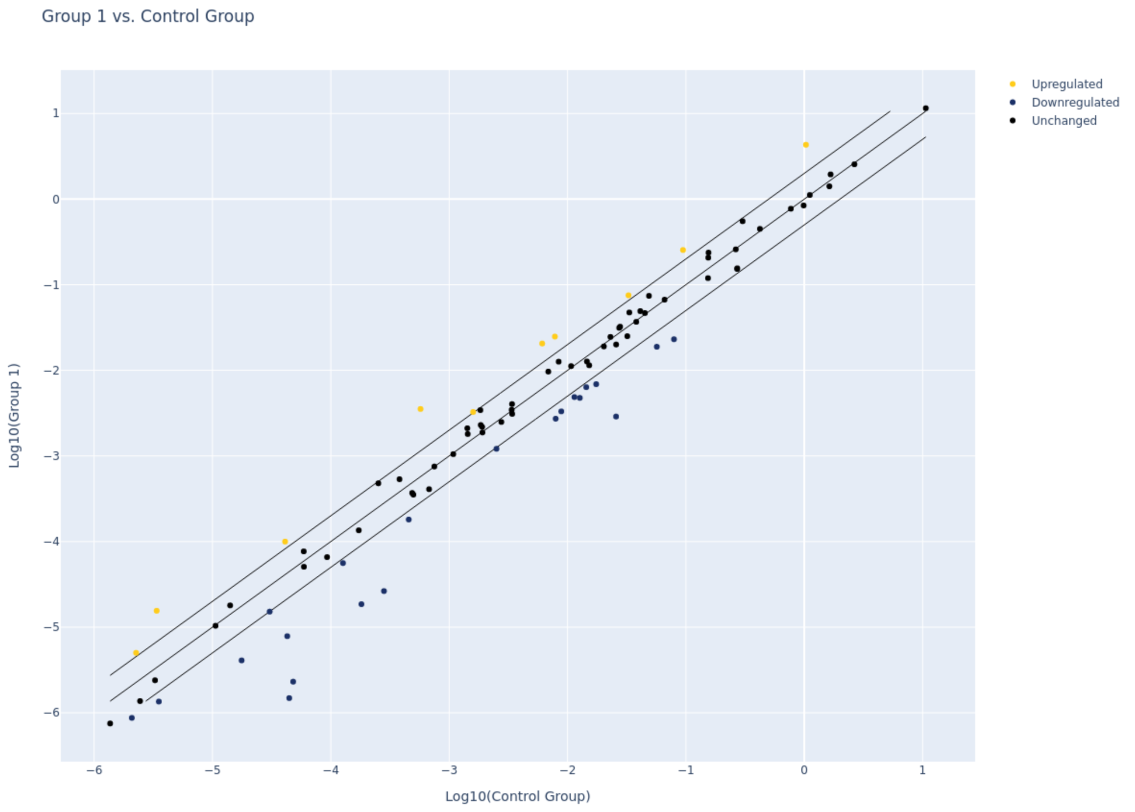

The Scatter Plot compares the normalized expression of every gene on the PCR Array between the two selected groups by plotting them against one another to quickly visualize large gene expression changes. The center diagonal line indicates unchanged gene expression, while the outer diagonal lines indicate the selected fold regulation threshold. Genes with data points beyond the outer lines in the upper left and lower right corners are up-regulated or down-regulated, respectively, by more than the fold regulation threshold in the y-axis Group relative to the x-axis Group.

Genes Over-Expressed in Group 1 vs. Control Group

Position	Gene Symbol	Fold Regulation	Comments	RT² qPCR Assay Catalog #
A08	BGLAP	3.37		PPF00334A
B03	COL1A1	4.18		PPF00230A
B05	CSF3	2.43		PPF11809A
B07	EGF	4.60	B	PPF00236A
C07	HGF	2.05		PPF00046A
E06	MMP2	2.69		PPF00308A
E07	NES	3.17		PPF10300A
F03	PPARG	6.18		PPF00186A
G06	THY1	2.30		PPF09626A
G07	TNF	2.21	B	PPF00476A

Genes Under-Expressed in Group 1 vs. Control Group

Position	Gene Symbol	Fold Regulation	Comments	RT² qPCR Assay Catalog #
A03	ADIPOQ	-9.77	A	PPF00204A
A04	ALCAM	-3.01		PPF03427A
A09	BMP2	-2.67		PPF02535A
A10	BMP4	-2.26		PPF10452A
B04	CSF2	-2.39	B	PPF00477A
C03	GDF15	-2.52		PPF07107A
C11	IGF1	-2.00	A	PPF12664A
C12	IL10	-5.46	A	PPF00207A
D01	IL1B	-2.61	B	PPF00525A
D03	INS	-4.31	B	PPF06441A
D04	INSR	-2.92		PPF07200A
D06	ITGAX	-30.03	A	PPF10013A
D09	KAT2B	-8.91		PPF02464A
D11	KITLG	-3.44		PPF00105A
E01	HAT1	-2.53		PPF14470A
E03	LPL	-10.66		PPF00205A
E09	NOTCH1	-2.07		PPF04820A
E12	PDGFRB	-2.35		PPF00638A
F06	PTPRC	-20.83	A	PPF10210A
F09	SLC17A5	-2.66		PPF01096A
G03	TBX5	-2.26		PPF10871A

Volcano Plot

Test Group	Control Group	Fold Regulation Threshold	p-Value Threshold
Group 1	Control Group	2	0.05

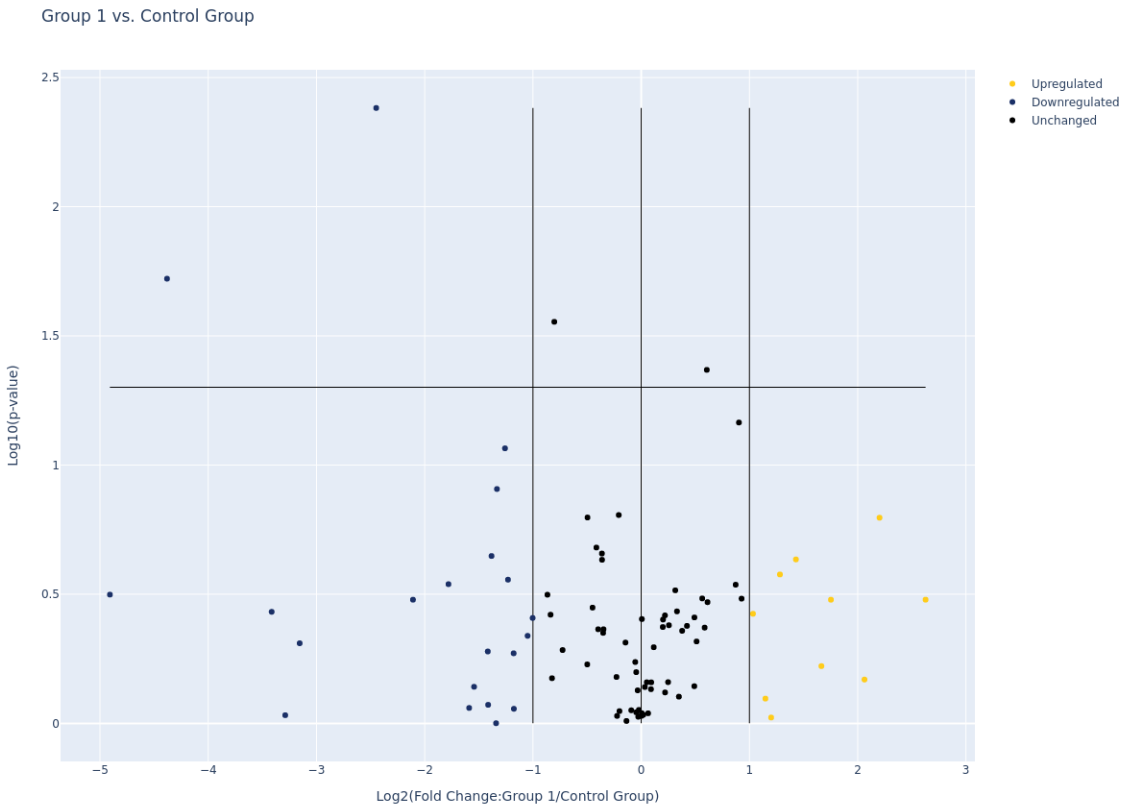

The Volcano Plot identifies significant gene expression changes by plotting the log2 of the fold changes in gene expression on the x-axis versus their statistical significance on the y-axis. The center vertical line indicates unchanged gene expression, while the two outer vertical lines indicate the selected fold regulation threshold. The horizontal line indicates the selected p-value threshold. Genes with data points in the far upper left (down-regulated) and far upper right (up-regulated) sections meet the selected fold regulation and p-value thresholds. By combining the fold change results with the p-value statistical test results, genes with both large and small expression changes that are statistically significant are easily visualized.

Genes Over-Expressed in Group 1 vs. Control Group

Position	Gene Symbol	Fold Regulation	p-Value	Comments	RT² qPCR Assay Catalog #
A08	BGLAP	3.37	0.332198		PPF00334A
B03	COL1A1	4.18	0.676730		PPF00230A
B05	CSF3	2.43	0.265206		PPF11809A
B07	EGF	4.60	0.159942	B	PPF00236A
C07	HGF	2.05	0.376520		PPF00046A
E06	MMP2	2.69	0.231985		PPF00308A
E07	NES	3.17	0.600357		PPF10300A
F03	PPARG	6.18	0.332115		PPF00186A
G06	THY1	2.30	0.949461		PPF09626A
G07	TNF	2.21	0.802296	B	PPF00476A

Genes Under-Expressed in Group 1 vs. Control Group

Position	Gene Symbol	Fold Regulation	p-Value	Comments	RT² qPCR Assay Catalog #
A03	ADIPOQ	-9.77	0.930111	A	PPF00204A
A04	ALCAM	-3.01	0.871567		PPF03427A
A09	BMP2	-2.67	0.526848		PPF02535A
A10	BMP4	-2.26	0.535596		PPF10452A
B04	CSF2	-2.39	0.086178	B	PPF00477A
C03	GDF15	-2.52	0.123846		PPF07107A
C11	IGF1	-2.00	0.391019	A	PPF12664A
C12	IL10	-5.46	0.004148	A	PPF00207A
D01	IL1B	-2.61	0.224983	B	PPF00525A
D03	INS	-4.31	0.332193	B	PPF06441A
D04	INSR	-2.92	0.721887		PPF07200A
D06	ITGAX	-30.03	0.317394	A	PPF10013A
D09	KAT2B	-8.91	0.489808		PPF02464A
D11	KITLG	-3.44	0.289194		PPF00105A
E01	HAT1	-2.53	0.998855		PPF14470A
E03	LPL	-10.66	0.369992		PPF00205A
E09	NOTCH1	-2.07	0.458332		PPF04820A
E12	PDGFRB	-2.35	0.277824		PPF00638A
F06	PTPRC	-20.83	0.019001	A	PPF10210A
F09	SLC17A5	-2.66	0.847677		PPF01096A
G03	TBX5	-2.26	0.878418		PPF10871A

Clustergram

Sample	Dimension	Join Type	Color Coded
Array	2-D	Average	Genes

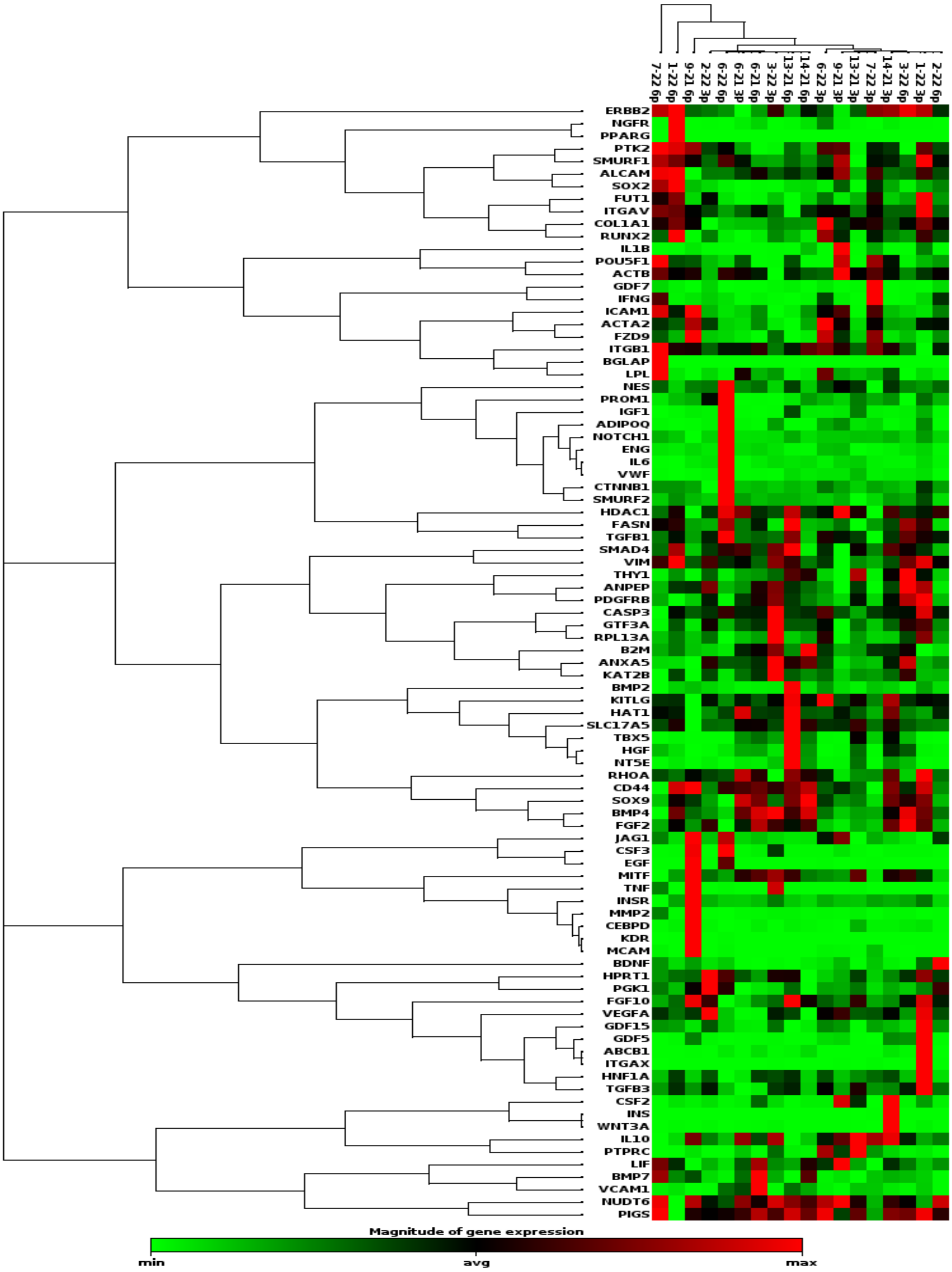

Heat Map

Test Group	Control Group
Group 1	Control Group

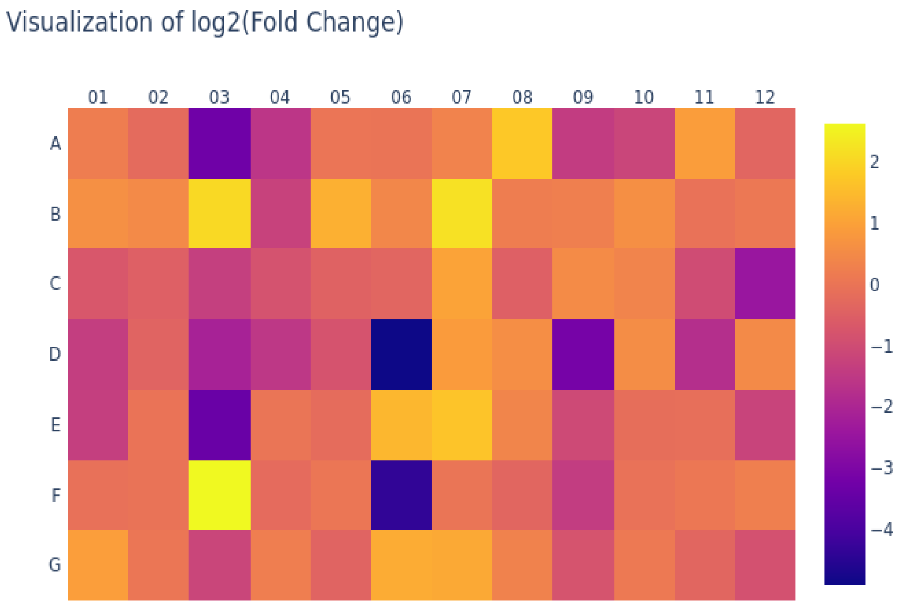

Layout	01	02	03	04	05	06	07	08	09	10	11	12
A	ABCB1 / 1.15	ACTA2 / -1.17	ADIPOQ / -9.77 / A	ALCAM / -3.01	ANPEP / 1.01	ANXA5 / 1.0	BDNF / 1.26	BGLAP / 3.37	BMP2 / -2.67	BMP4 / -2.26	BMP7 / 1.87	CASP3 / -1.28
B	CD44 / 1.53	CEBPD / 1.41	COL1A1 / 4.18	CSF2 / -2.39 / B	CSF3 / 2.43	CTNNB1 / 1.34	EGF / 4.6 / B	ENG / 1.15	ERBB2 / 1.19	FASN / 1.52	FGF10 / -1.03	FGF2 / 1.07
C	FUT1 / -1.65	FZD9 / -1.41	GDF15 / -2.52	GDF5 / -1.79 / B	GDF7 / -1.37 / B	GTF3A / -1.29	HGF / 2.05	HNF1A / -1.41	ICAM1 / 1.43	IFNG / 1.27 / B	IGF1 / -2.0 / A	IL10 / -5.46 / A
D	IL1B / -2.61 / B	IL6 / -1.32	INS / -4.31 / B	INSR / -2.92	ITGAV / -1.77	ITGAX / -30.03 / A	ITGB1 / 1.83	JAG1 / 1.5	KAT2B / -8.91	KDR / 1.48	KITLG / -3.44	LIF / 1.4
E	HAT1 / -2.53	HDAC1 / -1.02	LPL / -10.66	MCAM / 1.0	MITF / -1.15	MMP2 / 2.69	NES / 3.17	NGFR / 1.3	NOTCH1 / -2.07	NT5E / -1.11	NUDT6 / -1.1	PDGFRB / -2.35
F	PIGS / -1.07	POU5F1 / -1.02 / B	PPARG / 6.18	PROM1 / -1.17	PTK2 / 1.04	PTPRC / -20.83 / A	RHOA / 1.0	RUNX2 / -1.28	SLC17A5 / -2.66	SMAD4 / -1.03	SMURF1 / 1.05	SMURF2 / 1.19
G	SOX2 / 1.9	SOX9 / 1.02	TBX5 / -2.26	TGFB1 / 1.16	TGFB3 / -1.33	THY1 / 2.3	TNF / 2.21 / B	VCAM1 / 1.24	VEGFA / -1.74	VIM / 1.08	VWF / -1.27	WNT3A / -1.82 / B

What's next

Thank you for using the RT² Profiler Data Analysis Software.

The Data Analysis software delivers a list of expression changes in the samples from the supplied data. However, this result often only starts an investigation into the underlying mechanisms at work. In order to assist in further analysis, the QIAGEN now utilizes the latest bioinformatics tools to analyze the data and suggest regulatory mechanisms and future experiments. Please review the results from the selected tools below.

Gene Expression: This tool will help define a panel of genes based of this experiment’s results. This panel may represent aputative biomarker set, a target gene set or simply a collection of genes. The tool is designed to deliver a list of gene expressionassays that would allow the user to follow-up the results of the analyzed experiment.

Gene Expression

Test Group	Control Group	Fold Regulation Threshold	p-Value Threshold
Group 1	Control Group	2	0.05

Position	Symbol	Fold Regulation	p-Value	RT2 qPCR Assay
C12	IL10	-5.46	0.004148	PPF00207A
F06	PTPRC	-20.83	0.019001	PPF10210A
