# Supplementary material for: In vitro aging alters the gene expression and secretome composition of canine adipose-derived mesenchymal stem cells
Source: Front Vet Sci. 2024 Mar 28;11:1387174. doi: 10.3389/fvets.2024.1387174 (PMC11006985; doi:10.3389/fvets.2024.1387174)
Supplement: Supplementary file 2 [file Data_Sheet_2.PDF]

**Supplementary Material 3.** Complete list of LC-MS/MS detected proteins, their accession number, gene symbols, t-test and fold change results.

| # | Protein Name | Accession Number | Gene Name | Molecular Weight | t-test p-value | -log10 (p-value) | fold change (1.3) | log2fold change (+-0.3785) |
|---|---|---|---|---|---|---|---|---|
| **1** | Poly(rC) binding protein 1 OS=Canis lupus familiaris OX=9615 GN=PCBP1 PE=4 SV=1 | A0A8I3PTN2 | *PCBP1* | 37 kDa | 0.00053 | 3.2757241 | 0.373712312 | -1.42 |
| **2** | Dickkopf WNT signaling pathway inhibitor 3 OS=Canis lupus familiaris OX=9615 GN=DKK3 PE=3 SV=1 | A0A8I3P0C0 | *DKK3* | 38 kDa | 0.00087 | 3.0604807 | 1.414213562 | 0.5 |
| **3** | Eukaryotic translation initiation factor 3 subunit H OS=Canis lupus familiaris OX=9615 GN=EIF3H PE=4 SV=1 | A0A8I3NT41 | *EIF3H* | 39 kDa | 0.0025 | 2.60206 | 0.795536484 | -0.33 |
| **4** | Serine protease 23 OS=Canis lupus familiaris OX=9615 GN=PRSS23 PE=4 SV=1 | A0A8I3N353 | *PRSS23* | 42 kDa | 0.0032 | 2.49485 | 2.114036081 | 1.08 |
| **5** | Hypoxanthine phosphoribosyltransferase OS=Canis lupus familiaris OX=9615 GN=HPRT1 PE=3 SV=1 | A0A8I3PP34 | *HPRT1* | 26 kDa | 0.0035 | 2.455932 | 2.114036081 | 1.08 |
| **6** | Uncharacterized protein OS=Canis lupus familiaris OX=9615 GN=LOC102152698 PE=3 SV=1 | A0A8I3NNX3 | *LOC102152698* | 30 kDa | 0.0041 | 2.3872161 | 1.414213562 | 0.5 |
| **7** | Lysyl oxidase homolog OS=Canis lupus familiaris OX=9615 GN=LOXL2 PE=3 SV=1 | A0A8I3P9Y5 | *LOXL2* | 87 kDa | 0.007 | 2.154902 | 1.753211443 | 0.81 |
| **8** | AP complex subunit beta OS=Canis lupus familiaris OX=9615 GN=AP1B1 PE=3 SV=1 | A0A8I3QS06 | *AP1B1* | 102 kDa | 0.0078 | 2.1079054 | 1.531557997 | 0.615 |
| **9** | Malic enzyme OS=Canis lupus familiaris OX=9615 GN=ME1 PE=3 SV=1 | A0A8I3NJ10 | *ME1* | 61 kDa | 0.0084 | 2.0757207 | 1.767855062 | 0.822 |
| **10** | 40S ribosomal protein S8 OS=Canis lupus familiaris OX=9615 GN=RPS8 PE=3 SV=1 | A0A8I3PTU6 | *RPS8* | 25 kDa | 0.015 | 1.8239087 | 0.561360711 | -0.833 |
| **11** | Fibulin-1 OS=Canis lupus familiaris OX=9615 GN=FBLN1 PE=3 SV=1 | A0A8I3NH97 | *FBLN1* | 78 kDa | 0.02 | 1.69897 | 1.193335743 | 0.255 |
| **12** | Heat shock protein 70 OS=Canis lupus familiaris OX=9615 GN=HSP70 PE=3 SV=1 | A0A8I3NQW2 | *HSP70* | 67 kDa | 0.021 | 1.6777807 | 0.563309614 | -0.828 |
| **13** | Eukaryotic translation initiation factor 3 subunit F OS=Canis lupus familiaris OX=9615 GN=EIF3F PE=3 SV=1 | A0A8I3NFD2 | *EIF3F* | 39 kDa | 0.021 | 1.6777807 | 1.0604672 | 0.0847 |
| **14** | RAB7A, member RAS onco family OS=Canis lupus familiaris OX=9615 GN=RAB7A PE=4 SV=1 | A0A8I3P9F1 | *RAB7A* | 24 kDa | 0.022 | 1.6575773 | 1.21167266 | 0.277 |
| **15** | Elongation factor 1-gamma OS=Canis lupus familiaris OX=9615 GN=TUT1 PE=4 SV=1 | A0A8I3NDU5 | *EEF1G* | 50 kDa | 0.024 | 1.6197888 | 0.647970483 | -0.626 |
| **16** | Serine and arginine rich splicing factor 1 OS=Canis lupus familiaris OX=9615 GN=SRSF1 PE=4 SV=1 | A0A8I3NZQ3 | *SRSF1* | 26 kDa | 0.027 | 1.5686362 | 0.60583633 | -0.723 |
| **17** | Thrombospondin 3 OS=Canis lupus familiaris OX=9615 GN=THBS3 PE=3 SV=1 | A0A8I3NN32 | *THBS3* | 91 kDa | 0.035 | 1.455932 | 2.265767771 | 1.18 |
| **18** | Alpha-galactosidase OS=Canis lupus familiaris OX=9615 GN=NAGA PE=3 SV=1 | A0A8I3NKJ0 | *NAGA* | 44 kDa | 0.041 | 1.3872161 | 0.530343871 | -0.915 |
| **19** | Kininogen 1 OS=Canis lupus familiaris OX=9615 GN=KNG1 PE=4 SV=1 | A0A8I3PBR7 | *KNG1* | 48 kDa | 0.042 | 1.3767507 | 1.0604672 | 0.0847 |
| **20** | Heterogeneous nuclear ribonucleoprotein U OS=Canis lupus familiaris OX=9615 GN=HNRNPU PE=4 SV=1 | A0A8I3MPM7 | *HNRNPU* | 89 kDa | 0.044 | 1.3565473 | 1.32592576 | 0.407 |
| **21** | Small ribosomal subunit protein eS19 OS=Canis lupus familiaris OX=9615 GN=RPS19 PE=3 SV=1 | A0A8I3MJY3 | *RPS19* | 18 kDa | 0.045 | 1.3467875 | 1.943961976 | 0.959 |
| **22** | Fatty acid synthase OS=Canis lupus familiaris OX=9615 GN=FASN PE=4 SV=1 | A0A8I3MVX3 | *FASN* | 295 kDa | 0.046 | 1.3372422 | 0.423372656 | -1.24 |

| | | | | | | | | |
|---|---|---|---|---|---|---|---|---|
| **23** | Serpin family B member 1 OS=Canis lupus familiaris OX=9615 GN=SERPINB1 PE=3 SV=1 | A0A8I3P1P3 | *SERPINB1* | 49 kDa | 0.047 | 1.3279021 | 0.469761375 | -1.09 |
| **24** | Dickkopf WNT signaling pathway inhibitor 3 OS=Canis lupus familiaris OX=9615 GN=DKK3 PE=3 SV=1 | A0A8I3S2K3 | *DKK3* | 37 kDa | 0.047 | 1.3279021 | 1.893427262 | 0.921 |
| **25** | 10 kDa heat shock protein, mitochondrial OS=Canis lupus familiaris OX=9615 GN=COQ10B PE=3 SV=1 | A0A8I3PNX9 | *COQ10B* | 11 kDa | 0.048 | 1.3187588 | 0.423372656 | -1.24 |
| **26** | Reticulocalbin 1 OS=Canis lupus familiaris OX=9615 GN=RCN1 PE=4 SV=1 | A0A8I3PY67 | *RCN1* | 20 kDa | 0.048 | 1.3187588 | 1.0604672 | 0.0847 |
| **27** | Heterogeneous nuclear ribonucleoproteins A2/B1 OS=Canis lupus familiaris OX=9615 GN=HNRNPA2B1 PE=4 SV=1 | A0A8I3NCE9 | *HNRNPA2B1* | 37 kDa | 0.05 | 1.30103 | 0.675018993 | -0.567 |
| **28** | Fructose-bisphosphate aldolase OS=Canis lupus familiaris OX=9615 GN=ALDOC PE=3 SV=1 | A0A8I3NII5 | *ALDOC* | 39 kDa | 0.051 | 1.2924298 | 1.363202607 | 0.447 |
| **29** | Uncharacterized protein OS=Canis lupus familiaris OX=9615 PE=4 SV=1 | A0A8I3NLR5 | | 17 kDa | 0.052 | 1.2839967 | 0.530343871 | -0.915 |
| **30** | Lamin A/C OS=Canis lupus familiaris OX=9615 GN=LMNA PE=3 SV=1 | A0A8I3NEK0 | *LMNA* | 74 kDa | 0.055 | 1.2596373 | 0.816203046 | -0.293 |
| **31** | Activated RNA polymerase II transcriptional coactivator p15 OS=Canis lupus familiaris OX=9615 GN=LOC610407 PE=3 SV=1 | A0A8I3NM63 | *LOC610407* | 14 kDa | 0.055 | 1.2596373 | 1.16634937 | 0.222 |
| **32** | Interleukin enhancer binding factor 3 OS=Canis lupus familiaris OX=9615 GN=ILF3 PE=4 SV=1 | A0A8I3S4C9 | *ILF3* | 96 kDa | 0.056 | 1.251812 | 0.60583633 | -0.723 |
| **33** | GTP-binding nuclear protein Ran OS=Canis lupus familiaris OX=9615 GN=LOC119867725 PE=3 SV=1 | A0A8I3P1U7 | *LOC119867725* | 24 kDa | 0.056 | 1.251812 | 1.0604672 | 0.0847 |
| **34** | Glyceraldehyde-3-phosphate dehydrogenase OS=Canis lupus familiaris OX=9615 GN=LOC477441 PE=3 SV=1 | A0A8I3Q407 | *LOC477441* | 36 kDa | 0.059 | 1.229148 | 1.32592576 | 0.407 |
| **35** | Chondroitin sulfate proteoglycan 4 OS=Canis lupus familiaris OX=9615 GN=CSPG4 PE=4 SV=1 | A0A8I3PEA6 | *CSPG4* | 246 kDa | 0.064 | 1.19382 | 0.353553391 | -1.5 |
| **36** | Cytochrome c OS=Canis lupus familiaris OX=9615 GN=CYCS PE=1 SV=2 | P00011 | *CYCS* | 12 kDa | 0.066 | 1.1804561 | 1.414213562 | 0.5 |
| **37** | Ubiquitin carboxyl-terminal hydrolase OS=Canis lupus familiaris OX=9615 GN=USP14 PE=3 SV=1 | A0A8I3MEG2 | *USP14* | 55 kDa | 0.071 | 1.1487417 | 0.38958229 | -1.36 |
| **38** | Serpin family E member 1 OS=Canis lupus familiaris OX=9615 GN=SERPINE1 PE=3 SV=1 | A0A8I3MDS4 | *SERPINE1* | 45 kDa | 0.071 | 1.1487417 | 1.620006947 | 0.696 |
| **39** | Complement factor I OS=Canis lupus familiaris OX=9615 GN=CFI PE=4 SV=1 | A0A8I3Q318 | *CFI* | 67 kDa | 0.072 | 1.1426675 | 2.828427125 | 1.5 |
| **40** | peptidylprolyl isomerase OS=Canis lupus familiaris OX=9615 GN=LOC119881669 PE=4 SV=1 | A0A8I3Q1V5 | *LOC119881669* | 12 kDa | 0.076 | 1.1191864 | 0.795536484 | -0.33 |
| **41** | Chaperonin containing TCP1 subunit 6A OS=Canis lupus familiaris OX=9615 PE=3 SV=1 | A0A8I3N9I8 | | 53 kDa | 0.079 | 1.1023729 | 0.530343871 | -0.915 |
| **42** | Chloride intracellular channel protein OS=Canis lupus familiaris OX=9615 PE=3 SV=1 | A0A8I3NY67 | | 23 kDa | 0.08 | 1.09691 | 0.636397468 | -0.652 |
| **43** | Importin 5 OS=Canis lupus familiaris OX=9615 GN=IPO5 PE=4 SV=1 | A0A8I3PAW8 | *IPO5* | 118 kDa | 0.083 | 1.0809219 | 0.742261785 | -0.43 |
| **44** | Uncharacterized protein OS=Canis lupus familiaris OX=9615 GN=SRSF7 PE=4 SV=1 | A0A8I3S2F3 | *SRSF7* | 26 kDa | 0.084 | 1.0757207 | 1.591072968 | 0.67 |
| **45** | Annexin OS=Canis lupus familiaris OX=9615 GN=ANXA6 PE=3 SV=1 | A0A8I3MQ36 | *ANXA6* | 52 kDa | 0.086 | 1.0655015 | 0.343885455 | -1.54 |
| **46** | Uncharacterized protein OS=Canis lupus familiaris OX=9615 GN=SET PE=3 SV=1 | A0A8I3Q0N4 | *SET* | 53 kDa | 0.091 | 1.0409586 | 0.669891801 | -0.578 |

| | | | | | | | | |
|---|---|---|---|---|---|---|---|---|
| **47** | Actin related protein 3B OS=Canis lupus familiaris OX=9615 GN=ACTR3B PE=3 SV=1 | A0A8I3NER0 | *ACTR3B* | 46 kDa | 0.091 | 1.0409586 | 1.502119927 | 0.587 |
| **48** | Small nuclear ribonucleoprotein-associated protein OS=Canis lupus familiaris OX=9615 GN=SNRPN PE=3 SV=1 | A0A8I3NZ34 | *SNRPN* | 25 kDa | 0.095 | 1.0222764 | 0.883927531 | -0.178 |
| **49** | Septin OS=Canis lupus familiaris OX=9615 GN=SEPTIN6 PE=3 SV=1 | A0A8I3S249 | *SEPTIN6* | 50 kDa | 0.096 | 1.0177288 | 0.469761375 | -1.09 |
| **50** | Kinectin 1 OS=Canis lupus familiaris OX=9615 GN=KTN1 PE=4 SV=1 | A0A8I3P790 | *KTN1* | 157 kDa | 0.096 | 1.0177288 | 1.943961976 | 0.959 |
| **51** | Large ribosomal subunit protein eL33 OS=Canis lupus familiaris OX=9615 GN=RPL35A PE=3 SV=1 | A0A8I3QGT5 | *RPL35A* | 14 kDa | 0.098 | 1.0087739 | 1.0604672 | 0.0847 |
| **52** | MYG1 exonuclease OS=Canis lupus familiaris OX=9615 GN=MYG1 PE=3 SV=1 | A0A8I3PIY9 | *MYG1* | 42 kDa | 0.1 | 1 | 0.530343871 | -0.915 |
| **53** | Sialic acid acetylesterase OS=Canis lupus familiaris OX=9615 GN=SIAE PE=4 SV=1 | A0A8I3NBZ9 | *SIAE* | 53 kDa | 0.1 | 1 | 0.795536484 | -0.33 |
| **54** | Eukaryotic translation elongation factor 2 OS=Canis lupus familiaris OX=9615 GN=EEF2 PE=4 SV=1 | A0A8I3RV50 | *EEF2* | 95 kDa | 0.1 | 1 | 0.806082831 | -0.311 |
| **55** | Apolipoprotein D OS=Canis lupus familiaris OX=9615 GN=APOD PE=3 SV=1 | A0A8I3Q4W1 | *APOD* | 23 kDa | 0.1 | 1 | 1.0604672 | 0.0847 |
| **56** | DEAD-box helicase 17 OS=Canis lupus familiaris OX=9615 GN=DDX17 PE=3 SV=1 | A0A8I3S7P0 | *DDX17* | 80 kDa | 0.1 | 1 | 1.272794935 | 0.348 |
| **57** | phosphopyruvate hydratase OS=Canis lupus familiaris OX=9615 GN=ENO3 PE=3 SV=1 | A0A8I3MKD5 | *ENO3* | 48 kDa | 0.1 | 1 | 1.346300069 | 0.429 |
| **58** | Spectrin alpha, non-erythrocytic 1 OS=Canis lupus familiaris OX=9615 GN=SPTAN1 PE=3 SV=1 | A0A8I3S6F7 | *SPTAN1* | 285 kDa | 0.1 | 1 | 1.400556321 | 0.486 |
| **59** | Collagen type XII alpha 1 chain OS=Canis lupus familiaris OX=9615 GN=COL12A1 PE=4 SV=1 | A0A8I3NP07 | *COL12A1* | 324 kDa | 0.11 | 0.9586073 | 0.647970483 | -0.626 |
| **60** | PTTG1 interacting protein OS=Canis lupus familiaris OX=9615 GN=LOC100856547 PE=4 SV=1 | A0A8I3PCI0 | *LOC100856547* | 12 kDa | 0.11 | 0.9586073 | 0.795536484 | -0.33 |
| **61** | Chaperonin containing TCP1 subunit 6B OS=Canis lupus familiaris OX=9615 GN=CCT6B PE=3 SV=1 | A0A8I3NGT6 | *CCT6B* | 54 kDa | 0.11 | 0.9586073 | 1.272794935 | 0.348 |
| **62** | Serine/threonine kinase receptor associated protein OS=Canis lupus familiaris OX=9615 GN=STRAP PE=4 SV=1 | A0A8I3PA92 | *STRAP* | 38 kDa | 0.11 | 0.9586073 | 1.363202607 | 0.447 |
| **63** | Histone H2A/H2B/H3 domain-containing protein OS=Canis lupus familiaris OX=9615 PE=3 SV=1 | A0A8I3PS44 | | 29 kDa | 0.11 | 0.9586073 | 1.591072968 | 0.67 |
| **64** | Vasodilator stimulated phosphoprotein OS=Canis lupus familiaris OX=9615 GN=VASP PE=3 SV=1 | A0A8I3MWS4 | *VASP* | 59 kDa | 0.11 | 0.9586073 | 1.666706414 | 0.737 |
| **65** | Proteasome subunit alpha type OS=Canis lupus familiaris OX=9615 GN=PSMA7 PE=3 SV=1 | A0A8I3NW81 | *PSMA7* | 28 kDa | 0.12 | 0.9208188 | 0.530343871 | -0.915 |
| **66** | Cofilin 1 OS=Canis lupus familiaris OX=9615 GN=CFL1 PE=3 SV=1 | A0A8I3S096 | *CFL1* | 17 kDa | 0.12 | 0.9208188 | 0.760489377 | -0.395 |
| **67** | C-1-tetrahydrofolate synthase, cytoplasmic OS=Canis lupus familiaris OX=9615 GN=MTHFD1 PE=3 SV=1 | A0A8I3MJJ7 | *MTHFD1* | 101 kDa | 0.12 | 0.9208188 | 0.795536484 | -0.33 |
| **68** | SPARC OS=Canis lupus familiaris OX=9615 GN=SPARC PE=3 SV=1 | A0A8I3MWF4 | *SPARC* | 37 kDa | 0.12 | 0.9208188 | 1.396678532 | 0.482 |
| **69** | Heparan sulfate proteoglycan 2 OS=Canis lupus familiaris OX=9615 GN=HSPG2 PE=4 SV=1 | A0A8I3MGU2 | *HSPG2* | 444 kDa | 0.12 | 0.9208188 | 1.43893358 | 0.525 |
| **70** | Ribosome binding protein 1 OS=Canis lupus familiaris OX=9615 GN=RRBP1 PE=4 SV=1 | A0A8I3PD78 | *RRBP1* | 164 kDa | 0.13 | 0.8860566 | 0.750019495 | -0.415 |

| | | | | | | | | | |
|---|---|---|---|---|---|---|---|---|---|
| **71** | Methionine aminopeptidase 2 OS=Canis lupus familiaris OX=9615 GN=METAP2 PE=3 SV=1 | A0A8I3S7G5 | *METAP2* | 49 kDa | 0.13 | 0.8860566 | 0.795536484 | -0.33 |
| **72** | Calcyclin-binding protein OS=Canis lupus familiaris OX=9615 GN=CACYBP PE=4 SV=1 | A0A8I3RY44 | *CACYBP* | 41 kDa | 0.13 | 0.8860566 | 0.795536484 | -0.33 |
| **73** | Eukaryotic translation elongation factor 1 delta OS=Canis lupus familiaris OX=9615 GN=EEF1D PE=3 SV=1 | A0A8I3RYX3 | *EEF1D* | 44 kDa | 0.13 | 0.8860566 | 0.842062954 | -0.248 |
| **74** | Heat shock protein family A (Hsp70) member 8 OS=Canis lupus familiaris OX=9615 GN=HSPA8 PE=3 SV=1 | A0A8I3NK47 | *HSPA8* | 71 kDa | 0.13 | 0.8860566 | 0.866336856 | -0.207 |
| **75** | Uncharacterized protein OS=Canis lupus familiaris OX=9615 GN=PTX3 PE=4 SV=1 | A0A8I3PKQ2 | *PTX3* | 38 kDa | 0.13 | 0.8860566 | 1.514666316 | 0.599 |
| **76** | Eukaryotic translation initiation factor 3 subunit G OS=Canis lupus familiaris OX=9615 GN=EIF3G PE=3 SV=1 | A0A8I3P751 | *EIF3G* | 36 kDa | 0.13 | 0.8860566 | 1.767855062 | 0.822 |
| **77** | Uncharacterized protein OS=Canis lupus familiaris OX=9615 GN=LOC100856351 PE=3 SV=1 | A0A8I3P2J9 | *LOC100856351* | 16 kDa | 0.13 | 0.8860566 | 1.855746953 | 0.892 |
| **78** | Heterogeneous nuclear ribonucleoprotein U OS=Canis lupus familiaris OX=9615 GN=HNRNPU PE=4 SV=1 | A0A8I3MTZ6 | *HNRNPU* | 61 kDa | 0.13 | 0.8860566 | 1.855746953 | 0.892 |
| **79** | Dipeptidyl peptidase 1 OS=Canis lupus familiaris OX=9615 GN=CTSC PE=3 SV=1 | A0A8I3S5V2 | *CTSC* | 52 kDa | 0.13 | 0.8860566 | 2.114036081 | 1.08 |
| **80** | 40S ribosomal protein S25 OS=Canis lupus familiaris OX=9615 GN=RPS25 PE=3 SV=1 | A0A8I3RR71 | *RPS25* | 14 kDa | 0.13 | 0.8860566 | 2.114036081 | 1.08 |
| **81** | Vesicle amine transport 1 OS=Canis lupus familiaris OX=9615 GN=VAT1 PE=3 SV=1 | A0A8I3RT77 | *VAT1* | 43 kDa | 0.14 | 0.853872 | 0.720964436 | -0.472 |
| **82** | Galectin 1 OS=Canis lupus familiaris OX=9615 GN=LGALS1 PE=4 SV=1 | A0A8I3N184 | *LGALS1* | 14 kDa | 0.14 | 0.853872 | 0.837987135 | -0.255 |
| **83** | DNA-(apurinic or apyrimidinic site) endonuclease OS=Canis lupus familiaris OX=9615 GN=APEX1 PE=3 SV=1 | A0A8I3PLA3 | *APEX1* | 75 kDa | 0.14 | 0.853872 | 1.193335743 | 0.255 |
| **84** | Cellular communication network factor 1 OS=Canis lupus familiaris OX=9615 GN=CCN1 PE=3 SV=1 | A0A8I3S8V0 | *CCN1* | 42 kDa | 0.14 | 0.853872 | 1.378405153 | 0.463 |
| **85** | Heterogeneous nuclear ribonucleoprotein H2 OS=Canis lupus familiaris OX=9615 GN=HNRNPH2 PE=4 SV=1 | A0A8I3P7A3 | *HNRNPH2* | 49 kDa | 0.15 | 0.8239087 | 0.469761375 | -1.09 |
| **86** | Phosphoglycerate kinase OS=Canis lupus familiaris OX=9615 GN=PGK2 PE=3 SV=1 | A0A8I3N4J1 | *PGK2* | 47 kDa | 0.15 | 0.8239087 | 0.636397468 | -0.652 |
| **87** | Splicing factor 3b subunit 4 OS=Canis lupus familiaris OX=9615 GN=SF3B4 PE=3 SV=1 | A0A8I3NK96 | *SF3B4* | 44 kDa | 0.15 | 0.8239087 | 0.707106781 | -0.5 |
| **88** | Large ribosomal subunit protein eL34 OS=Canis lupus familiaris OX=9615 GN=LOC111096443 PE=3 SV=1 | A0A8I3RT15 | *LOC111096443* | 13 kDa | 0.15 | 0.8239087 | 1.0604672 | 0.0847 |
| **89** | Aspartate aminotransferase OS=Canis lupus familiaris OX=9615 GN=GOT2 PE=3 SV=1 | A0A8I3MPF7 | *GOT2* | 54 kDa | 0.15 | 0.8239087 | 1.484523571 | 0.57 |
| **90** | Collapsin response mediator protein 1 OS=Canis lupus familiaris OX=9615 GN=CRMP1 PE=3 SV=1 | A0A8I3MRF4 | *CRMP1* | 74 kDa | 0.15 | 0.8239087 | 1.591072968 | 0.67 |
| **91** | Cell cycle associated protein 1 OS=Canis lupus familiaris OX=9615 GN=CAPRIN1 PE=3 SV=1 | A0A8I3S8S8 | *CAPRIN1* | 79 kDa | 0.16 | 0.79588 | 0.386891248 | -1.37 |
| **92** | Malate dehydrogenase 1 OS=Canis lupus familiaris OX=9615 GN=MDH1 PE=4 SV=1 | A0A8I3P8L3 | *MDH1* | 23 kDa | 0.16 | 0.79588 | 0.757333158 | -0.401 |
| **93** | Ubiquitin-like domain-containing protein OS=Canis lupus familiaris OX=9615 PE=4 SV=1 | A0A8I3NUE4 | | 22 kDa | 0.16 | 0.79588 | 0.827596816 | -0.273 |
| **94** | Phosphoinositide phospholipase C OS=Canis lupus familiaris OX=9615 GN=PLCD1 PE=4 SV=1 | A0A8I3Q2Z2 | *PLCD1* | 91 kDa | 0.16 | 0.79588 | 1.0604672 | 0.0847 |

| | | | | | | | | |
|---|---|---|---|---|---|---|---|---|
| **95** | T-complex protein 1 subunit eta OS=Canis lupus familiaris OX=9615 GN=LOC100856782 PE=3 SV=1 | A0A8I3S301 | *LOC100856782* | 59 kDa | 0.17 | 0.7695511 | 0.544498508 | -0.877 |
| **96** | Heat shock 70 kDa protein 4 OS=Canis lupus familiaris OX=9615 GN=HSPA4 PE=3 SV=1 | A0A8I3N9R4 | *HSPA4* | 94 kDa | 0.17 | 0.7695511 | 0.717474767 | -0.479 |
| **97** | Biglycan OS=Canis lupus familiaris OX=9615 GN=BGN PE=3 SV=1 | A0A8I3PHY6 | *BGN* | 42 kDa | 0.17 | 0.7695511 | 1.227735684 | 0.296 |
| **98** | Fibrillin 2 OS=Canis lupus familiaris OX=9615 GN=FBN2 PE=3 SV=1 | A0A8I3NQU3 | *FBN2* | 314 kDa | 0.17 | 0.7695511 | 1.391846392 | 0.477 |
| **99** | Myotrophin OS=Canis lupus familiaris OX=9615 GN=MTPN PE=3 SV=3 | Q863Z4 | *MTPN* | 13 kDa | 0.17 | 0.7695511 | 1.414213562 | 0.5 |
| **100** | Beta-2-glycoprotein 1 OS=Canis lupus familiaris OX=9615 GN=APOH PE=2 SV=1 | P33703 | *APOH* | 38 kDa | 0.17 | 0.7695511 | 2.114036081 | 1.08 |
| **101** | Rho GDP dissociation inhibitor alpha OS=Canis lupus familiaris OX=9615 GN=ARHGDIA PE=3 SV=1 | A0A8I3N6C6 | *ARHGDIA* | 18 kDa | 0.18 | 0.7447275 | 0.762072415 | -0.392 |
| **102** | glutaminyl-peptide cyclotransferase OS=Canis lupus familiaris OX=9615 GN=QPCT PE=3 SV=1 | A0A8I3PHH6 | *QPCT* | 41 kDa | 0.18 | 0.7447275 | 1.0604672 | 0.0847 |
| **103** | Uncharacterized protein OS=Canis lupus familiaris OX=9615 PE=4 SV=1 | A0A8I3S8W0 | | 8 kDa | 0.18 | 0.7447275 | 1.272794935 | 0.348 |
| **104** | Ferritin OS=Canis lupus familiaris OX=9615 PE=3 SV=1 | A0A8I3NVK3 | | 32 kDa | 0.18 | 0.7447275 | 1.514666316 | 0.599 |
| **105** | Large ribosomal subunit protein uL5 OS=Canis lupus familiaris OX=9615 GN=ELOA PE=3 SV=1 | A0A8I3NWE4 | *ELOA* | 25 kDa | 0.18 | 0.7447275 | 2.29739671 | 1.2 |
| **106** | Coronin OS=Canis lupus familiaris OX=9615 GN=CORO1B PE=3 SV=1 | A0A8I3NE74 | *CORO1B* | 54 kDa | 0.19 | 0.7212464 | 0.423372656 | -1.24 |
| **107** | FERM domain containing kindlin 2 OS=Canis lupus familiaris OX=9615 GN=FERMT2 PE=3 SV=1 | A0A8I3MV64 | *FERMT2* | 79 kDa | 0.19 | 0.7212464 | 0.675018993 | -0.567 |
| **108** | Large ribosomal subunit protein P2 OS=Canis lupus familiaris OX=9615 GN=RPLP2 PE=3 SV=1 | A0A8I3N5I3 | *RPLP2* | 12 kDa | 0.19 | 0.7212464 | 0.675018993 | -0.567 |
| **109** | Cartilage acidic protein 1 OS=Canis lupus familiaris OX=9615 GN=CRTAC1 PE=4 SV=1 | A0A8I3Q959 | *CRTAC1* | 67 kDa | 0.19 | 0.7212464 | 1.0604672 | 0.0847 |
| **110** | Follistatin like 1 OS=Canis lupus familiaris OX=9615 GN=FSTL1 PE=4 SV=1 | A0A8I3S0F8 | *FSTL1* | 58 kDa | 0.19 | 0.7212464 | 1.178539408 | 0.237 |
| **111** | Alpha-2-macroglobulin OS=Canis lupus familiaris OX=9615 GN=A2M PE=3 SV=1 | A0A8I3QH15 | *A2M* | 164 kDa | 0.19 | 0.7212464 | 1.42899414 | 0.515 |
| **112** | Aspartate aminotransferase OS=Canis lupus familiaris OX=9615 GN=GOT1 PE=3 SV=1 | A0A8I3QCW7 | *GOT1* | 46 kDa | 0.19 | 0.7212464 | 1.549711862 | 0.632 |
| **113** | Dystroglycan 1 OS=Canis lupus familiaris OX=9615 GN=DAG1 PE=3 SV=1 | Q9TSZ6 | *DAG1* | 97 kDa | 0.19 | 0.7212464 | 1.591072968 | 0.67 |
| **114** | Acyl-CoA binding domain containing 3 OS=Canis lupus familiaris OX=9615 GN=ACBD3 PE=4 SV=1 | A0A8I3N0K8 | *ACBD3* | 60 kDa | 0.2 | 0.69897 | 0.303548721 | -1.72 |
| **115** | Proteasome 20S subunit alpha 3 OS=Canis lupus familiaris OX=9615 GN=PSMA3 PE=3 SV=1 | A0A8I3PDR8 | *PSMA3* | 29 kDa | 0.2 | 0.69897 | 0.386891248 | -1.37 |
| **116** | Tubulin beta chain OS=Canis lupus familiaris OX=9615 GN=TUBB PE=3 SV=1 | A0A8I3N467 | *TUBB* | 48 kDa | 0.2 | 0.69897 | 0.60583633 | -0.723 |
| **117** | Nucleophosmin 1 OS=Canis lupus familiaris OX=9615 GN=NPM1 PE=3 SV=1 | A0A8I3MH65 | *NPM1* | 28 kDa | 0.2 | 0.69897 | 0.645281245 | -0.632 |
| **118** | Ribosomal protein L19 OS=Canis lupus familiaris OX=9615 GN=RPL19 PE=3 SV=1 | A0A8I3NQB4 | *RPL19* | 23 kDa | 0.2 | 0.69897 | 0.66296288 | -0.593 |

| | | | | | | | | |
|---|---|---|---|---|---|---|---|---|
| **119** | Uncharacterized protein OS=Canis lupus familiaris OX=9615 PE=4 SV=1 | E2R5M5 | | 23 kDa | 0.2 | 0.69897 | 0.675018993 | -0.567 |
| **120** | Complement C2 OS=Canis lupus familiaris OX=9615 GN=CFB PE=4 SV=1 | A0A8I3NLH3 | *CFB* | 86 kDa | 0.2 | 0.69897 | 1.0604672 | 0.0847 |
| **121** | Caveolae associated protein 1 OS=Canis lupus familiaris OX=9615 GN=CAVIN1 PE=3 SV=1 | A0A8I3NAW5 | *CAVIN1* | 43 kDa | 0.2 | 0.69897 | 1.21167266 | 0.277 |
| **122** | Insulin-like growth factor II OS=Canis lupus familiaris OX=9615 GN=IGF2 PE=3 SV=1 | A0A8I3RYD4 | *IGF2* | 31 kDa | 0.2 | 0.69897 | 2.114036081 | 1.08 |
| **123** | Transferrin-like domain-containing protein OS=Canis lupus familiaris OX=9615 GN=LOC477072 PE=3 SV=1 | A0A8I3Q5K3 | *LOC477072* | 78 kDa | 0.2 | 0.69897 | 3.810551992 | 1.93 |
| **124** | Carboxypeptidase OS=Canis lupus familiaris OX=9615 GN=SCPEP1 PE=3 SV=1 | A0A8I3NVI7 | *SCPEP1* | 50 kDa | 0.21 | 0.6777807 | 0.234880687 | -2.09 |
| **125** | Collagen type VI alpha 3 chain OS=Canis lupus familiaris OX=9615 GN=COL6A3 PE=4 SV=1 | A0A8I3Q026 | *COL6A3* | 343 kDa | 0.21 | 0.6777807 | 0.309926925 | -1.69 |
| **126** | Interleukin enhancer binding factor 2 OS=Canis lupus familiaris OX=9615 GN=ILF2 PE=4 SV=1 | A0A8I3MGS3 | *ILF2* | 43 kDa | 0.21 | 0.6777807 | 0.578344092 | -0.79 |
| **127** | Osteoglycin OS=Canis lupus familiaris OX=9615 GN=OGN PE=3 SV=1 | A0A8I3PS70 | *OGN* | 42 kDa | 0.21 | 0.6777807 | 0.60583633 | -0.723 |
| **128** | Uncharacterized protein OS=Canis lupus familiaris OX=9615 GN=SERBP1 PE=3 SV=1 | A0A8I3QC63 | *SERBP1* | 45 kDa | 0.21 | 0.6777807 | 0.62981499 | -0.667 |
| **129** | Mitogen-activated protein kinase OS=Canis lupus familiaris OX=9615 GN=MAPK1 PE=3 SV=1 | A0A8I3PZP0 | *MAPK1* | 41 kDa | 0.21 | 0.6777807 | 1.0604672 | 0.0847 |
| **130** | IQ motif containing GTPase activating protein 1 OS=Canis lupus familiaris OX=9615 GN=IQGAP1 PE=4 SV=1 | A0A8I3MM93 | *IQGAP1* | 136 kDa | 0.21 | 0.6777807 | 1.299539062 | 0.378 |
| **131** | CD109 molecule OS=Canis lupus familiaris OX=9615 GN=CD109 PE=3 SV=1 | A0A5F4D8V4 | *CD109* | 176 kDa | 0.21 | 0.6777807 | 1.354724977 | 0.438 |
| **132** | Clusterin OS=Canis lupus familiaris OX=9615 GN=CLU PE=3 SV=1 | A0A8I3NF26 | *CLU* | 53 kDa | 0.21 | 0.6777807 | 1.393777239 | 0.479 |
| **133** | Carboxypeptidase E OS=Canis lupus familiaris OX=9615 GN=CPE PE=3 SV=1 | A0A8I3N249 | *CPE* | 74 kDa | 0.21 | 0.6777807 | 1.855746953 | 0.892 |
| **134** | Alpha fetoprotein OS=Canis lupus familiaris OX=9615 GN=AFP PE=4 SV=1 | A0A8I3N1E6 | *AFP* | 69 kDa | 0.21 | 0.6777807 | 1.933212194 | 0.951 |
| **135** | Cartilage oligomeric matrix protein OS=Canis lupus familiaris OX=9615 GN=COMP PE=3 SV=1 | A0A8I3P787 | *COMP* | 82 kDa | 0.21 | 0.6777807 | 2.657371628 | 1.41 |
| **136** | REVERSE_A0A8I3NPQ5 | REVERSE_A0A8I3NPQ5 | | | 0.21 | 0.6777807 | 2.828427125 | 1.5 |
| **137** | Afamin OS=Canis lupus familiaris OX=9615 GN=AFM PE=4 SV=1 | A0A8I3NCZ8 | *AFM* | 69 kDa | 0.21 | 0.6777807 | 3.182145935 | 1.67 |
| **138** | COP9 signalosome complex subunit 4 OS=Canis lupus familiaris OX=9615 GN=COPS4 PE=3 SV=1 | A0A8I3P5Y3 | *COPS4* | 46 kDa | 0.22 | 0.6575773 | 0.26425451 | -1.92 |
| **139** | Dynactin subunit 1 OS=Canis lupus familiaris OX=9615 GN=DCTN1 PE=3 SV=1 | A0A8I3P3A5 | *DCTN1* | 140 kDa | 0.22 | 0.6575773 | 0.530343871 | -0.915 |
| **140** | Aldo-keto reductase family 1 member B OS=Canis lupus familiaris OX=9615 GN=AKR1B1 PE=3 SV=1 | A0A8I3P2L6 | *AKR1B1* | 36 kDa | 0.22 | 0.6575773 | 0.578344092 | -0.79 |
| **141** | Glycine--tRNA ligase OS=Canis lupus familiaris OX=9615 GN=GARS1 PE=3 SV=1 | A0A8I3S467 | *GARS1* | 89 kDa | 0.22 | 0.6575773 | 0.717474767 | -0.479 |
| **142** | 6-phosphogluconolactonase OS=Canis lupus familiaris OX=9615 GN=PGLS PE=3 SV=1 | A0A8I3SAK1 | *PGLS* | 27 kDa | 0.22 | 0.6575773 | 0.848507902 | -0.237 |

| | | | | | | | | |
|---|---|---|---|---|---|---|---|---|
| **143** | glutamate--tRNA ligase OS=Canis lupus familiaris OX=9615 GN=EPRS1 PE=3 SV=1 | A0A8I3Q110 | *EPRS1* | 163 kDa | 0.22 | 0.6575773 | 0.94258851 | -0.0853 |
| **144** | Protein AMBP OS=Canis lupus familiaris OX=9615 GN=AMBP PE=3 SV=1 | A0A8I3NDM2 | *AMBP* | 54 kDa | 0.22 | 0.6575773 | 1.0604672 | 0.0847 |
| **145** | Vimentin OS=Canis lupus familiaris OX=9615 GN=VIM PE=3 SV=1 | A0A8I3NU02 | *VIM* | 54 kDa | 0.22 | 0.6575773 | 1.35754498 | 0.441 |
| **146** | Procollagen C-endopeptidase enhancer OS=Canis lupus familiaris OX=9615 GN=PCOLCE PE=4 SV=1 | A0A8I3MFU6 | *PCOLCE* | 52 kDa | 0.22 | 0.6575773 | 1.391846392 | 0.477 |
| **147** | procollagen-lysine 5-dioxygenase OS=Canis lupus familiaris OX=9615 GN=PLOD1 PE=4 SV=1 | A0A8I3RRS2 | *PLOD1* | 103 kDa | 0.22 | 0.6575773 | 1.591072968 | 0.67 |
| **148** | Carboxypeptidase E OS=Canis lupus familiaris OX=9615 GN=CPE PE=3 SV=1 | A0A8I3N117 | *CPE* | 53 kDa | 0.22 | 0.6575773 | 1.767855062 | 0.822 |
| **149** | High mobility group AT-hook 1 OS=Canis lupus familiaris OX=9615 GN=HMGA1 PE=3 SV=1 | A0A8I3SA81 | *HMGA1* | 10 kDa | 0.22 | 0.6575773 | 2.828427125 | 1.5 |
| **150** | Plasminogen OS=Canis lupus familiaris OX=9615 GN=PLG PE=3 SV=1 | A0A8I3MF27 | *PLG* | 91 kDa | 0.22 | 0.6575773 | 3.182145935 | 1.67 |
| **151** | Early endosome antigen 1 OS=Canis lupus familiaris OX=9615 GN=EEA1 PE=4 SV=1 | A0A8I3PCC1 | *EEA1* | 163 kDa | 0.23 | 0.6382722 | 0.482968164 | -1.05 |
| **152** | protein-synthesizing GTPase OS=Canis lupus familiaris OX=9615 GN=EIF2S3 PE=4 SV=1 | A0A8I3Q3Z7 | *EIF2S3* | 67 kDa | 0.23 | 0.6382722 | 0.51192345 | -0.966 |
| **153** | Staphylococcal nuclease domain-containing protein 1 OS=Canis lupus familiaris OX=9615 GN=SND1 PE=4 SV=1 | A0A8I3PQZ2 | *SND1* | 95 kDa | 0.23 | 0.6382722 | 0.596667872 | -0.745 |
| **154** | Dermatopontin OS=Canis lupus familiaris OX=9615 GN=DPT PE=3 SV=1 | A0A8I3RS96 | *DPT* | 24 kDa | 0.23 | 0.6382722 | 0.636397468 | -0.652 |
| **155** | Heat shock protein 90 alpha family class B member 1 OS=Canis lupus familiaris OX=9615 GN=HSP90AB1 PE=3 SV=1 | A0A8I3N9V3 | *HSP90AB1* | 80 kDa | 0.23 | 0.6382722 | 0.883927531 | -0.178 |
| **156** | Inter-alpha-trypsin inhibitor heavy chain 3 OS=Canis lupus familiaris OX=9615 GN=ITIH3 PE=3 SV=1 | A0A8I3QUR2 | *ITIH3* | 100 kDa | 0.23 | 0.6382722 | 1.178539408 | 0.237 |
| **157** | Fibronectin 1 OS=Canis lupus familiaris OX=9615 GN=FN1 PE=4 SV=1 | A0A8I3PQD5 | *FN1* | 262 kDa | 0.23 | 0.6382722 | 1.450952208 | 0.537 |
| **158** | Alpha-2-HS-glycoprotein OS=Canis lupus familiaris OX=9615 GN=AHSG PE=4 SV=1 | A0A8I3PIK8 | *AHSG* | 39 kDa | 0.23 | 0.6382722 | 2.02791896 | 1.02 |
| **159** | Vitamin D-binding protein OS=Canis lupus familiaris OX=9615 GN=GC PE=4 SV=1 | A0A8I3RWX3 | *GC* | 55 kDa | 0.23 | 0.6382722 | 2.329467173 | 1.22 |
| **160** | Fetuin B OS=Canis lupus familiaris OX=9615 GN=FETUB PE=4 SV=1 | A0A8I3PDJ5 | *FETUB* | 42 kDa | 0.23 | 0.6382722 | 2.37841423 | 1.25 |
| **161** | Aspartyl aminopeptidase OS=Canis lupus familiaris OX=9615 GN=DNPEP PE=3 SV=1 | A0A8I3QAI6 | *DNPEP* | 55 kDa | 0.24 | 0.6197888 | 0.423372656 | -1.24 |
| **162** | Tripeptidyl-peptidase 1 OS=Canis lupus familiaris OX=9615 GN=TPP1 PE=4 SV=1 | A0A8I3NAI6 | *TPP1* | 51 kDa | 0.24 | 0.6197888 | 0.530343871 | -0.915 |
| **163** | Rho GTPase activating protein 1 OS=Canis lupus familiaris OX=9615 GN=ARHGAP1 PE=4 SV=1 | A0A8I3PTG9 | *ARHGAP1* | 50 kDa | 0.24 | 0.6197888 | 0.558256481 | -0.841 |
| **164** | Rab GDP dissociation inhibitor OS=Canis lupus familiaris OX=9615 GN=GDI2 PE=3 SV=1 | A0A8I3N2F9 | *GDI2* | 45 kDa | 0.24 | 0.6197888 | 0.623732786 | -0.681 |
| **165** | 60S acidic ribosomal protein P0 OS=Canis lupus familiaris OX=9615 GN=RPLP0 PE=3 SV=1 | A0A8I3QJZ3 | *RPLP0* | 34 kDa | 0.24 | 0.6197888 | 0.62676651 | -0.674 |
| **166** | Switching B cell complex subunit SWAP70 OS=Canis lupus familiaris OX=9615 GN=SWAP70 PE=4 SV=1 | A0A8I3NS08 | *SWAP70* | 62 kDa | 0.24 | 0.6197888 | 0.636397468 | -0.652 |

| | | | | | | | | |
|---|---|---|---|---|---|---|---|---|
| **167** | Actin-related protein 2/3 complex subunit OS=Canis lupus familiaris OX=9615 GN=ARPC1B PE=3 SV=1 | A0A8I3MVD4 | *ARPC1B* | 41 kDa | 0.24 | 0.6197888 | 0.707106781 | -0.5 |
| **168** | TATA-box binding protein associated factor 15 OS=Canis lupus familiaris OX=9615 GN=TAF15 PE=3 SV=1 | A0A8I3NBU0 | *TAF15* | 57 kDa | 0.24 | 0.6197888 | 0.707106781 | -0.5 |
| **169** | DExD-box helicase 39A OS=Canis lupus familiaris OX=9615 GN=DDX39A PE=4 SV=1 | A0A8I3PGD1 | *DDX39A* | 49 kDa | 0.24 | 0.6197888 | 0.795536484 | -0.33 |
| **170** | G1 to S phase transition 1 OS=Canis lupus familiaris OX=9615 GN=GSPT1 PE=3 SV=1 | A0A8I3NLG0 | *GSPT1* | 72 kDa | 0.24 | 0.6197888 | 1.0604672 | 0.0847 |
| **171** | Diazepam binding inhibitor, acyl-CoA binding protein OS=Canis lupus familiaris OX=9615 GN=DBI PE=4 SV=1 | A0A8I3QQH5 | *DBI* | 21 kDa | 0.24 | 0.6197888 | 1.193335743 | 0.255 |
| **172** | Apoptosis inhibitor 5 OS=Canis lupus familiaris OX=9615 GN=API5 PE=3 SV=1 | A0A8I3S176 | *API5* | 58 kDa | 0.24 | 0.6197888 | 1.272794935 | 0.348 |
| **173** | EH domain containing 2 OS=Canis lupus familiaris OX=9615 GN=EHD2 PE=4 SV=1 | A0A8I3MKL5 | *EHD2* | 61 kDa | 0.24 | 0.6197888 | 1.591072968 | 0.67 |
| **174** | Protein phosphatase 1 regulatory subunit OS=Canis lupus familiaris OX=9615 GN=PPP1R12A PE=4 SV=1 | A0A8I3N5P7 | *PPP1R12A* | 122 kDa | 0.24 | 0.6197888 | 1.767855062 | 0.822 |
| **175** | ATP-dependent RNA helicase OS=Canis lupus familiaris OX=9615 GN=EIF4A1 PE=3 SV=1 | A0A8I3PVR6 | *EIF4A1* | 44 kDa | 0.25 | 0.60206 | 0.26425451 | -1.92 |
| **176** | Ubiquitin conjugating enzyme E2 K OS=Canis lupus familiaris OX=9615 GN=UBE2K PE=4 SV=1 | A0A8I3MSC8 | *UBE2K* | 23 kDa | 0.25 | 0.60206 | 0.530343871 | -0.915 |
| **177** | Heterogeneous nuclear ribonucleoprotein A1 OS=Canis lupus familiaris OX=9615 GN=HNRNPA1 PE=4 SV=1 | A0A8I3P181 | *HNRNPA1* | 39 kDa | 0.25 | 0.60206 | 0.567227742 | -0.818 |
| **178** | Slit guidance ligand 3 OS=Canis lupus familiaris OX=9615 GN=SLIT3 PE=4 SV=1 | A0A8I3MRE2 | *SLIT3* | 168 kDa | 0.25 | 0.60206 | 0.636397468 | -0.652 |
| **179** | Microtubule-associated protein OS=Canis lupus familiaris OX=9615 GN=MAP4 PE=4 SV=1 | A0A8I3RVJ6 | *MAP4* | 238 kDa | 0.25 | 0.60206 | 0.729004689 | -0.456 |
| **180** | Osteomodulin OS=Canis lupus familiaris OX=9615 GN=OMD PE=4 SV=1 | A0A8I3S4Y4 | *OMD* | 48 kDa | 0.25 | 0.60206 | 0.795536484 | -0.33 |
| **181** | Actin related protein 3 OS=Canis lupus familiaris OX=9615 GN=ACTR3 PE=3 SV=1 | A0A8I3NEW9 | *ACTR3* | 67 kDa | 0.25 | 0.60206 | 1.295940965 | 0.374 |
| **182** | Chloride intracellular channel protein OS=Canis lupus familiaris OX=9615 PE=3 SV=1 | A0A8I3MPJ5 | | 26 kDa | 0.25 | 0.60206 | 1.484523571 | 0.57 |
| **183** | Fibroblast activation protein alpha OS=Canis lupus familiaris OX=9615 GN=FAP PE=4 SV=1 | A0A8I3S7K0 | *FAP* | 85 kDa | 0.26 | 0.5850267 | 0.386891248 | -1.37 |
| **184** | Nuclear migration protein nudC OS=Canis lupus familiaris OX=9615 GN=NUDC PE=3 SV=1 | A0A8I3N734 | *NUDC* | 38 kDa | 0.26 | 0.5850267 | 0.435275282 | -1.2 |
| **185** | Ribosomal protein S5 C-terminal domain-containing protein OS=Canis lupus familiaris OX=9615 PE=4 SV=1 | A0A8I3RPR6 | | 9 kDa | 0.26 | 0.5850267 | 0.66296288 | -0.593 |
| **186** | Actinin alpha 4 OS=Canis lupus familiaris OX=9615 GN=LGALS7B PE=4 SV=1 | A0A8I3RTY1 | *LGALS7B* | 109 kDa | 0.26 | 0.5850267 | 0.868140228 | -0.204 |
| **187** | Serpin family F member 1 OS=Canis lupus familiaris OX=9615 GN=SERPINF1 PE=3 SV=1 | A0A8I3S0B7 | *SERPINF1* | 46 kDa | 0.26 | 0.5850267 | 1.20664392 | 0.271 |
| **188** | IF rod domain-containing protein OS=Canis lupus familiaris OX=9615 GN=KRT7 PE=3 SV=1 | A0A8I3PRA8 | *KRT7* | 51 kDa | 0.26 | 0.5850267 | 1.249196126 | 0.321 |
| **189** | Albumin OS=Canis lupus familiaris OX=9615 GN=ALB PE=4 SV=1 | A0A8I3MZG8 | *ALB* | 47 kDa | 0.26 | 0.5850267 | 1.710004356 | 0.774 |
| **190** | Uncharacterized protein OS=Canis lupus familiaris OX=9615 GN=SPON2 PE=4 SV=1 | A0A8I3MD18 | *SPON2* | 37 kDa | 0.27 | 0.5686362 | 0.332171454 | -1.59 |

| | | | | | | | | |
|---|---|---|---|---|---|---|---|---|
| **191** | Biliverdin reductase B OS=Canis lupus familiaris OX=9615 GN=BLVRB PE=4 SV=1 | A0A8I3MIS0 | *BLVRB* | 26 kDa | 0.27 | 0.5686362 | 0.530343871 | -0.915 |
| **192** | Endoplasmic reticulum oxidoreductase 1 alpha OS=Canis lupus familiaris OX=9615 GN=ERO1A PE=3 SV=1 | A0A8I3MRA0 | *ERO1A* | 72 kDa | 0.27 | 0.5686362 | 0.636397468 | -0.652 |
| **193** | Collagen type VI alpha 2 chain OS=Canis lupus familiaris OX=9615 GN=COL6A2 PE=4 SV=1 | A0A8I3PM54 | *COL6A2* | 106 kDa | 0.27 | 0.5686362 | 0.832198735 | -0.265 |
| **194** | peptidylprolyl isomerase OS=Canis lupus familiaris OX=9615 GN=FKBP3 PE=4 SV=1 | A0A8I3MMG4 | *FKBP3* | 25 kDa | 0.27 | 0.5686362 | 1.0604672 | 0.0847 |
| **195** | Prostaglandin reductase 1 OS=Canis lupus familiaris OX=9615 GN=PTGR1 PE=3 SV=1 | A0A8I3MRC0 | *PTGR1* | 36 kDa | 0.27 | 0.5686362 | 1.237132479 | 0.307 |
| **196** | Uncharacterized protein OS=Canis lupus familiaris OX=9615 GN=TMPO PE=3 SV=1 | A0A8I3PQ05 | *TMPO* | 46 kDa | 0.27 | 0.5686362 | 1.414213562 | 0.5 |
| **197** | TIMP metallopeptidase inhibitor 1 OS=Canis lupus familiaris OX=9615 GN=TIMP1 PE=3 SV=1 | A0A8I3Q4H4 | *TIMP1* | 28 kDa | 0.27 | 0.5686362 | 1.643760375 | 0.717 |
| **198** | A-kinase anchoring protein 12 OS=Canis lupus familiaris OX=9615 GN=AKAP12 PE=4 SV=1 | A0A8I3PFX0 | *AKAP12* | 180 kDa | 0.28 | 0.552842 | 0.647970483 | -0.626 |
| **199** | Non-metastatic cells 1, protein (NM23A) expressed in OS=Canis lupus familiaris OX=9615 GN=NME1 PE=3 SV=1 | A0A8I3NVX7 | *NME1* | 10 kDa | 0.28 | 0.552842 | 0.765778999 | -0.385 |
| **200** | Elongation factor 1-gamma OS=Canis lupus familiaris OX=9615 PE=4 SV=1 | A0A8I3RTB2 | | 59 kDa | 0.28 | 0.552842 | 0.829319546 | -0.27 |
| **201** | Uncharacterized protein OS=Canis lupus familiaris OX=9615 GN=CSRP1 PE=4 SV=1 | A0A8I3P401 | *CSRP1* | 22 kDa | 0.28 | 0.552842 | 0.857376037 | -0.222 |
| **202** | Pyruvate kinase OS=Canis lupus familiaris OX=9615 GN=PKM PE=3 SV=1 | A0A8I3PG67 | *PKM* | 58 kDa | 0.28 | 0.552842 | 0.883927531 | -0.178 |
| **203** | Inter-alpha-trypsin inhibitor heavy chain 4 OS=Canis lupus familiaris OX=9615 GN=ITIH4 PE=3 SV=1 | A0A8I3Q0B4 | *ITIH4* | 108 kDa | 0.28 | 0.552842 | 1.0604672 | 0.0847 |
| **204** | Actin-related protein 2/3 complex subunit 5 OS=Canis lupus familiaris OX=9615 GN=ARPC5 PE=3 SV=1 | A0A8I3MNR2 | *ARPC5* | 17 kDa | 0.28 | 0.552842 | 1.295940965 | 0.374 |
| **205** | Thrombospondin 4 OS=Canis lupus familiaris OX=9615 GN=THBS4 PE=3 SV=1 | A0A8I3N1V2 | *THBS4* | 104 kDa | 0.28 | 0.552842 | 2.828427125 | 1.5 |
| **206** | Proteasome 26S subunit, ATPase 2 OS=Canis lupus familiaris OX=9615 GN=PSMC2 PE=3 SV=1 | A0A8I3NXK9 | *PSMC2* | 46 kDa | 0.28 | 0.552842 | 3.706352248 | 1.89 |
| **207** | folate gamma-glutamyl hydrolase OS=Canis lupus familiaris OX=9615 GN=GGH PE=3 SV=1 | A0A8I3Q498 | *GGH* | 34 kDa | 0.29 | 0.537602 | 0.26425451 | -1.92 |
| **208** | Asparagine synthetase [glutamine-hydrolyzing] OS=Canis lupus familiaris OX=9615 GN=ASNS PE=4 SV=1 | A0A8I3PI35 | *ASNS* | 64 kDa | 0.29 | 0.537602 | 0.60583633 | -0.723 |
| **209** | Cellular communication network factor 3 OS=Canis lupus familiaris OX=9615 GN=CCN3 PE=3 SV=1 | A0A8I3MYU5 | *CCN3* | 42 kDa | 0.29 | 0.537602 | 0.998054152 | -0.00281 |
| **210** | Capping actin protein of muscle Z-line subunit alpha 2 OS=Canis lupus familiaris OX=9615 GN=CAPZA2 PE=3 SV=1 | A0A8I3N536 | *CAPZA2* | 44 kDa | 0.29 | 0.537602 | 1.0604672 | 0.0847 |
| **211** | Glypican 1 OS=Canis lupus familiaris OX=9615 GN=GPC1 PE=3 SV=1 | A0A8I3Q4U0 | *GPC1* | 61 kDa | 0.29 | 0.537602 | 1.339783602 | 0.422 |
| **212** | Malate dehydrogenase, mitochondrial OS=Canis lupus familiaris OX=9615 PE=3 SV=1 | A0A8I3PHB7 | | 29 kDa | 0.29 | 0.537602 | 1.484523571 | 0.57 |
| **213** | Twinfilin actin binding protein 1 OS=Canis lupus familiaris OX=9615 GN=TWF1 PE=3 SV=1 | A0A8I3QIT6 | *TWF1* | 40 kDa | 0.3 | 0.5228787 | 0.176776695 | -2.5 |
| **214** | ATP-dependent RNA helicase OS=Canis lupus familiaris OX=9615 GN=EIF4A2 PE=3 SV=1 | A0A8I3P8M4 | *EIF4A2* | 41 kDa | 0.3 | 0.5228787 | 0.647970483 | -0.626 |

| | | | | | | | | |
|---|---|---|---|---|---|---|---|---|
| **215** | Coatomer subunit delta OS=Canis lupus familiaris OX=9615 GN=ARCN1 PE=3 SV=1 | A0A8I3MM92 | *ARCN1* | 58 kDa | 0.3 | 0.5228787 | 0.67877249 | -0.559 |
| **216** | RRM domain-containing protein OS=Canis lupus familiaris OX=9615 GN=SFPQ PE=4 SV=1 | A0A8I3NVM0 | *SFPQ* | 72 kDa | 0.3 | 0.5228787 | 0.707106781 | -0.5 |
| **217** | Proteasome subunit alpha type OS=Canis lupus familiaris OX=9615 GN=PSMA5 PE=3 SV=1 | A0A8I3N3S4 | *PSMA5* | 26 kDa | 0.3 | 0.5228787 | 0.742261785 | -0.43 |
| **218** | CAP-Gly domain containing linker protein 1 OS=Canis lupus familiaris OX=9615 GN=CLIP1 PE=4 SV=1 | A0A8I3PIK9 | *CLIP1* | 152 kDa | 0.3 | 0.5228787 | 1.0604672 | 0.0847 |
| **219** | Major vault protein OS=Canis lupus familiaris OX=9615 GN=MVP PE=4 SV=1 | A0A8I3MGN1 | *MVP* | 99 kDa | 0.3 | 0.5228787 | 1.131314463 | 0.178 |
| **220** | Tropomyosin 1 OS=Canis lupus familiaris OX=9615 GN=TPM1 PE=3 SV=1 | A0A8I3PH06 | *TPM1* | 37 kDa | 0.3 | 0.5228787 | 1.16634937 | 0.222 |
| **221** | Nucleobindin 1 OS=Canis lupus familiaris OX=9615 GN=NUCB1 PE=3 SV=1 | A0A8I3RP80 | *NUCB1* | 53 kDa | 0.3 | 0.5228787 | 1.190856849 | 0.252 |
| **222** | Tenascin C OS=Canis lupus familiaris OX=9615 GN=TNC PE=3 SV=1 | A0A8I3NJZ1 | *TNC* | 250 kDa | 0.3 | 0.5228787 | 1.32592576 | 0.407 |
| **223** | Uncharacterized protein OS=Canis lupus familiaris OX=9615 GN=STIP1 PE=4 SV=1 | A0A8I3Q5C0 | *STIP1* | 68 kDa | 0.3 | 0.5228787 | 1.350037985 | 0.433 |
| **224** | Milk fat globule EGF and factor V/VIII domain containing OS=Canis lupus familiaris OX=9615 GN=MFGE8 PE=4 SV=1 | A0A8I3RT41 | *MFGE8* | 53 kDa | 0.3 | 0.5228787 | 1.350037985 | 0.433 |
| **225** | Septin 9 OS=Canis lupus familiaris OX=9615 GN=SEPTIN9 PE=3 SV=1 | A0A8I3NSH9 | *SEPTIN9* | 64 kDa | 0.3 | 0.5228787 | 1.591072968 | 0.67 |
| **226** | phenylalanine--tRNA ligase OS=Canis lupus familiaris OX=9615 GN=LOC119881735 PE=3 SV=1 | A0A8I3N3V5 | *LOC119881735* | 60 kDa | 0.31 | 0.5086383 | 0.303548721 | -1.72 |
| **227** | S-methyl-5'-thioadenosine phosphorylase OS=Canis lupus familiaris OX=9615 GN=MTAP PE=3 SV=1 | A0A8I3NWN6 | *MTAP* | 31 kDa | 0.31 | 0.5086383 | 0.397768242 | -1.33 |
| **228** | Aminopeptidase N OS=Canis lupus familiaris OX=9615 GN=ANPEP PE=1 SV=2 | P79143 | *ANPEP* | 110 kDa | 0.31 | 0.5086383 | 0.453759578 | -1.14 |
| **229** | VPS26, retromer complex component A OS=Canis lupus familiaris OX=9615 GN=VPS26A PE=3 SV=1 | A0A8I3N4R4 | *VPS26A* | 29 kDa | 0.31 | 0.5086383 | 0.530343871 | -0.915 |
| **230** | Integrin subunit beta like 1 OS=Canis lupus familiaris OX=9615 GN=ITGBL1 PE=4 SV=1 | A0A8I3Q988 | *ITGBL1* | 48 kDa | 0.31 | 0.5086383 | 0.848507902 | -0.237 |
| **231** | Canopy FGF signaling regulator 2 OS=Canis lupus familiaris OX=9615 GN=CNPY2 PE=3 SV=1 | A0A8I3RVQ5 | *CNPY2* | 21 kDa | 0.31 | 0.5086383 | 1.0604672 | 0.0847 |
| **232** | Protein arginine methyltransferase 8 OS=Canis lupus familiaris OX=9615 GN=PRMT8 PE=4 SV=1 | A0A8I3PPG1 | *PRMT8* | 45 kDa | 0.31 | 0.5086383 | 1.0604672 | 0.0847 |
| **233** | Glyceraldehyde-3-phosphate dehydrogenase OS=Canis lupus familiaris OX=9615 GN=LOC106558049 PE=3 SV=1 | A0A8I3Q6B9 | *LOC106558049* | 36 kDa | 0.31 | 0.5086383 | 1.156688184 | 0.21 |
| **234** | Inter-alpha-trypsin inhibitor heavy chain 2 OS=Canis lupus familiaris OX=9615 GN=ITIH2 PE=3 SV=1 | A0A8I3RRH5 | *ITIH2* | 107 kDa | 0.31 | 0.5086383 | 1.458009379 | 0.544 |
| **235** | Uncharacterized protein OS=Canis lupus familiaris OX=9615 GN=DDT PE=3 SV=1 | A0A8I3Q0N2 | *DDT* | 13 kDa | 0.31 | 0.5086383 | 1.666706414 | 0.737 |
| **236** | RAB2A, member RAS onco family OS=Canis lupus familiaris OX=9615 GN=RAB2A PE=4 SV=1 | A0A8I3Q185 | *RAB2A* | 21 kDa | 0.32 | 0.49485 | 0.530343871 | -0.915 |
| **237** | Complement C2 OS=Canis lupus familiaris OX=9615 GN=C2 PE=4 SV=1 | A0A8I3NF11 | *C2* | 86 kDa | 0.32 | 0.49485 | 0.581560021 | -0.782 |
| **238** | Retinitis pigmentosa GTPase regulator OS=Canis lupus familiaris OX=9615 GN=RPGR PE=4 SV=1 | A0A8I3PTH7 | *RPGR* | 49 kDa | 0.32 | 0.49485 | 0.623732786 | -0.681 |

| | | | | | | | | |
|---|---|---|---|---|---|---|---|---|
| **239** | T-complex protein 1 subunit delta OS=Canis lupus familiaris OX=9615 GN=CCT4 PE=3 SV=1 | A0A8I3NQN9 | *CCT4* | 55 kDa | 0.32 | 0.49485 | 0.643940815 | -0.635 |
| **240** | Synaptotagmin binding cytoplasmic RNA interacting protein OS=Canis lupus familiaris OX=9615 GN=SYNCRIP PE=4 SV=1 | A0A8I3NM71 | *SYNCRIP* | 63 kDa | 0.32 | 0.49485 | 0.66296288 | -0.593 |
| **241** | creatine kinase OS=Canis lupus familiaris OX=9615 GN=LOC100686502 PE=3 SV=1 | A0A8I3NY59 | *LOC100686502* | 35 kDa | 0.32 | 0.49485 | 0.669891801 | -0.578 |
| **242** | Transitional endoplasmic reticulum ATPase OS=Canis lupus familiaris OX=9615 GN=VCP PE=3 SV=1 | A0A8I3MWF5 | *VCP* | 89 kDa | 0.32 | 0.49485 | 0.922742493 | -0.116 |
| **243** | 40S ribosomal protein S6 OS=Canis lupus familiaris OX=9615 PE=3 SV=1 | A0A8I3PVU6 | | 29 kDa | 0.32 | 0.49485 | 0.94258851 | -0.0853 |
| **244** | Pregnancy zone protein-like OS=Canis lupus familiaris OX=9615 GN=LOC611458 PE=3 SV=1 | A0A8I3PX48 | *LOC611458* | 166 kDa | 0.32 | 0.49485 | 0.964063446 | -0.0528 |
| **245** | F-actin-capping protein subunit alpha OS=Canis lupus familiaris OX=9615 GN=CAPZA1 PE=3 SV=1 | A0A8I3NF50 | *CAPZA1* | 33 kDa | 0.32 | 0.49485 | 1.0604672 | 0.0847 |
| **246** | Drebrin 1 OS=Canis lupus familiaris OX=9615 GN=DBN1 PE=4 SV=1 | A0A8I3NNL1 | *DBN1* | 115 kDa | 0.32 | 0.49485 | 1.193335743 | 0.255 |
| **247** | Collagen type V alpha 1 chain OS=Canis lupus familiaris OX=9615 GN=COL5A1 PE=4 SV=1 | A0A8I3NPQ5 | *COL5A1* | 183 kDa | 0.32 | 0.49485 | 1.374588696 | 0.459 |
| **248** | CD44 antigen OS=Canis lupus familiaris OX=9615 GN=CD44 PE=4 SV=1 | A0A8I3PCF8 | *CD44* | 102 kDa | 0.32 | 0.49485 | 1.387030969 | 0.472 |
| **249** | Cathepsin K OS=Canis lupus familiaris OX=9615 GN=CTSK PE=2 SV=1 | Q3ZKN1 | *CTSK* | 37 kDa | 0.33 | 0.4814861 | 0.60583633 | -0.723 |
| **250** | Complement C1r OS=Canis lupus familiaris OX=9615 GN=C1R PE=4 SV=1 | A0A8I3PJQ7 | *C1R* | 89 kDa | 0.33 | 0.4814861 | 0.833353207 | -0.263 |
| **251** | Superoxide dismutase [Cu-Zn] OS=Canis lupus familiaris OX=9615 GN=SOD1 PE=3 SV=1 | A0A8I3PS52 | *SOD1* | 16 kDa | 0.33 | 0.4814861 | 0.897510051 | -0.156 |
| **252** | procollagen-proline 4-dioxygenase OS=Canis lupus familiaris OX=9615 GN=P4HA2 PE=3 SV=1 | A0A8I3N423 | *P4HA2* | 61 kDa | 0.33 | 0.4814861 | 1.0604672 | 0.0847 |
| **253** | RRM domain-containing protein OS=Canis lupus familiaris OX=9615 GN=LOC119866711 PE=4 SV=1 | A0A8I3PPR8 | *LOC119866711* | 42 kDa | 0.33 | 0.4814861 | 1.0604672 | 0.0847 |
| **254** | Sulfhydryl oxidase OS=Canis lupus familiaris OX=9615 GN=QSOX1 PE=3 SV=1 | A0A8I3MLE9 | *QSOX1* | 78 kDa | 0.33 | 0.4814861 | 1.216722359 | 0.283 |
| **255** | Actin-related protein 2/3 complex subunit 4 OS=Canis lupus familiaris OX=9615 GN=ARPC4 PE=3 SV=1 | A0A8I3N156 | *ARPC4* | 20 kDa | 0.33 | 0.4814861 | 1.378405153 | 0.463 |
| **256** | Ribosomal protein L10e/L16 domain-containing protein OS=Canis lupus familiaris OX=9615 GN=RPL10 PE=3 SV=1 | A0A8I3PXJ3 | *RPL10* | 25 kDa | 0.33 | 0.4814861 | 1.591072968 | 0.67 |
| **257** | WAP, follistatin/kazal, immunoglobulin, kunitz and netrin domain containing 2 OS=Canis lupus familiaris OX=9615 GN=WFIKKN2 PE=3 SV=1 | A0A8I3NJD8 | *WFIKKN2* | 63 kDa | 0.33 | 0.4814861 | 1.969732886 | 0.978 |
| **258** | Calreticulin OS=Canis lupus familiaris OX=9615 GN=CALR PE=3 SV=1 | A0A8I3NM03 | *CALR* | 48 kDa | 0.34 | 0.4685211 | 0.84264683 | -0.247 |
| **259** | Uncharacterized protein OS=Canis lupus familiaris OX=9615 GN=RPL5 PE=3 SV=1 | A0A8I3NV94 | *RPL5* | 34 kDa | 0.34 | 0.4685211 | 0.954422527 | -0.0673 |
| **260** | Aminopeptidase OS=Canis lupus familiaris OX=9615 GN=NPEPPS PE=3 SV=1 | A0A8I3RYM4 | *NPEPPS* | 97 kDa | 0.34 | 0.4685211 | 1.0604672 | 0.0847 |
| **261** | Proteasome subunit alpha type OS=Canis lupus familiaris OX=9615 GN=PSMA4 PE=3 SV=1 | A0A8I3NL81 | *PSMA4* | 20 kDa | 0.34 | 0.4685211 | 1.0604672 | 0.0847 |

| | | | | | | | | |
|---|---|---|---|---|---|---|---|---|
| **262** | Neudesin neurotrophic factor OS=Canis lupus familiaris OX=9615 GN=NENF PE=4 SV=1 | A0A8I3N6H0 | *NENF* | 19 kDa | 0.34 | 0.4685211 | 1.0604672 | 0.0847 |
| **263** | Cellular communication network factor 2 OS=Canis lupus familiaris OX=9615 GN=CCN2 PE=3 SV=1 | A0A8I3MXL6 | *CCN2* | 55 kDa | 0.34 | 0.4685211 | 1.372684431 | 0.457 |
| **264** | C-type lectin domain family 3 member B OS=Canis lupus familiaris OX=9615 GN=CLEC3B PE=4 SV=1 | A0A8I3NNJ6 | *CLEC3B* | 22 kDa | 0.34 | 0.4685211 | 1.387030969 | 0.472 |
| **265** | ATP-citrate synthase OS=Canis lupus familiaris OX=9615 GN=ACLY PE=3 SV=1 | A0A8I3NHY9 | *ACLY* | 121 kDa | 0.34 | 0.4685211 | 1.414213562 | 0.5 |
| **266** | Angiopoietin like 2 OS=Canis lupus familiaris OX=9615 GN=ANGPTL2 PE=4 SV=1 | A0A8I3N7R9 | *ANGPTL2* | 57 kDa | 0.35 | 0.455932 | 0.589269704 | -0.763 |
| **267** | G3BP stress granule assembly factor 1 OS=Canis lupus familiaris OX=9615 GN=G3BP1 PE=4 SV=1 | A0A8I3MXB3 | *G3BP1* | 52 kDa | 0.35 | 0.455932 | 0.623732786 | -0.681 |
| **268** | Large ribosomal subunit protein uL11 OS=Canis lupus familiaris OX=9615 PE=3 SV=1 | A0A8I3P848 | | 18 kDa | 0.35 | 0.455932 | 0.652477474 | -0.616 |
| **269** | S100/CaBP-9k-type calcium binding subdomain domain-containing protein OS=Canis lupus familiaris OX=9615 PE=4 SV=1 | A0A8I3PUW7 | | 28 kDa | 0.35 | 0.455932 | 0.908778116 | -0.138 |
| **270** | Proteasome subunit alpha type OS=Canis lupus familiaris OX=9615 GN=PSMA1 PE=3 SV=1 | A0A8I3PCT4 | *PSMA1* | 30 kDa | 0.35 | 0.455932 | 1.0604672 | 0.0847 |
| **271** | Peptidyl-prolyl cis-trans isomerase OS=Canis lupus familiaris OX=9615 PE=3 SV=1 | A0A8I3NVG7 | | 17 kDa | 0.35 | 0.455932 | 1.0604672 | 0.0847 |
| **272** | Large ribosomal subunit protein uL22 OS=Canis lupus familiaris OX=9615 PE=3 SV=1 | A0A8I3NL12 | | 14 kDa | 0.35 | 0.455932 | 1.0604672 | 0.0847 |
| **273** | Peptidyl-prolyl cis-trans isomerase OS=Canis lupus familiaris OX=9615 GN=PPIB PE=3 SV=1 | A0A8I3PCB7 | *PPIB* | 24 kDa | 0.35 | 0.455932 | 1.106497353 | 0.146 |
| **274** | Thioredoxin OS=Canis lupus familiaris OX=9615 GN=TXN PE=4 SV=1 | A0A8I3RXE2 | *TXN* | 10 kDa | 0.35 | 0.455932 | 1.125058485 | 0.17 |
| **275** | Coronin OS=Canis lupus familiaris OX=9615 GN=CORO1C PE=3 SV=1 | A0A8I3PST1 | *CORO1C* | 49 kDa | 0.35 | 0.455932 | 1.142346247 | 0.192 |
| **276** | Adenylate kinase isoenzyme 1 OS=Canis lupus familiaris OX=9615 GN=AK1 PE=3 SV=1 | B4YY02 | *AK1* | 23 kDa | 0.35 | 0.455932 | 1.21167266 | 0.277 |
| **277** | Fibronectin 1 OS=Canis lupus familiaris OX=9615 GN=FN1 PE=4 SV=1 | A0A8I3PLI0 | *FN1* | 250 kDa | 0.35 | 0.455932 | 1.30224419 | 0.381 |
| **278** | Endoplasmic reticulum resident protein 29 OS=Canis lupus familiaris OX=9615 GN=ERP29 PE=4 SV=1 | A0A8I3PAX2 | *ERP29* | 29 kDa | 0.36 | 0.4436975 | 0.60583633 | -0.723 |
| **279** | Cathepsin D OS=Canis lupus familiaris OX=9615 GN=CTSD PE=3 SV=1 | A0A8I3RVL8 | *CTSD* | 43 kDa | 0.36 | 0.4436975 | 0.707106781 | -0.5 |
| **280** | Small ribosomal subunit protein uS15 OS=Canis lupus familiaris OX=9615 GN=LOC480235 PE=3 SV=1 | A0A8I3Q153 | *LOC480235* | 17 kDa | 0.36 | 0.4436975 | 0.707106781 | -0.5 |
| **281** | Extended synaptotagmin 1 OS=Canis lupus familiaris OX=9615 GN=ESYT1 PE=3 SV=1 | A0A8I3N2N7 | *ESYT1* | 122 kDa | 0.36 | 0.4436975 | 0.707106781 | -0.5 |
| **282** | Procollagen-lysine,2-oxoglutarate 5-dioxygenase 3 OS=Canis lupus familiaris OX=9615 GN=PLOD3 PE=4 SV=1 | A0A8I3MUP6 | *PLOD3* | 84 kDa | 0.36 | 0.4436975 | 1.0604672 | 0.0847 |
| **283** | ATP-dependent 6-phosphofructokinase OS=Canis lupus familiaris OX=9615 GN=PFKP PE=3 SV=1 | A0A8I3MPH7 | *PFKP* | 86 kDa | 0.36 | 0.4436975 | 1.0604672 | 0.0847 |
| **284** | Clathrin light chain OS=Canis lupus familiaris OX=9615 GN=CLTA PE=3 SV=1 | A0A8I3NI30 | *CLTA* | 18 kDa | 0.36 | 0.4436975 | 1.193335743 | 0.255 |
| **285** | Desmin OS=Canis lupus familiaris OX=9615 GN=DES PE=3 SV=1 | A0A8I3Q5S1 | *DES* | 55 kDa | 0.36 | 0.4436975 | 1.292352831 | 0.37 |

| | | | | | | | | |
|---|---|---|---|---|---|---|---|---|
| **286** | 26S proteasome non-ATPase regulatory subunit 5 OS=Canis lupus familiaris OX=9615 GN=PSMD5 PE=3 SV=1 | A0A8I3NUK8 | *PSMD5* | 56 kDa | 0.36 | 0.4436975 | 1.591072968 | 0.67 |
| **287** | Amyloid beta precursor like protein 2 OS=Canis lupus familiaris OX=9615 GN=APLP2 PE=3 SV=1 | A0A8I3NFP2 | *APLP2* | 59 kDa | 0.37 | 0.4317983 | 0.423372656 | -1.24 |
| **288** | Ubiquitin carboxyl-terminal hydrolase OS=Canis lupus familiaris OX=9615 GN=USP5 PE=3 SV=1 | A0A8I3PWW2 | *USP5* | 96 kDa | 0.37 | 0.4317983 | 0.482968164 | -1.05 |
| **289** | procollagen-proline 4-dioxygenase OS=Canis lupus familiaris OX=9615 GN=P4HA1 PE=3 SV=1 | A0A8I3N1J1 | *P4HA1* | 61 kDa | 0.37 | 0.4317983 | 0.530343871 | -0.915 |
| **290** | Bleomycin hydrolase OS=Canis lupus familiaris OX=9615 GN=BLMH PE=3 SV=1 | A0A8I3NP01 | *BLMH* | 48 kDa | 0.37 | 0.4317983 | 0.530343871 | -0.915 |
| **291** | UV excision repair protein RAD23 OS=Canis lupus familiaris OX=9615 GN=RAD23A PE=3 SV=1 | A0A8I3NT37 | *RAD23A* | 40 kDa | 0.37 | 0.4317983 | 0.623732786 | -0.681 |
| **292** | SEC13 homolog, nuclear pore and COPII coat complex component OS=Canis lupus familiaris OX=9615 GN=SEC13 PE=3 SV=1 | A0A8I3RU56 | *SEC13* | 31 kDa | 0.37 | 0.4317983 | 0.636397468 | -0.652 |
| **293** | Metalloendopeptidase OS=Canis lupus familiaris OX=9615 GN=TLL2 PE=4 SV=1 | A0A8I3PS39 | *TLL2* | 114 kDa | 0.37 | 0.4317983 | 0.686342216 | -0.543 |
| **294** | Jupiter microtubule associated homolog 2 OS=Canis lupus familiaris OX=9615 GN=JPT2 PE=3 SV=1 | A0A8I3P624 | *JPT2* | 20 kDa | 0.37 | 0.4317983 | 0.707106781 | -0.5 |
| **295** | C1q and TNF related 3 OS=Canis lupus familiaris OX=9615 GN=C1QTNF3 PE=4 SV=1 | A0A8I3MD57 | *C1QTNF3* | 40 kDa | 0.37 | 0.4317983 | 0.867538687 | -0.205 |
| **296** | Histone H2B OS=Canis lupus familiaris OX=9615 GN=H2BC21 PE=3 SV=1 | A0A8I3NGL0 | *H2BC21* | 18 kDa | 0.37 | 0.4317983 | 1.142346247 | 0.192 |
| **297** | Collagen type II alpha 1 chain OS=Canis lupus familiaris OX=9615 GN=COL2A1 PE=4 SV=1 | A0A8I3PW42 | *COL2A1* | 134 kDa | 0.37 | 0.4317983 | 1.21167266 | 0.277 |
| **298** | Apolipoprotein A1 OS=Canis lupus familiaris OX=9615 GN=APOA1 PE=3 SV=1 | A0A8I3MSG4 | *APOA1* | 30 kDa | 0.37 | 0.4317983 | 1.295940965 | 0.374 |
| **299** | EGF containing fibulin extracellular matrix protein 1 OS=Canis lupus familiaris OX=9615 GN=EFEMP1 PE=4 SV=1 | A0A8I3NN55 | *EFEMP1* | 55 kDa | 0.37 | 0.4317983 | 1.659789171 | 0.731 |
| **300** | Large ribosomal subunit protein eL28 OS=Canis lupus familiaris OX=9615 GN=RPL28 PE=3 SV=1 | A0A8I3MCG8 | *RPL28* | 16 kDa | 0.37 | 0.4317983 | 2.657371628 | 1.41 |
| **301** | Mitogen-activated protein kinase OS=Canis lupus familiaris OX=9615 GN=MAPK1 PE=3 SV=1 | A0A8I3PZT6 | *MAPK1* | 36 kDa | 0.38 | 0.4202164 | 0.589269704 | -0.763 |
| **302** | AHNAK nucleoprotein OS=Canis lupus familiaris OX=9615 GN=AHNAK PE=4 SV=1 | A0A8I3NCQ4 | *AHNAK* | 657 kDa | 0.38 | 0.4202164 | 0.816203046 | -0.293 |
| **303** | Ezrin OS=Canis lupus familiaris OX=9615 GN=EZR PE=4 SV=1 | A0A8I3MKE1 | *EZR* | 69 kDa | 0.38 | 0.4202164 | 0.883927531 | -0.178 |
| **304** | non-specific serine/threonine protein kinase OS=Canis lupus familiaris OX=9615 GN=OXSR1 PE=4 SV=1 | A0A8I3S7C3 | *OXSR1* | 58 kDa | 0.38 | 0.4202164 | 1.0604672 | 0.0847 |
| **305** | Epidermal growth factor receptor pathway substrate 15 like 1 OS=Canis lupus familiaris OX=9615 GN=EPS15L1 PE=4 SV=1 | A0A8I3QMB9 | *EPS15L1* | 95 kDa | 0.38 | 0.4202164 | 1.0604672 | 0.0847 |
| **306** | NBL1, DAN family BMP antagonist OS=Canis lupus familiaris OX=9615 GN=NBL1 PE=3 SV=1 | A0A8I3RR78 | *NBL1* | 19 kDa | 0.38 | 0.4202164 | 1.484523571 | 0.57 |
| **307** | Splicing factor U2AF subunit OS=Canis lupus familiaris OX=9615 GN=U2AF2 PE=3 SV=1 | A0A8I3RP62 | *U2AF2* | 54 kDa | 0.39 | 0.4089354 | 0.530343871 | -0.915 |
| **308** | Coatomer subunit epsilon OS=Canis lupus familiaris OX=9615 GN=COPE PE=3 SV=1 | A0A8I3NZ27 | *COPE* | 35 kDa | 0.39 | 0.4089354 | 0.60583633 | -0.723 |

| | | | | | | | | |
|---|---|---|---|---|---|---|---|---|
| **309** | Uncharacterized protein OS=Canis lupus familiaris OX=9615 PE=4 SV=1 | A0A8I3S1Y0 | | 5 kDa | 0.39 | 0.4089354 | 0.707106781 | -0.5 |
| **310** | Uncharacterized protein OS=Canis lupus familiaris OX=9615 GN=TPT1 PE=3 SV=1 | A0A8I3N0W8 | *TPT1* | 20 kDa | 0.39 | 0.4089354 | 1.21167266 | 0.277 |
| **311** | Nestin OS=Canis lupus familiaris OX=9615 GN=NES PE=4 SV=1 | A0A8I3N5W6 | *NES* | 164 kDa | 0.39 | 0.4089354 | 1.855746953 | 0.892 |
| **312** | Alpha-1,4 glucan phosphorylase OS=Canis lupus familiaris OX=9615 GN=PYGB PE=3 SV=1 | A0A8I3Q6I7 | *PYGB* | 97 kDa | 0.39 | 0.4089354 | 2.657371628 | 1.41 |
| **313** | N-acetylglucosamine-6-sulfatase OS=Canis lupus familiaris OX=9615 GN=GNS PE=3 SV=1 | A0A8I3NER6 | *GNS* | 60 kDa | 0.4 | 0.39794 | 0.162667732 | -2.62 |
| **314** | Heat shock protein beta-1 OS=Canis lupus familiaris OX=9615 GN=Hsp27 PE=2 SV=1 | F1PYE3 | *Hsp27* | 23 kDa | 0.4 | 0.39794 | 0.636397468 | -0.652 |
| **315** | PCI domain-containing protein OS=Canis lupus familiaris OX=9615 PE=4 SV=1 | A0A8I3P8X2 | | 40 kDa | 0.4 | 0.39794 | 0.707106781 | -0.5 |
| **316** | Large ribosomal subunit protein eL30 OS=Canis lupus familiaris OX=9615 PE=3 SV=1 | A0A8I3MP22 | | 13 kDa | 0.4 | 0.39794 | 0.707106781 | -0.5 |
| **317** | Uncharacterized protein OS=Canis lupus familiaris OX=9615 PE=3 SV=1 | A0A8I3MH27 | | 51 kDa | 0.4 | 0.39794 | 0.93809114 | -0.0922 |
| **318** | Chloride intracellular channel protein OS=Canis lupus familiaris OX=9615 GN=CLIC4 PE=3 SV=1 | A0A8I3NSF2 | *CLIC4* | 28 kDa | 0.4 | 0.39794 | 1.126619228 | 0.172 |
| **319** | asparagine--tRNA ligase OS=Canis lupus familiaris OX=9615 GN=NARS1 PE=4 SV=1 | A0A8I3RRR8 | *NARS1* | 64 kDa | 0.4 | 0.39794 | 1.21167266 | 0.277 |
| **320** | Plasma retinol-binding protein OS=Canis lupus familiaris OX=9615 GN=RBP4 PE=3 SV=1 | A0A8I3S143 | *RBP4* | 39 kDa | 0.4 | 0.39794 | 1.21167266 | 0.277 |
| **321** | Reversion inducing cysteine rich protein with kazal motifs OS=Canis lupus familiaris OX=9615 GN=RECK PE=4 SV=1 | A0A8I3NTH3 | *RECK* | 17 kDa | 0.4 | 0.39794 | 1.414213562 | 0.5 |
| **322** | Laminin subunit beta 2 OS=Canis lupus familiaris OX=9615 GN=LAMB2 PE=4 SV=1 | A0A8I3QEM8 | *LAMB2* | 207 kDa | 0.4 | 0.39794 | 1.742308384 | 0.801 |
| **323** | Small ribosomal subunit protein uS10 OS=Canis lupus familiaris OX=9615 GN=LOC106559752 PE=3 SV=1 | A0A8I3RXY4 | *LOC106559752* | 11 kDa | 0.41 | 0.3872161 | 0.303548721 | -1.72 |
| **324** | Eukaryotic translation initiation factor 3 subunit J OS=Canis lupus familiaris OX=9615 GN=EIF3J PE=4 SV=1 | A0A8I3Q8I2 | *EIF3J* | 23 kDa | 0.41 | 0.3872161 | 0.530343871 | -0.915 |
| **325** | Talin 1 OS=Canis lupus familiaris OX=9615 GN=TLN1 PE=4 SV=1 | A0A8I3N6K0 | *TLN1* | 271 kDa | 0.41 | 0.3872161 | 0.919550046 | -0.121 |
| **326** | Plastin 3 OS=Canis lupus familiaris OX=9615 GN=PLS3 PE=4 SV=1 | A0A8I3NS61 | *PLS3* | 72 kDa | 0.41 | 0.3872161 | 0.923382311 | -0.115 |
| **327** | Tropomyosin 3 OS=Canis lupus familiaris OX=9615 GN=TPM3 PE=3 SV=1 | A0A8I3S1D8 | *TPM3* | 33 kDa | 0.41 | 0.3872161 | 0.927873476 | -0.108 |
| **328** | Nucleoside diphosphate kinase OS=Canis lupus familiaris OX=9615 GN=NME2 PE=3 SV=1 | A0A8I3NVY9 | *NME2* | 51 kDa | 0.41 | 0.3872161 | 0.927873476 | -0.108 |
| **329** | Leukotriene A(4) hydrolase OS=Canis lupus familiaris OX=9615 GN=LTA4H PE=3 SV=1 | A0A8I3PTW1 | *LTA4H* | 68 kDa | 0.41 | 0.3872161 | 0.94258851 | -0.0853 |
| **330** | RB binding protein 4, chromatin remodeling factor OS=Canis lupus familiaris OX=9615 GN=RBBP4 PE=4 SV=1 | A0A8I3N0Q4 | *RBBP4* | 48 kDa | 0.41 | 0.3872161 | 1.0604672 | 0.0847 |
| **331** | Lumican OS=Canis lupus familiaris OX=9615 GN=LUM PE=4 SV=1 | A0A8I3P684 | *LUM* | 38 kDa | 0.41 | 0.3872161 | 1.099616149 | 0.137 |
| **332** | Sushi repeat containing protein X-linked 2 OS=Canis lupus familiaris OX=9615 GN=SRPX2 PE=4 SV=1 | A0A8I3P3I8 | *SRPX2* | 52 kDa | 0.41 | 0.3872161 | 1.21167266 | 0.277 |

| | | | | | | | | |
|---|---|---|---|---|---|---|---|---|
| **333** | Zinc finger CCHC-type containing 13 OS=Canis lupus familiaris OX=9615 GN=ZCCHC13 PE=4 SV=1 | A0A8I3Q9V9 | *ZCCHC13* | 19 kDa | 0.41 | 0.3872161 | 1.767855062 | 0.822 |
| **334** | Palladin, cytoskeletal associated protein OS=Canis lupus familiaris OX=9615 GN=PALLD PE=4 SV=1 | A0A8I3PVG9 | *PALLD* | 146 kDa | 0.42 | 0.3767507 | 0.423372656 | -1.24 |
| **335** | Uncharacterized protein OS=Canis lupus familiaris OX=9615 GN=LRRFIP1 PE=3 SV=1 | A0A8I3Q6C3 | *LRRFIP1* | 106 kDa | 0.42 | 0.3767507 | 0.681601304 | -0.553 |
| **336** | SH3 domain binding glutamate rich protein like OS=Canis lupus familiaris OX=9615 GN=SH3BGRL PE=3 SV=1 | A0A8I3S5M4 | *SH3BGRL* | 15 kDa | 0.42 | 0.3767507 | 0.707106781 | -0.5 |
| **337** | Tropomyosin 1 OS=Canis lupus familiaris OX=9615 GN=TPM1 PE=3 SV=1 | A0A8I3P8C0 | *TPM1* | 45 kDa | 0.42 | 0.3767507 | 0.725476104 | -0.463 |
| **338** | ATP synthase F1 subunit epsilon OS=Canis lupus familiaris OX=9615 GN=ATP5IF1 PE=3 SV=1 | A0A8I3NGS0 | *ATP5IF1* | 12 kDa | 0.42 | 0.3767507 | 0.978877342 | -0.0308 |
| **339** | Cell division control protein 42 homolog OS=Canis lupus familiaris OX=9615 PE=3 SV=1 | A0A8I3P5T9 | | 22 kDa | 0.42 | 0.3767507 | 1.0604672 | 0.0847 |
| **340** | Collagen type I alpha 1 chain OS=Canis lupus familiaris OX=9615 GN=COL1A1 PE=4 SV=1 | A0A8I3RX37 | *COL1A1* | 149 kDa | 0.42 | 0.3767507 | 1.172834949 | 0.23 |
| **341** | Beta-2-microglobulin OS=Canis lupus familiaris OX=9615 PE=3 SV=1 | A0A8I3PXH7 | | 15 kDa | 0.42 | 0.3767507 | 1.219255094 | 0.286 |
| **342** | Plectin OS=Canis lupus familiaris OX=9615 GN=PLEC PE=4 SV=1 | A0A8I3MVC5 | *PLEC* | 388 kDa | 0.42 | 0.3767507 | 2.114036081 | 1.08 |
| **343** | High mobility group box 1 OS=Canis lupus familiaris OX=9615 GN=HMGB1 PE=3 SV=1 | A0A8I3P9Z9 | *HMGB1* | 23 kDa | 0.43 | 0.3665315 | 0.675018993 | -0.567 |
| **344** | GDP-L-fucose synthase OS=Canis lupus familiaris OX=9615 GN=GFUS PE=3 SV=1 | A0A8I3NTS0 | *GFUS* | 44 kDa | 0.43 | 0.3665315 | 0.707106781 | -0.5 |
| **345** | Mesencephalic astrocyte derived neurotrophic factor OS=Canis lupus familiaris OX=9615 GN=MANF PE=3 SV=1 | A0A8I3P5H2 | *MANF* | 21 kDa | 0.43 | 0.3665315 | 1.0604672 | 0.0847 |
| **346** | Actin gamma 1 OS=Canis lupus familiaris OX=9615 GN=ACTG1 PE=3 SV=1 | A0A8I3NE73 | *ACTG1* | 28 kDa | 0.43 | 0.3665315 | 1.101905116 | 0.14 |
| **347** | Protein S OS=Canis lupus familiaris OX=9615 GN=PROS1 PE=4 SV=1 | A0A8I3Q4J0 | *pro.01* | 67 kDa | 0.43 | 0.3665315 | 1.104964485 | 0.144 |
| **348** | Thyroglobulin type-1 domain-containing protein OS=Canis lupus familiaris OX=9615 GN=IGFBP6 PE=4 SV=1 | A0A8I3S4S1 | *IGFBP6* | 9 kDa | 0.43 | 0.3665315 | 1.161508732 | 0.216 |
| **349** | Dynein cytoplasmic 1 heavy chain 1 OS=Canis lupus familiaris OX=9615 GN=DYNC1H1 PE=3 SV=1 | A0A8I3RSP2 | *DYNC1H1* | 532 kDa | 0.44 | 0.3565473 | 0.283220971 | -1.82 |
| **350** | Cadherin 11 OS=Canis lupus familiaris OX=9615 GN=CDH11 PE=4 SV=1 | A0A8I3NLK4 | *CDH11* | 88 kDa | 0.44 | 0.3565473 | 0.411795509 | -1.28 |
| **351** | Asporin OS=Canis lupus familiaris OX=9615 GN=ASPN PE=3 SV=1 | A0A8I3PJ40 | *ASPN* | 42 kDa | 0.44 | 0.3565473 | 0.530343871 | -0.915 |
| **352** | Syntaxin 7 OS=Canis lupus familiaris OX=9615 GN=STX7 PE=3 SV=1 | A0A8I3MUA1 | *STX7* | 30 kDa | 0.44 | 0.3565473 | 0.795536484 | -0.33 |
| **353** | Large ribosomal subunit protein uL11 OS=Canis lupus familiaris OX=9615 GN=RPL12 PE=3 SV=1 | A0A8I3N1L0 | *RPL12* | 14 kDa | 0.44 | 0.3565473 | 0.795536484 | -0.33 |
| **354** | Syndecan 4 OS=Canis lupus familiaris OX=9615 GN=SDC4 PE=3 SV=1 | A0A8I3NWG4 | *SDC4* | 22 kDa | 0.44 | 0.3565473 | 0.848507902 | -0.237 |
| **355** | Tyrosine--tRNA ligase OS=Canis lupus familiaris OX=9615 GN=YARS1 PE=3 SV=1 | A0A8I3RU42 | *YARS1* | 56 kDa | 0.44 | 0.3565473 | 0.908778116 | -0.138 |
| **356** | Podocan OS=Canis lupus familiaris OX=9615 GN=PODN PE=4 SV=1 | A0A8I3P1R7 | *PODN* | 53 kDa | 0.44 | 0.3565473 | 0.94881584 | -0.0758 |

| | | | | | | | | |
|---|---|---|---|---|---|---|---|---|
| **357** | Decorin OS=Canis lupus familiaris OX=9615 GN=DCN PE=2 SV=2 | Q29393 | *DCN* | 40 kDa | 0.44 | 0.3565473 | 1.039651496 | 0.0561 |
| **358** | Antithrombin-III OS=Canis lupus familiaris OX=9615 GN=SERPINC1 PE=3 SV=1 | A0A8I3NHZ1 | *SERPINC1* | 61 kDa | 0.44 | 0.3565473 | 1.106497353 | 0.146 |
| **359** | HtrA serine peptidase 1 OS=Canis lupus familiaris OX=9615 GN=HTRA1 PE=3 SV=1 | A0A8I3PAG9 | *HTRA1* | 52 kDa | 0.44 | 0.3565473 | 1.125058485 | 0.17 |
| **360** | Spectrin beta chain OS=Canis lupus familiaris OX=9615 GN=SPTBN1 PE=3 SV=1 | A0A8I3N1N4 | *SPTBN1* | 275 kDa | 0.44 | 0.3565473 | 1.126619228 | 0.172 |
| **361** | peptidylprolyl isomerase OS=Canis lupus familiaris OX=9615 GN=FKBP10 PE=4 SV=1 | A0A8I3NDX1 | *FKBP10* | 64 kDa | 0.44 | 0.3565473 | 1.161508732 | 0.216 |
| **362** | Alpha-mannosidase OS=Canis lupus familiaris OX=9615 GN=MAN2B1 PE=3 SV=1 | A0A8I3NTM5 | *MAN2B1* | 110 kDa | 0.45 | 0.3467875 | 0.26425451 | -1.92 |
| **363** | Tryptophan--tRNA ligase, cytoplasmic OS=Canis lupus familiaris OX=9615 GN=WARS1 PE=3 SV=1 | A0A8I3RTG1 | *WARS1* | 55 kDa | 0.45 | 0.3467875 | 0.530343871 | -0.915 |
| **364** | cathepsin X OS=Canis lupus familiaris OX=9615 GN=CTSZ PE=3 SV=1 | A0A8I3NT46 | *CTSZ* | 44 kDa | 0.45 | 0.3467875 | 0.636397468 | -0.652 |
| **365** | Proteasome 20S subunit alpha 6 OS=Canis lupus familiaris OX=9615 GN=PSMA6 PE=3 SV=1 | A0A8I3MJ89 | *PSMA6* | 25 kDa | 0.45 | 0.3467875 | 0.681601304 | -0.553 |
| **366** | Microfibril associated protein 5 OS=Canis lupus familiaris OX=9615 GN=MFAP5 PE=3 SV=1 | A0A8I3Q7G6 | *MFAP5* | 21 kDa | 0.45 | 0.3467875 | 0.757333158 | -0.401 |
| **367** | 14-3-3 protein theta OS=Canis lupus familiaris OX=9615 GN=YWHAQ PE=3 SV=1 | A0A8I3PQU8 | *YWHAQ* | 28 kDa | 0.45 | 0.3467875 | 0.94258851 | -0.0853 |
| **368** | Eukaryotic translation initiation factor 5A OS=Canis lupus familiaris OX=9615 GN=EIF5A PE=3 SV=1 | A0A8I3PFB8 | *EIF5A* | 17 kDa | 0.45 | 0.3467875 | 0.954422527 | -0.0673 |
| **369** | Adenylyl cyclase-associated protein OS=Canis lupus familiaris OX=9615 GN=CAP2 PE=3 SV=1 | A0A8I3PQ45 | *CAP2* | 40 kDa | 0.45 | 0.3467875 | 0.964063446 | -0.0528 |
| **370** | Acidic leucine-rich nuclear phosphoprotein 32 family member OS=Canis lupus familiaris OX=9615 GN=ANP32B PE=3 SV=1 | A0A8I3MUS3 | *ANP32B* | 31 kDa | 0.45 | 0.3467875 | 1.0604672 | 0.0847 |
| **371** | Thioredoxin domain-containing protein OS=Canis lupus familiaris OX=9615 GN=TXNDC5 PE=4 SV=1 | A0A8I3S2V9 | *TXNDC5* | 48 kDa | 0.45 | 0.3467875 | 1.0604672 | 0.0847 |
| **372** | Fibronectin 1 OS=Canis lupus familiaris OX=9615 GN=FN1 PE=4 SV=1 | A0A8I3PHI4 | *FN1* | 263 kDa | 0.45 | 0.3467875 | 1.0604672 | 0.0847 |
| **373** | NAD(P)H quinone dehydrogenase 1 OS=Canis lupus familiaris OX=9615 GN=NQO1 PE=3 SV=1 | A0A8I3NME7 | *NQO1* | 26 kDa | 0.45 | 0.3467875 | 1.090507733 | 0.125 |
| **374** | Uncharacterized protein OS=Canis lupus familiaris OX=9615 PE=4 SV=1 | A0A8I3MY65 | | 87 kDa | 0.45 | 0.3467875 | 1.156688184 | 0.21 |
| **375** | Transgelin OS=Canis lupus familiaris OX=9615 GN=TAGLN2 PE=3 SV=1 | A0A8I3SCV9 | *TAGLN2* | 22 kDa | 0.45 | 0.3467875 | 1.203303026 | 0.267 |
| **376** | Serine/threonine-protein phosphatase OS=Canis lupus familiaris OX=9615 GN=PPP2CB PE=3 SV=1 | A0A8I3S1W8 | *PPP2CB* | 36 kDa | 0.45 | 0.3467875 | 1.414213562 | 0.5 |
| **377** | Thrombospondin 3 OS=Canis lupus familiaris OX=9615 GN=THBS3 PE=3 SV=1 | A0A8I3NMW4 | *THBS3* | 104 kDa | 0.45 | 0.3467875 | 1.414213562 | 0.5 |
| **378** | Quinoid dihydropteridine reductase OS=Canis lupus familiaris OX=9615 GN=QDPR PE=4 SV=1 | A0A8I3MG17 | *QDPR* | 22 kDa | 0.45 | 0.3467875 | 1.591072968 | 0.67 |
| **379** | 40S ribosomal protein S21 OS=Canis lupus familiaris OX=9615 GN=RPS21 PE=3 SV=1 | A0A8I3NWI3 | *RPS21* | 9 kDa | 0.46 | 0.3372422 | 0.453759578 | -1.14 |
| **380** | procollagen-lysine 5-dioxygenase OS=Canis lupus familiaris OX=9615 GN=PLOD2 PE=4 SV=1 | A0A8I3PE41 | *PLOD2* | 84 kDa | 0.46 | 0.3372422 | 0.553248677 | -0.854 |

| | | | | | | | | |
|---|---|---|---|---|---|---|---|---|
| **381** | 60 kDa heat shock protein, mitochondrial OS=Canis lupus familiaris OX=9615 GN=HSPD1 PE=3 SV=1 | A0A8I3Q1N0 | *HSPD1* | 59 kDa | 0.46 | 0.3372422 | 0.647970483 | -0.626 |
| **382** | Thioredoxin domain containing 17 OS=Canis lupus familiaris OX=9615 GN=TXNDC17 PE=4 SV=1 | A0A8I3MI79 | *TXNDC17* | 9 kDa | 0.46 | 0.3372422 | 0.707106781 | -0.5 |
| **383** | Hypoxia up-regulated 1 OS=Canis lupus familiaris OX=9615 GN=HYOU1 PE=3 SV=1 | A0A8I3MMG0 | *HYOU1* | 105 kDa | 0.46 | 0.3372422 | 0.883927531 | -0.178 |
| **384** | Histone H2B OS=Canis lupus familiaris OX=9615 PE=3 SV=1 | A0A8I3Q8U2 | | 12 kDa | 0.46 | 0.3372422 | 0.897510051 | -0.156 |
| **385** | phosphopyruvate hydratase OS=Canis lupus familiaris OX=9615 GN=ENO2 PE=3 SV=1 | A0A8I3SAA3 | *ENO2* | 43 kDa | 0.46 | 0.3372422 | 1.0604672 | 0.0847 |
| **386** | Small ubiquitin-related modifier OS=Canis lupus familiaris OX=9615 GN=SUMO2 PE=3 SV=1 | A0A8I3Q1K4 | *SUMO2* | 11 kDa | 0.46 | 0.3372422 | 1.0604672 | 0.0847 |
| **387** | Uncharacterized protein OS=Canis lupus familiaris OX=9615 GN=AIMP1 PE=4 SV=1 | A0A8I3Q1I7 | *AIMP1* | 35 kDa | 0.46 | 0.3372422 | 1.0604672 | 0.0847 |
| **388** | Bifunctional purine biosynthesis protein ATIC OS=Canis lupus familiaris OX=9615 GN=ATIC PE=3 SV=1 | A0A8I3PLK1 | *ATIC* | 65 kDa | 0.46 | 0.3372422 | 1.178539408 | 0.237 |
| **389** | Galectin-3-binding protein OS=Canis lupus familiaris OX=9615 GN=CANT1 PE=4 SV=1 | A0A8I3NTB5 | *CANT1* | 96 kDa | 0.46 | 0.3372422 | 1.219255094 | 0.286 |
| **390** | leucine--tRNA ligase OS=Canis lupus familiaris OX=9615 GN=LARS1 PE=3 SV=1 | A0A8I3PJ09 | *LARS1* | 131 kDa | 0.46 | 0.3372422 | 1.32592576 | 0.407 |
| **391** | Proteasome subunit beta OS=Canis lupus familiaris OX=9615 GN=PSMB2 PE=3 SV=1 | A0A8I3NUC6 | *PSMB2* | 23 kDa | 0.46 | 0.3372422 | 1.32592576 | 0.407 |
| **392** | Pre-mRNA-processing factor 19 OS=Canis lupus familiaris OX=9615 GN=PRPF19 PE=3 SV=1 | A0A8I3S308 | *PRPF19* | 55 kDa | 0.46 | 0.3372422 | 1.591072968 | 0.67 |
| **393** | Serpin family A member 7 OS=Canis lupus familiaris OX=9615 GN=SERPINA7 PE=3 SV=1 | A0A8I3NWV3 | *SERPINA7* | 49 kDa | 0.46 | 0.3372422 | 3.89061979 | 1.96 |
| **394** | Cytoskeleton associated protein 4 OS=Canis lupus familiaris OX=9615 GN=CKAP4 PE=4 SV=1 | A0A8I3NR35 | *CKAP4* | 65 kDa | 0.47 | 0.3279021 | 0.707106781 | -0.5 |
| **395** | Ribosomal protein L3 like OS=Canis lupus familiaris OX=9615 GN=RPL3L PE=3 SV=1 | A0A8I3PDF0 | *RPL3L* | 47 kDa | 0.47 | 0.3279021 | 0.707106781 | -0.5 |
| **396** | AP-2 complex subunit alpha OS=Canis lupus familiaris OX=9615 GN=AP2A1 PE=3 SV=1 | A0A8I3S2R3 | *AP2A1* | 105 kDa | 0.47 | 0.3279021 | 0.795536484 | -0.33 |
| **397** | Tropomyosin 1 OS=Canis lupus familiaris OX=9615 GN=TPM1 PE=3 SV=1 | A0A8I3PBQ8 | *TPM1* | 28 kDa | 0.47 | 0.3279021 | 0.857376037 | -0.222 |
| **398** | Uncharacterized protein OS=Canis lupus familiaris OX=9615 GN=UPP1 PE=3 SV=1 | A0A8I3RUZ7 | *UPP1* | 34 kDa | 0.47 | 0.3279021 | 0.883927531 | -0.178 |
| **399** | Small ribosomal subunit protein uS5 OS=Canis lupus familiaris OX=9615 GN=RPS2 PE=3 SV=1 | A0A8I3PAP3 | *RPS2* | 22 kDa | 0.47 | 0.3279021 | 0.998054152 | -0.00281 |
| **400** | calcium/calmodulin-dependent protein kinase OS=Canis lupus familiaris OX=9615 GN=CAMK2D PE=3 SV=1 | A0A8I3QH31 | *CAMK2D* | 59 kDa | 0.47 | 0.3279021 | 1.0604672 | 0.0847 |
| **401** | Glutathione S-transferase OS=Canis lupus familiaris OX=9615 GN=GSTP1 PE=3 SV=1 | A0A8I3N6E9 | *GSTP1* | 20 kDa | 0.47 | 0.3279021 | 1.161508732 | 0.216 |
| **402** | Tight junction protein 1 OS=Canis lupus familiaris OX=9615 GN=TJP1 PE=4 SV=1 | A0A8I3MFM5 | *TJP1* | 198 kDa | 0.47 | 0.3279021 | 2.114036081 | 1.08 |
| **403** | LDL receptor related protein 1 OS=Canis lupus familiaris OX=9615 GN=LRP1 PE=3 SV=1 | A0A8I3N9S8 | *LRP1* | 505 kDa | 0.48 | 0.3187588 | 0.325335464 | -1.62 |
| **404** | Eukaryotic translation initiation factor 2A OS=Canis lupus familiaris OX=9615 GN=EIF2A PE=3 SV=1 | A0A8I3PJX8 | *EIF2A* | 58 kDa | 0.48 | 0.3187588 | 0.353553391 | -1.5 |

| | | | | | | | | |
|---|---|---|---|---|---|---|---|---|
| **405** | Coatomer subunit gamma OS=Canis lupus familiaris OX=9615 GN=COPG1 PE=3 SV=1 | A0A8I3P8V4 | *COPG1* | 86 kDa | 0.48 | 0.3187588 | 0.530343871 | -0.915 |
| **406** | Annexin OS=Canis lupus familiaris OX=9615 GN=ANXA11 PE=3 SV=1 | A0A8I3P2S6 | *ANXA11* | 54 kDa | 0.48 | 0.3187588 | 0.636397468 | -0.652 |
| **407** | Heat shock protein 90 alpha family class A member 1 OS=Canis lupus familiaris OX=9615 GN=HSP90AA1 PE=3 SV=1 | A0A8I3MR78 | *HSP90AA1* | 85 kDa | 0.48 | 0.3187588 | 0.932386486 | -0.101 |
| **408** | IF rod domain-containing protein OS=Canis lupus familiaris OX=9615 PE=4 SV=1 | A0A8I3PND8 | | 47 kDa | 0.48 | 0.3187588 | 0.950000383 | -0.074 |
| **409** | RAB8A, member RAS onco family OS=Canis lupus familiaris OX=9615 GN=RAB8A PE=3 SV=1 | A0A8I3QBN7 | *RAB8A* | 30 kDa | 0.48 | 0.3187588 | 1.0604672 | 0.0847 |
| **410** | H1.2 linker histone, cluster member OS=Canis lupus familiaris OX=9615 GN=H1-2 PE=3 SV=1 | A0A8I3PV53 | *H1-2* | 21 kDa | 0.48 | 0.3187588 | 1.237132479 | 0.307 |
| **411** | Lamin B2 OS=Canis lupus familiaris OX=9615 GN=LMNB2 PE=3 SV=1 | A0A8I3NGI5 | *LMNB2* | 76 kDa | 0.48 | 0.3187588 | 1.25353302 | 0.326 |
| **412** | Apolipoprotein E OS=Canis lupus familiaris OX=9615 GN=APOE PE=1 SV=3 | P18649 | *APOE* | 37 kDa | 0.48 | 0.3187588 | 1.330529041 | 0.412 |
| **413** | Uncharacterized protein OS=Canis lupus familiaris OX=9615 GN=LOC491316 PE=4 SV=1 | A0A8I3Q1D2 | *LOC491316* | 15 kDa | 0.49 | 0.3098039 | 0.530343871 | -0.915 |
| **414** | Phosphatidylinositol transfer protein beta OS=Canis lupus familiaris OX=9615 GN=PITPNB PE=4 SV=1 | A0A8I3Q8X6 | *PITPNB* | 32 kDa | 0.49 | 0.3098039 | 0.742261785 | -0.43 |
| **415** | Tissue factor pathway inhibitor OS=Canis lupus familiaris OX=9615 GN=TFPI2 PE=4 SV=1 | A0A8I3NZH5 | *TFPI2* | 26 kDa | 0.49 | 0.3098039 | 1.441928871 | 0.528 |
| **416** | Aconitase 1 OS=Canis lupus familiaris OX=9615 GN=ACO1 PE=3 SV=1 | A0A8I3P9E5 | *ACO1* | 100 kDa | 0.49 | 0.3098039 | 1.591072968 | 0.67 |
| **417** | Laminin subunit alpha 2 OS=Canis lupus familiaris OX=9615 GN=LAMA2 PE=4 SV=1 | A0A8I3RRA4 | *LAMA2* | 341 kDa | 0.5 | 0.30103 | 0.301451957 | -1.73 |
| **418** | H1.4 linker histone, cluster member OS=Canis lupus familiaris OX=9615 GN=H1-4 PE=3 SV=1 | A0A8I3PW81 | *H1-4* | 22 kDa | 0.5 | 0.30103 | 0.530343871 | -0.915 |
| **419** | Prosaposin OS=Canis lupus familiaris OX=9615 GN=PSAP PE=4 SV=1 | A0A8I3MQM8 | *PSAP* | 58 kDa | 0.5 | 0.30103 | 0.775930854 | -0.366 |
| **420** | Metalloendopeptidase OS=Canis lupus familiaris OX=9615 GN=BMP1 PE=4 SV=1 | A0A8I3PQL4 | *BMP1* | 112 kDa | 0.5 | 0.30103 | 1.0604672 | 0.0847 |
| **421** | Uncharacterized protein OS=Canis lupus familiaris OX=9615 GN=SERPINE2 PE=3 SV=1 | A0A8I3NVU4 | *SERPINE2* | 46 kDa | 0.5 | 0.30103 | 1.126619228 | 0.172 |
| **422** | Uncharacterized protein OS=Canis lupus familiaris OX=9615 GN=RPL24 PE=3 SV=1 | A0A8I3PBR3 | *RPL24* | 18 kDa | 0.51 | 0.2924298 | 0.636397468 | -0.652 |
| **423** | Proteasome subunit beta OS=Canis lupus familiaris OX=9615 GN=PSMB5 PE=3 SV=1 | A0A8I3MNI0 | *PSMB5* | 29 kDa | 0.51 | 0.2924298 | 0.652477474 | -0.616 |
| **424** | Proteasome subunit beta OS=Canis lupus familiaris OX=9615 GN=PSMB1 PE=3 SV=1 | A0A8I3PKH4 | *PSMB1* | 26 kDa | 0.51 | 0.2924298 | 0.681601304 | -0.553 |
| **425** | Elastin OS=Canis lupus familiaris OX=9615 GN=ELN PE=3 SV=1 | A0A8I3MRF8 | *ELN* | 65 kDa | 0.51 | 0.2924298 | 0.78132788 | -0.356 |
| **426** | Protein disulfide-isomerase A6 OS=Canis lupus familiaris OX=9615 GN=PDIA6 PE=3 SV=1 | A0A8I3SAL0 | *PDIA6* | 48 kDa | 0.51 | 0.2924298 | 0.877821798 | -0.188 |
| **427** | Caldesmon 1 OS=Canis lupus familiaris OX=9615 GN=CALD1 PE=4 SV=1 | A0A8I3N4C3 | *CALD1* | 63 kDa | 0.51 | 0.2924298 | 1.119612889 | 0.163 |
| **428** | Collagen type I alpha 2 chain OS=Canis lupus familiaris OX=9615 GN=COL1A2 PE=4 SV=1 | A0A8I3P1L4 | *COL1A2* | 120 kDa | 0.51 | 0.2924298 | 1.134455485 | 0.182 |

| | | | | | | | | |
|---|---|---|---|---|---|---|---|---|
| **429** | Collagen type IV alpha 2 chain OS=Canis lupus familiaris OX=9615 GN=COL4A2 PE=4 SV=1 | A0A8I3PJI8 | *COL4A2* | 203 kDa | 0.51 | 0.2924298 | 1.401527449 | 0.487 |
| **430** | Clathrin light chain OS=Canis lupus familiaris OX=9615 GN=CLTB PE=3 SV=1 | A0A8I3NML2 | *CLTB* | 23 kDa | 0.52 | 0.2839967 | 0.530343871 | -0.915 |
| **431** | Elastin microfibril interfacer 2 OS=Canis lupus familiaris OX=9615 GN=EMILIN2 PE=4 SV=1 | A0A8I3MQ82 | *EMILIN2* | 111 kDa | 0.52 | 0.2839967 | 0.667574152 | -0.583 |
| **432** | Myoferlin OS=Canis lupus familiaris OX=9615 GN=MYOF PE=3 SV=1 | A0A8I3PC83 | *MYOF* | 235 kDa | 0.52 | 0.2839967 | 0.707106781 | -0.5 |
| **433** | Rab GDP dissociation inhibitor OS=Canis lupus familiaris OX=9615 GN=GDI1 PE=3 SV=1 | A0A8I3Q509 | *GDI1* | 51 kDa | 0.52 | 0.2839967 | 0.762072415 | -0.392 |
| **434** | Clathrin light chain OS=Canis lupus familiaris OX=9615 GN=CLTA PE=3 SV=1 | A0A8I3NEF5 | *CLTA* | 25 kDa | 0.52 | 0.2839967 | 1.0604672 | 0.0847 |
| **435** | Dihydropyrimidinase like 3 OS=Canis lupus familiaris OX=9615 GN=DPYSL3 PE=3 SV=1 | A0A8I3PFJ6 | *DPYSL3* | 75 kDa | 0.52 | 0.2839967 | 1.136029265 | 0.184 |
| **436** | Collagen type IV alpha 1 chain OS=Canis lupus familiaris OX=9615 GN=COL4A1 PE=4 SV=1 | A0A8I3S0U4 | *COL4A1* | 160 kDa | 0.52 | 0.2839967 | 1.42899414 | 0.515 |
| **437** | Lysyl oxidase homolog OS=Canis lupus familiaris OX=9615 GN=LOX PE=3 SV=1 | A0A8I3N9H6 | *LOX* | 46 kDa | 0.52 | 0.2839967 | 1.45296505 | 0.539 |
| **438** | Ribosomal protein S5 C-terminal domain-containing protein OS=Canis lupus familiaris OX=9615 PE=4 SV=1 | A0A8I3NNL0 | | 9 kDa | 0.53 | 0.2757241 | 0.60583633 | -0.723 |
| **439** | PWWP domain-containing protein OS=Canis lupus familiaris OX=9615 GN=HDGF PE=4 SV=1 | A0A8I3RUF1 | *HDGF* | 27 kDa | 0.53 | 0.2757241 | 0.613867842 | -0.704 |
| **440** | Chaperonin containing TCP1 subunit 5 OS=Canis lupus familiaris OX=9615 GN=CCT5 PE=3 SV=1 | A0A8I3PI04 | *CCT5* | 54 kDa | 0.53 | 0.2757241 | 0.679714121 | -0.557 |
| **441** | Creatine kinase B-type OS=Canis lupus familiaris OX=9615 GN=CKB PE=1 SV=1 | P05124 | *CKB* | 43 kDa | 0.53 | 0.2757241 | 0.801069878 | -0.32 |
| **442** | PDZ and LIM domain 1 OS=Canis lupus familiaris OX=9615 GN=PDLIM1 PE=4 SV=1 | A0A8I3S614 | *PDLIM1* | 36 kDa | 0.53 | 0.2757241 | 0.824733549 | -0.278 |
| **443** | L-lactate dehydrogenase OS=Canis lupus familiaris OX=9615 GN=LDHB PE=3 SV=1 | A0A8I3NUJ8 | *LDHB* | 36 kDa | 0.53 | 0.2757241 | 0.883927531 | -0.178 |
| **444** | Calpain-2 catalytic subunit OS=Canis lupus familiaris OX=9615 GN=CAPN2 PE=3 SV=1 | A0A8I3NDL3 | *CAPN2* | 80 kDa | 0.53 | 0.2757241 | 0.966874134 | -0.0486 |
| **445** | Small ribosomal subunit protein uS13 OS=Canis lupus familiaris OX=9615 GN=RPS18 PE=3 SV=1 | A0A8I3PLA9 | *RPS18* | 19 kDa | 0.53 | 0.2757241 | 1.0604672 | 0.0847 |
| **446** | Myosin heavy chain 14 OS=Canis lupus familiaris OX=9615 GN=MYH14 PE=3 SV=1 | A0A8I3P8R0 | *MYH14* | 232 kDa | 0.53 | 0.2757241 | 1.173648178 | 0.231 |
| **447** | 45 kDa calcium-binding protein OS=Canis lupus familiaris OX=9615 GN=SDF4 PE=3 SV=1 | A0A8I3PU05 | *SDF4* | 41 kDa | 0.53 | 0.2757241 | 1.817556233 | 0.862 |
| **448** | Proteasome subunit beta OS=Canis lupus familiaris OX=9615 GN=PSMB4 PE=3 SV=1 | A0A8I3PII7 | *PSMB4* | 29 kDa | 0.54 | 0.2676062 | 0.423372656 | -1.24 |
| **449** | Heterogeneous nuclear ribonucleoprotein U like 2 OS=Canis lupus familiaris OX=9615 GN=HNRNPUL2 PE=4 SV=1 | A0A8I3MZ83 | *HNRNPUL2* | 81 kDa | 0.54 | 0.2676062 | 0.530343871 | -0.915 |
| **450** | Peroxidasin OS=Canis lupus familiaris OX=9615 GN=PXDN PE=4 SV=1 | A0A8I3P3Y3 | *PXDN* | 164 kDa | 0.54 | 0.2676062 | 0.649769531 | -0.622 |
| **451** | ceramidase OS=Canis lupus familiaris OX=9615 GN=ASAH1 PE=3 SV=1 | A0A8I3S2J6 | *ASAH1* | 41 kDa | 0.54 | 0.2676062 | 0.734075318 | -0.446 |
| **452** | Nuclear casein kinase and cyclin dependent kinase substrate 1 OS=Canis lupus familiaris OX=9615 GN=NUCKS1 PE=4 SV=1 | A0A8I3PTX6 | *NUCKS1* | 28 kDa | 0.54 | 0.2676062 | 0.757333158 | -0.401 |

| | | | | | | | | |
|---|---|---|---|---|---|---|---|---|
| **453** | Integral membrane protein 2 OS=Canis lupus familiaris OX=9615 GN=ITM2B PE=3 SV=1 | A0A8I3N344 | *ITM2B* | 18 kDa | 0.54 | 0.2676062 | 1.0604672 | 0.0847 |
| **454** | Uncharacterized protein OS=Canis lupus familiaris OX=9615 GN=CFD PE=4 SV=1 | A0A8I3NIC8 | *CFD* | 44 kDa | 0.54 | 0.2676062 | 1.131314463 | 0.178 |
| **455** | Collagen type V alpha 2 chain OS=Canis lupus familiaris OX=9615 GN=COL5A2 PE=4 SV=1 | A0A8I3PBM1 | *COL5A2* | 141 kDa | 0.54 | 0.2676062 | 1.144724161 | 0.195 |
| **456** | Insulin like growth factor binding protein 7 OS=Canis lupus familiaris OX=9615 GN=IGFBP7 PE=4 SV=1 | A0A8I3P1V2 | *IGFBP7* | 31 kDa | 0.54 | 0.2676062 | 1.255271991 | 0.328 |
| **457** | Ubiquilin 1 OS=Canis lupus familiaris OX=9615 GN=UBQLN1 PE=4 SV=1 | A0A8I3MWP5 | *UBQLN1* | 62 kDa | 0.54 | 0.2676062 | 2.114036081 | 1.08 |
| **458** | Inhibin beta A chain OS=Canis lupus familiaris OX=9615 GN=INHBA PE=3 SV=1 | A0A8I3P2Y0 | *INHBA* | 47 kDa | 0.54 | 0.2676062 | 2.265767771 | 1.18 |
| **459** | SEC31 homolog A, COPII coat complex component OS=Canis lupus familiaris OX=9615 GN=SEC31A PE=3 SV=1 | A0A8I3PIX0 | *SEC31A* | 129 kDa | 0.55 | 0.2596373 | 0.463294031 | -1.11 |
| **460** | Uncharacterized protein OS=Canis lupus familiaris OX=9615 GN=UBE2L3 PE=3 SV=1 | A0A8I3PWH6 | *UBE2L3* | 14 kDa | 0.55 | 0.2596373 | 0.848507902 | -0.237 |
| **461** | Uncharacterized protein OS=Canis lupus familiaris OX=9615 GN=PSME1 PE=3 SV=1 | A0A8I3MU16 | *PSME1* | 29 kDa | 0.55 | 0.2596373 | 0.978877342 | -0.0308 |
| **462** | Large ribosomal subunit protein eL14 OS=Canis lupus familiaris OX=9615 GN=RPL14 PE=3 SV=1 | A0A8I3RZN5 | *RPL14* | 23 kDa | 0.55 | 0.2596373 | 1.0604672 | 0.0847 |
| **463** | Phenylalanine--tRNA ligase alpha subunit OS=Canis lupus familiaris OX=9615 GN=FARSA PE=3 SV=1 | A0A8I3NX27 | *FARSA* | 57 kDa | 0.55 | 0.2596373 | 1.0604672 | 0.0847 |
| **464** | Nucleobindin 2 OS=Canis lupus familiaris OX=9615 GN=NUCB2 PE=3 SV=1 | A0A8I3NSK9 | *NUCB2* | 50 kDa | 0.55 | 0.2596373 | 1.148698355 | 0.2 |
| **465** | Laminin subunit alpha 4 OS=Canis lupus familiaris OX=9615 GN=LAMA4 PE=4 SV=1 | A0A8I3PBN7 | *LAMA4* | 184 kDa | 0.55 | 0.2596373 | 1.185092771 | 0.245 |
| **466** | Thrombospondin 1 OS=Canis lupus familiaris OX=9615 GN=THBS1 PE=3 SV=1 | A0A8I3PPU8 | *THBS1* | 159 kDa | 0.55 | 0.2596373 | 1.435944511 | 0.522 |
| **467** | Coiled-coil domain containing 80 OS=Canis lupus familiaris OX=9615 GN=CCDC80 PE=4 SV=1 | A0A8I3Q3Q8 | *CCDC80* | 107 kDa | 0.55 | 0.2596373 | 1.445932295 | 0.532 |
| **468** | Extracellular matrix protein 1 OS=Canis lupus familiaris OX=9615 GN=ECM1 PE=4 SV=1 | A0A8I3P5P0 | *ECM1* | 65 kDa | 0.55 | 0.2596373 | 1.455989549 | 0.542 |
| **469** | Uncharacterized protein OS=Canis lupus familiaris OX=9615 GN=PAPSS2 PE=3 SV=1 | A0A8I3Q7N2 | *PAPSS2* | 70 kDa | 0.56 | 0.251812 | 0.530343871 | -0.915 |
| **470** | 40S ribosomal protein S4 OS=Canis lupus familiaris OX=9615 GN=LOC102154781 PE=3 SV=1 | A0A8I3MMM1 | *LOC102154781* | 29 kDa | 0.56 | 0.251812 | 0.60583633 | -0.723 |
| **471** | UTP--glucose-1-phosphate uridylyltransferase OS=Canis lupus familiaris OX=9615 GN=UGP2 PE=3 SV=1 | A0A8I3S420 | *UGP2* | 57 kDa | 0.56 | 0.251812 | 0.669891801 | -0.578 |
| **472** | Actin-related protein 2/3 complex subunit OS=Canis lupus familiaris OX=9615 GN=ARPC1A PE=3 SV=1 | A0A8I3MPK0 | *ARPC1A* | 42 kDa | 0.56 | 0.251812 | 0.707106781 | -0.5 |
| **473** | 60S ribosomal protein L6 OS=Canis lupus familiaris OX=9615 GN=RPL6 PE=3 SV=1 | F1Q424 | *RPL6* | 33 kDa | 0.56 | 0.251812 | 0.746389192 | -0.422 |
| **474** | 60S ribosomal protein L10a OS=Canis lupus familiaris OX=9615 PE=4 SV=1 | A0A8I3NJD1 | | 39 kDa | 0.56 | 0.251812 | 0.771105413 | -0.375 |
| **475** | Dynein cytoplasmic 1 intermediate chain 2 OS=Canis lupus familiaris OX=9615 GN=DYNC1I2 PE=3 SV=1 | A0A8I3PYE3 | *DYNC1I2* | 71 kDa | 0.56 | 0.251812 | 0.883927531 | -0.178 |
| **476** | 6-phosphogluconate dehydrogenase, decarboxylating OS=Canis lupus familiaris OX=9615 GN=PGD PE=3 SV=1 | A0A8I3MQ54 | *PGD* | 53 kDa | 0.56 | 0.251812 | 1.0604672 | 0.0847 |

| | | | | | | | | |
|---|---|---|---|---|---|---|---|---|
| **477** | Glucose-6-phosphate isomerase OS=Canis lupus familiaris OX=9615 PE=3 SV=1 | A0A8I3RR36 | | 61 kDa | 0.56 | 0.251812 | 1.098092814 | 0.135 |
| **478** | phosphopyruvate hydratase OS=Canis lupus familiaris OX=9615 GN=ENO1 PE=3 SV=1 | A0A8I3PVT9 | *ENO1* | 47 kDa | 0.56 | 0.251812 | 1.139183377 | 0.188 |
| **479** | Latent transforming growth factor beta binding protein 1 OS=Canis lupus familiaris OX=9615 GN=LTBP1 PE=4 SV=1 | A0A8I3NFL4 | *LTBP1* | 180 kDa | 0.56 | 0.251812 | 1.257884972 | 0.331 |
| **480** | Heterogeneous nuclear ribonucleoprotein R OS=Canis lupus familiaris OX=9615 GN=HNRNPR PE=4 SV=1 | A0A8I3P1I8 | *HNRNPR* | 56 kDa | 0.56 | 0.251812 | 1.363202607 | 0.447 |
| **481** | Nidogen 2 OS=Canis lupus familiaris OX=9615 GN=NID2 PE=4 SV=1 | A0A8I3MQB2 | *NID2* | 160 kDa | 0.56 | 0.251812 | 1.514666316 | 0.599 |
| **482** | peptidylprolyl isomerase OS=Canis lupus familiaris OX=9615 GN=LOC607841 PE=4 SV=1 | A0A8I3NBC7 | *LOC607841* | 25 kDa | 0.57 | 0.2441251 | 0.771105413 | -0.375 |
| **483** | Peroxiredoxin 2 OS=Canis lupus familiaris OX=9615 GN=PRDX2 PE=4 SV=1 | A0A8I3NXZ9 | *PRDX2* | 15 kDa | 0.58 | 0.236572 | 0.66296288 | -0.593 |
| **484** | Small ribosomal subunit protein uS5 OS=Canis lupus familiaris OX=9615 PE=4 SV=1 | A0A8I3PFW4 | | 25 kDa | 0.58 | 0.236572 | 0.707106781 | -0.5 |
| **485** | RRM domain-containing protein OS=Canis lupus familiaris OX=9615 GN=HNRNPC PE=3 SV=1 | A0A8I3PNS7 | *HNRNPC* | 33 kDa | 0.58 | 0.236572 | 0.722966147 | -0.468 |
| **486** | Collagen type I alpha 2 chain OS=Canis lupus familiaris OX=9615 GN=COL1A2 PE=4 SV=1 | A0A8I3P828 | *COL1A2* | 129 kDa | 0.58 | 0.236572 | 0.774855931 | -0.368 |
| **487** | Dihydropyrimidinase like 3 OS=Canis lupus familiaris OX=9615 GN=DPYSL3 PE=3 SV=1 | A0A8I3PFZ1 | *DPYSL3* | 62 kDa | 0.58 | 0.236572 | 0.815637493 | -0.294 |
| **488** | E1 ubiquitin-activating enzyme OS=Canis lupus familiaris OX=9615 GN=UBA1 PE=3 SV=1 | A0A8I3Q8F0 | *UBA1* | 118 kDa | 0.58 | 0.236572 | 0.821880187 | -0.283 |
| **489** | Fascin OS=Canis lupus familiaris OX=9615 GN=FSCN1 PE=3 SV=1 | A0A8I3S028 | *FSCN1* | 53 kDa | 0.58 | 0.236572 | 0.988148656 | -0.0172 |
| **490** | Lysyl oxidase homolog OS=Canis lupus familiaris OX=9615 GN=LOXL1 PE=3 SV=1 | A0A8I3S6M8 | *LOXL1* | 75 kDa | 0.58 | 0.236572 | 0.989725257 | -0.0149 |
| **491** | Fibulin-1 OS=Canis lupus familiaris OX=9615 GN=FBLN1 PE=3 SV=1 | A0A8I3NSN5 | *FBLN1* | 75 kDa | 0.58 | 0.236572 | 1.074749173 | 0.104 |
| **492** | Insulin-like growth factor-binding protein 4 OS=Canis lupus familiaris OX=9615 GN=IGFBP4 PE=4 SV=1 | A0A8I3NA54 | *IGFBP4* | 28 kDa | 0.58 | 0.236572 | 1.187559666 | 0.248 |
| **493** | Actin, cytoplasmic 1 OS=Canis lupus familiaris OX=9615 GN=ACTB PE=2 SV=3 | O18840 | *ACTB* | 42 kDa | 0.58 | 0.236572 | 1.217566019 | 0.284 |
| **494** | High mobility group protein HMG-I/HMG-Y OS=Canis lupus familiaris OX=9615 GN=HMGA1 PE=3 SV=3 | Q6URC2 | *HMGA1* | 12 kDa | 0.58 | 0.236572 | 1.32592576 | 0.407 |
| **495** | FAM3 metabolism regulating signaling molecule C OS=Canis lupus familiaris OX=9615 GN=FAM3C PE=3 SV=1 | A0A8I3N7J4 | *FAM3C* | 25 kDa | 0.58 | 0.236572 | 1.591072968 | 0.67 |
| **496** | Eukaryotic translation initiation factor 4B OS=Canis lupus familiaris OX=9615 GN=EIF4B PE=4 SV=1 | A0A8I3S3N6 | *EIF4B* | 70 kDa | 0.59 | 0.229148 | 0.530343871 | -0.915 |
| **497** | Isocitrate dehydrogenase [NADP] OS=Canis lupus familiaris OX=9615 GN=IDH1 PE=2 SV=1 | F1PZA1 | *IDH1* | 47 kDa | 0.59 | 0.229148 | 0.636397468 | -0.652 |
| **498** | Protein arginine methyltransferase 1 OS=Canis lupus familiaris OX=9615 GN=PRMT1 PE=4 SV=1 | A0A8I3PS34 | *PRMT1* | 42 kDa | 0.59 | 0.229148 | 0.636397468 | -0.652 |
| **499** | Polyadenylate-binding protein OS=Canis lupus familiaris OX=9615 GN=PABPC1 PE=3 SV=1 | A0A8I3PXD4 | *PABPC1* | 67 kDa | 0.59 | 0.229148 | 0.675018993 | -0.567 |
| **500** | ATP binding cassette subfamily F member 1 OS=Canis lupus familiaris OX=9615 GN=ABCF1 PE=4 SV=1 | A0A8I3N7G8 | *ABCF1* | 96 kDa | 0.59 | 0.229148 | 0.707106781 | -0.5 |

| | | | | | | | | |
|---|---|---|---|---|---|---|---|---|
| **501** | Sulfhydryl oxidase OS=Canis lupus familiaris OX=9615 GN=QSOX1 PE=3 SV=1 | A0A8I3RQL9 | *QSOX1* | 82 kDa | 0.59 | 0.229148 | 0.771105413 | -0.375 |
| **502** | Kinectin 1 OS=Canis lupus familiaris OX=9615 GN=KTN1 PE=4 SV=1 | A0A8I3P295 | *KTN1* | 69 kDa | 0.59 | 0.229148 | 0.824733549 | -0.278 |
| **503** | Heat shock protein family B (small) member 1 OS=Canis lupus familiaris OX=9615 GN=HSPB1 PE=3 SV=1 | A0A8I3MMU2 | *HSPB1* | 18 kDa | 0.59 | 0.229148 | 0.837406488 | -0.256 |
| **504** | Myosin heavy chain 10 OS=Canis lupus familiaris OX=9615 GN=MYH10 PE=3 SV=1 | A0A8I3MIQ6 | *MYH10* | 232 kDa | 0.59 | 0.229148 | 0.935688353 | -0.0959 |
| **505** | Parkinsonism associated deglycase OS=Canis lupus familiaris OX=9615 GN=PARK7 PE=4 SV=1 | A0A8I3PTM6 | *PARK7* | 29 kDa | 0.59 | 0.229148 | 1.0604672 | 0.0847 |
| **506** | Uncharacterized protein OS=Canis lupus familiaris OX=9615 GN=LOC479459 PE=3 SV=1 | A0A8I3MGN3 | *LOC479459* | 26 kDa | 0.6 | 0.2218487 | 0.423372656 | -1.24 |
| **507** | Tropomyosin 2 OS=Canis lupus familiaris OX=9615 GN=TPM2 PE=3 SV=1 | A0A8I3RWJ7 | *TPM2* | 33 kDa | 0.6 | 0.2218487 | 0.636397468 | -0.652 |
| **508** | AP complex subunit beta OS=Canis lupus familiaris OX=9615 GN=AP2B1 PE=3 SV=1 | A0A8I3ND36 | *AP2B1* | 105 kDa | 0.6 | 0.2218487 | 0.742261785 | -0.43 |
| **509** | AP-2 complex subunit mu OS=Canis lupus familiaris OX=9615 GN=AP2M1 PE=3 SV=1 | A0A8I3PSX7 | *AP2M1* | 50 kDa | 0.6 | 0.2218487 | 0.848507902 | -0.237 |
| **510** | Ribonuclease inhibitor OS=Canis lupus familiaris OX=9615 GN=RNH1 PE=4 SV=1 | A0A8I3Q1Z0 | *RNH1* | 50 kDa | 0.6 | 0.2218487 | 0.994160026 | -0.00845 |
| **511** | Chromobox 3 OS=Canis lupus familiaris OX=9615 GN=CBX3 PE=4 SV=1 | A0A8I3NCM0 | *CBX3* | 23 kDa | 0.6 | 0.2218487 | 1.237132479 | 0.307 |
| **512** | Hepatocyte growth factor-regulated tyrosine kinase substrate OS=Canis lupus familiaris OX=9615 GN=HGS PE=4 SV=1 | A0A8I3N2P0 | *HGS* | 111 kDa | 0.61 | 0.2146702 | 0.530343871 | -0.915 |
| **513** | Importin 7 OS=Canis lupus familiaris OX=9615 GN=IPO7 PE=4 SV=1 | A0A8I3NQH3 | *IPO7* | 120 kDa | 0.61 | 0.2146702 | 0.60583633 | -0.723 |
| **514** | Lysosomal associated membrane protein 1 OS=Canis lupus familiaris OX=9615 GN=LAMP1 PE=3 SV=1 | A0A8I3PPW7 | *LAMP1* | 44 kDa | 0.61 | 0.2146702 | 0.636397468 | -0.652 |
| **515** | Drebrin 1 OS=Canis lupus familiaris OX=9615 GN=DBN1 PE=4 SV=1 | A0A8I3NEM0 | *DBN1* | 33 kDa | 0.61 | 0.2146702 | 0.734075318 | -0.446 |
| **516** | Clathrin heavy chain OS=Canis lupus familiaris OX=9615 GN=CLTC PE=3 SV=1 | A0A8I3PLG2 | *CLTC* | 185 kDa | 0.61 | 0.2146702 | 0.812815602 | -0.299 |
| **517** | Adenylyl cyclase-associated protein OS=Canis lupus familiaris OX=9615 GN=CAP1 PE=3 SV=1 | A0A8I3NG14 | *CAP1* | 51 kDa | 0.61 | 0.2146702 | 0.831045862 | -0.267 |
| **518** | Podocan OS=Canis lupus familiaris OX=9615 GN=PODN PE=4 SV=1 | A0A8I3P9X5 | *PODN* | 74 kDa | 0.61 | 0.2146702 | 0.954422527 | -0.0673 |
| **519** | Aminopeptidase OS=Canis lupus familiaris OX=9615 GN=NPEPPS PE=3 SV=1 | A0A8I3P4I9 | *NPEPPS* | 99 kDa | 0.61 | 0.2146702 | 0.968215436 | -0.0466 |
| **520** | Phosphoglycerate mutase OS=Canis lupus familiaris OX=9615 GN=PGAM2 PE=3 SV=1 | A0A8I3NE41 | *PGAM2* | 32 kDa | 0.61 | 0.2146702 | 0.978877342 | -0.0308 |
| **521** | Proteasome subunit beta OS=Canis lupus familiaris OX=9615 GN=PSMB6 PE=3 SV=1 | A0A8I3RT39 | *PSMB6* | 25 kDa | 0.61 | 0.2146702 | 1.001519145 | 0.00219 |
| **522** | peptidylprolyl isomerase OS=Canis lupus familiaris OX=9615 GN=LOC100686132 PE=4 SV=1 | A0A8I3S252 | *LOC100686132* | 21 kDa | 0.62 | 0.2076083 | 0.530343871 | -0.915 |
| **523** | Glyoxalase domain containing 4 OS=Canis lupus familiaris OX=9615 GN=GLOD4 PE=3 SV=1 | A0A8I3NSX2 | *GLOD4* | 33 kDa | 0.62 | 0.2076083 | 0.530343871 | -0.915 |
| **524** | Granulin precursor OS=Canis lupus familiaris OX=9615 GN=GRN PE=3 SV=1 | A0A8I3MYU9 | *GRN* | 63 kDa | 0.62 | 0.2076083 | 0.585199321 | -0.773 |

| | | | | | | | | |
|---|---|---|---|---|---|---|---|---|
| **525** | EGF containing fibulin extracellular matrix protein 2 OS=Canis lupus familiaris OX=9615 GN=EFEMP2 PE=4 SV=1 | A0A8I3S033 | *EFEMP2* | 50 kDa | 0.62 | 0.2076083 | 0.636397468 | -0.652 |
| **526** | Matrix metallopeptidase 2 OS=Canis lupus familiaris OX=9615 GN=MMP2 PE=3 SV=1 | A0A8I3MGS0 | *MMP2* | 74 kDa | 0.62 | 0.2076083 | 0.86154616 | -0.215 |
| **527** | Triosephosphate isomerase OS=Canis lupus familiaris OX=9615 GN=TPI1 PE=3 SV=1 | A0A8I3PRM7 | *TPI1* | 31 kDa | 0.62 | 0.2076083 | 0.991304373 | -0.0126 |
| **528** | RAB8A, member RAS onco family OS=Canis lupus familiaris OX=9615 GN=RAB8A PE=3 SV=1 | A0A8I3Q6P3 | *RAB8A* | 29 kDa | 0.62 | 0.2076083 | 1.0604672 | 0.0847 |
| **529** | PDGFA associated protein 1 OS=Canis lupus familiaris OX=9615 GN=PDAP1 PE=4 SV=1 | A0A8I3MQ69 | *PDAP1* | 21 kDa | 0.62 | 0.2076083 | 1.767855062 | 0.822 |
| **530** | Uncharacterized protein OS=Canis lupus familiaris OX=9615 GN=SUGT1 PE=3 SV=1 | A0A8I3ND03 | *SUGT1* | 38 kDa | 0.63 | 0.2006595 | 0.636397468 | -0.652 |
| **531** | 40S ribosomal protein S6 OS=Canis lupus familiaris OX=9615 GN=RPS6 PE=3 SV=1 | A0A8I3NUU7 | *RPS6* | 29 kDa | 0.63 | 0.2006595 | 0.636397468 | -0.652 |
| **532** | VAMP associated protein A OS=Canis lupus familiaris OX=9615 GN=VAPA PE=3 SV=1 | A0A8I3MT19 | *VAPA* | 28 kDa | 0.63 | 0.2006595 | 0.757333158 | -0.401 |
| **533** | Phosphoserine aminotransferase OS=Canis lupus familiaris OX=9615 GN=PSAT1 PE=3 SV=1 | A0A8I3RUC9 | *PSAT1* | 47 kDa | 0.63 | 0.2006595 | 0.764718139 | -0.387 |
| **534** | Complement C1r OS=Canis lupus familiaris OX=9615 GN=C1R PE=4 SV=1 | A0A8I3Q683 | *C1R* | 77 kDa | 0.63 | 0.2006595 | 0.795536484 | -0.33 |
| **535** | Coatomer subunit gamma OS=Canis lupus familiaris OX=9615 GN=COPG1 PE=3 SV=1 | A0A8I3PBX4 | *COPG1* | 90 kDa | 0.63 | 0.2006595 | 0.848507902 | -0.237 |
| **536** | Renin receptor OS=Canis lupus familiaris OX=9615 GN=ATP6AP2 PE=4 SV=1 | A0A8I3PTV1 | *ATP6AP2* | 39 kDa | 0.63 | 0.2006595 | 0.908778116 | -0.138 |
| **537** | Nucleoside diphosphate kinase A OS=Canis lupus familiaris OX=9615 GN=NME1 PE=2 SV=1 | Q50KA9 | *NME1* | 17 kDa | 0.63 | 0.2006595 | 0.918912883 | -0.122 |
| **538** | Coronin OS=Canis lupus familiaris OX=9615 GN=CORO1C PE=3 SV=1 | A0A8I3PZZ4 | *CORO1C* | 53 kDa | 0.63 | 0.2006595 | 0.927873476 | -0.108 |
| **539** | Versican OS=Canis lupus familiaris OX=9615 GN=VCAN PE=3 SV=1 | A0A8I3PBG9 | *VCAN* | 366 kDa | 0.63 | 0.2006595 | 0.954422527 | -0.0673 |
| **540** | phosphopyruvate hydratase OS=Canis lupus familiaris OX=9615 GN=ENO1 PE=3 SV=1 | A0A8I3PWZ2 | *ENO1* | 47 kDa | 0.63 | 0.2006595 | 0.964063446 | -0.0528 |
| **541** | Vinculin OS=Canis lupus familiaris OX=9615 GN=VCL PE=3 SV=1 | A0A8I3NBQ1 | *VCL* | 113 kDa | 0.63 | 0.2006595 | 0.969357005 | -0.0449 |
| **542** | Calmodulin 2 OS=Canis lupus familiaris OX=9615 GN=CALM1 PE=4 SV=1 | E2REK6 | *CALM1* | 17 kDa | 0.63 | 0.2006595 | 1.0604672 | 0.0847 |
| **543** | Eukaryotic translation initiation factor 3 subunit H OS=Canis lupus familiaris OX=9615 GN=EIF3H PE=3 SV=1 | A0A8I3NST4 | *EIF3H* | 41 kDa | 0.63 | 0.2006595 | 1.272794935 | 0.348 |
| **544** | Uncharacterized protein OS=Canis lupus familiaris OX=9615 PE=4 SV=1 | A0A8I3Q8Q7 | | 22 kDa | 0.63 | 0.2006595 | 1.484523571 | 0.57 |
| **545** | EMAP like 2 OS=Canis lupus familiaris OX=9615 GN=EML2 PE=3 SV=1 | A0A8I3RTW8 | *EML2* | 73 kDa | 0.64 | 0.19382 | 0.883927531 | -0.178 |
| **546** | Myelin protein zero like 1 OS=Canis lupus familiaris OX=9615 GN=MPZL1 PE=4 SV=1 | A0A8I3MQS7 | *MPZL1* | 29 kDa | 0.64 | 0.19382 | 0.94258851 | -0.0853 |
| **547** | Phosphatidylethanolamine binding protein 1 OS=Canis lupus familiaris OX=9615 GN=PEBP1 PE=3 SV=1 | A0A8I3SAZ0 | *PEBP1* | 27 kDa | 0.64 | 0.19382 | 0.984661667 | -0.0223 |
| **548** | SH3 domain binding glutamate rich protein like 3 OS=Canis lupus familiaris OX=9615 GN=SH3BGRL3 PE=3 SV=1 | A0A8I3NHK0 | *SH3BGRL3* | 9 kDa | 0.64 | 0.19382 | 1.0604672 | 0.0847 |

| | | | | | | | | |
|---|---|---|---|---|---|---|---|---|
| **549** | Capping actin protein, gelsolin like OS=Canis lupus familiaris OX=9615 GN=CAPG PE=4 SV=1 | A0A8I3NNU8 | *CAPG* | 45 kDa | 0.64 | 0.19382 | 1.0604672 | 0.0847 |
| **550** | Collagen type III alpha 1 chain OS=Canis lupus familiaris OX=9615 GN=COL3A1 PE=4 SV=1 | A0A8I3S1I9 | *COL3A1* | 136 kDa | 0.64 | 0.19382 | 1.204972315 | 0.269 |
| **551** | Eukaryotic translation initiation factor 3 subunit D OS=Canis lupus familiaris OX=9615 GN=EIF3D PE=3 SV=1 | A0A8I3NBD4 | *EIF3D* | 65 kDa | 0.64 | 0.19382 | 1.21167266 | 0.277 |
| **552** | PDZ and LIM domain 5 OS=Canis lupus familiaris OX=9615 GN=PDLIM5 PE=4 SV=1 | A0A8I3PM75 | *PDLIM5* | 64 kDa | 0.64 | 0.19382 | 1.32592576 | 0.407 |
| **553** | Lymphocyte cytosolic protein 1 OS=Canis lupus familiaris OX=9615 GN=LCP1 PE=4 SV=1 | A0A8I3MXC1 | *LCP1* | 71 kDa | 0.64 | 0.19382 | 1.414213562 | 0.5 |
| **554** | Heterogeneous nuclear ribonucleoprotein A/B OS=Canis lupus familiaris OX=9615 GN=HNRNPAB PE=4 SV=1 | A0A8I3N8K2 | *HNRNPAB* | 31 kDa | 0.65 | 0.1870866 | 0.482968164 | -1.05 |
| **555** | Serine/arginine-rich splicing factor 1 OS=Canis lupus familiaris OX=9615 GN=SRSF1 PE=3 SV=1 | A0A8I3P1V7 | *SRSF1* | 28 kDa | 0.65 | 0.1870866 | 0.757333158 | -0.401 |
| **556** | Integrin subunit beta like 1 OS=Canis lupus familiaris OX=9615 GN=ITGBL1 PE=4 SV=1 | A0A8I3PXW7 | *ITGBL1* | 54 kDa | 0.65 | 0.1870866 | 0.795536484 | -0.33 |
| **557** | Radixin OS=Canis lupus familiaris OX=9615 GN=RDX PE=4 SV=1 | A0A8I3MN54 | *RDX* | 69 kDa | 0.65 | 0.1870866 | 1.027330228 | 0.0389 |
| **558** | ADF-H domain-containing protein OS=Canis lupus familiaris OX=9615 GN=DSTN PE=3 SV=1 | A0A8I3P8P0 | *DSTN* | 32 kDa | 0.65 | 0.1870866 | 1.0604672 | 0.0847 |
| **559** | Large ribosomal subunit protein uL4 OS=Canis lupus familiaris OX=9615 GN=RPL4 PE=3 SV=1 | A0A8I3P247 | *RPL4* | 47 kDa | 0.65 | 0.1870866 | 1.0604672 | 0.0847 |
| **560** | Fibronectin type-III domain-containing protein OS=Canis lupus familiaris OX=9615 PE=4 SV=1 | A0A8I3NTW0 | | 159 kDa | 0.65 | 0.1870866 | 1.378405153 | 0.463 |
| **561** | KH-type splicing regulatory protein OS=Canis lupus familiaris OX=9615 GN=KHSRP PE=4 SV=1 | A0A8I3RYB2 | *KHSRP* | 77 kDa | 0.66 | 0.1804561 | 0.636397468 | -0.652 |
| **562** | Ubiquitin conjugating enzyme E2 V1 OS=Canis lupus familiaris OX=9615 GN=LOC119881719 PE=4 SV=1 | A0A8I3PX03 | *LOC119881719* | 12 kDa | 0.66 | 0.1804561 | 0.707106781 | -0.5 |
| **563** | Actin related protein 2 OS=Canis lupus familiaris OX=9615 GN=ACTR2 PE=3 SV=1 | A0A8I3S593 | *ACTR2* | 45 kDa | 0.66 | 0.1804561 | 0.757333158 | -0.401 |
| **564** | F-actin-capping protein subunit alpha OS=Canis lupus familiaris OX=9615 GN=LOC612266 PE=3 SV=1 | A0A8I3N7S9 | *LOC612266* | 25 kDa | 0.66 | 0.1804561 | 0.757333158 | -0.401 |
| **565** | Eukaryotic translation initiation factor 3 subunit B OS=Canis lupus familiaris OX=9615 GN=EIF3B PE=3 SV=1 | A0A8I3RTH2 | *EIF3B* | 92 kDa | 0.66 | 0.1804561 | 0.994160026 | -0.00845 |
| **566** | Protein S100-A10 OS=Canis lupus familiaris OX=9615 GN=S100A10 PE=4 SV=3 | E2RGM0 | *S100A10* | 11 kDa | 0.66 | 0.1804561 | 0.998054152 | -0.00281 |
| **567** | Large ribosomal subunit protein eL15 OS=Canis lupus familiaris OX=9615 GN=RPL15 PE=1 SV=1 | E2QXF3 | *RPL15* | 24 kDa | 0.66 | 0.1804561 | 1.0604672 | 0.0847 |
| **568** | Glyceraldehyde-3-phosphate dehydrogenase OS=Canis lupus familiaris OX=9615 GN=LOC100683724 PE=3 SV=1 | A0A8I3NPU2 | *LOC100683724* | 36 kDa | 0.66 | 0.1804561 | 1.133669413 | 0.181 |
| **569** | Myosin light chain kinase, smooth muscle OS=Canis lupus familiaris OX=9615 GN=MYLK PE=3 SV=1 | A0A8I3PTT4 | *MYLK* | 220 kDa | 0.67 | 0.1739252 | 0.60583633 | -0.723 |
| **570** | Lipocalin 2 OS=Canis lupus familiaris OX=9615 GN=LCN2 PE=3 SV=1 | A0A8I3Q9B0 | *LCN2* | 23 kDa | 0.67 | 0.1739252 | 0.60583633 | -0.723 |
| **571** | Fascin OS=Canis lupus familiaris OX=9615 GN=FSCN1 PE=3 SV=1 | A0A8I3NPK1 | *FSCN1* | 55 kDa | 0.67 | 0.1739252 | 0.757333158 | -0.401 |
| **572** | 40S ribosomal protein S11 N-terminal domain-containing protein OS=Canis lupus familiaris OX=9615 PE=4 SV=1 | A0A8I3RQY9 | | 21 kDa | 0.67 | 0.1739252 | 0.757333158 | -0.401 |

| | | | | | | | | |
|---|---|---|---|---|---|---|---|---|
| **573** | Lamin B1 OS=Canis lupus familiaris OX=9615 GN=LMNB1 PE=3 SV=1 | A0A8I3NBF9 | *LMNB1* | 67 kDa | 0.67 | 0.1739252 | 0.867538687 | -0.205 |
| **574** | X-prolyl aminopeptidase 1 OS=Canis lupus familiaris OX=9615 GN=XPNPEP1 PE=3 SV=1 | A0A8I3PV34 | *XPNPEP1* | 75 kDa | 0.67 | 0.1739252 | 0.883927531 | -0.178 |
| **575** | Myristoylated alanine rich protein kinase C substrate OS=Canis lupus familiaris OX=9615 GN=MARCKS PE=3 SV=1 | A0A8I3P9P7 | *MARCKS* | 34 kDa | 0.67 | 0.1739252 | 0.901250463 | -0.15 |
| **576** | Lactoylglutathione lyase OS=Canis lupus familiaris OX=9615 GN=GLO1 PE=3 SV=1 | A0A8I3NIZ1 | *GLO1* | 20 kDa | 0.67 | 0.1739252 | 0.927873476 | -0.108 |
| **577** | Peroxiredoxin-1 OS=Canis lupus familiaris OX=9615 GN=PRDX1 PE=3 SV=1 | A0A8I3S380 | *PRDX1* | 24 kDa | 0.67 | 0.1739252 | 1.0604672 | 0.0847 |
| **578** | Ubiquitin carboxyl-terminal hydrolase OS=Canis lupus familiaris OX=9615 GN=UCHL1 PE=3 SV=1 | A0A8I3MNG8 | *UCHL1* | 38 kDa | 0.67 | 0.1739252 | 1.247465572 | 0.319 |
| **579** | 40S ribosomal protein S7 OS=Canis lupus familiaris OX=9615 GN=RPS7 PE=3 SV=1 | A0A8I3PNN7 | *RPS7* | 22 kDa | 0.67 | 0.1739252 | 1.591072968 | 0.67 |
| **580** | BRO1 domain-containing protein OS=Canis lupus familiaris OX=9615 GN=PDCD6IP PE=4 SV=1 | A0A8I3PN55 | *PDCD6IP* | 95 kDa | 0.68 | 0.1674911 | 0.618566239 | -0.693 |
| **581** | Inositol-1-monophosphatase OS=Canis lupus familiaris OX=9615 GN=IMPA1 PE=3 SV=1 | A0A8I3NVV9 | *IMPA1* | 30 kDa | 0.68 | 0.1674911 | 0.795536484 | -0.33 |
| **582** | dimethylargininase OS=Canis lupus familiaris OX=9615 GN=DDAH2 PE=3 SV=1 | A0A8I3NHQ9 | *DDAH2* | 30 kDa | 0.68 | 0.1674911 | 0.795536484 | -0.33 |
| **583** | Cytosol aminopeptidase OS=Canis lupus familiaris OX=9615 GN=LAP3 PE=3 SV=1 | A0A8I3MBN3 | *LAP3* | 56 kDa | 0.68 | 0.1674911 | 0.854409741 | -0.227 |
| **584** | Small ribosomal subunit protein uS2 OS=Canis lupus familiaris OX=9615 GN=RPSA PE=3 SV=1 | A0A8I3NYW8 | *RPSA* | 33 kDa | 0.68 | 0.1674911 | 0.856781955 | -0.223 |
| **585** | Protein disulfide-isomerase OS=Canis lupus familiaris OX=9615 GN=P4HB PE=3 SV=1 | A0A8I3NBI7 | *P4HB* | 57 kDa | 0.68 | 0.1674911 | 0.867538687 | -0.205 |
| **586** | peptidylprolyl isomerase OS=Canis lupus familiaris OX=9615 GN=FKBP9 PE=4 SV=1 | A0A8I3PIH4 | *FKBP9* | 63 kDa | 0.68 | 0.1674911 | 0.883927531 | -0.178 |
| **587** | Dihydropyrimidinase like 2 OS=Canis lupus familiaris OX=9615 GN=DPYSL2 PE=3 SV=1 | A0A8I3NRS8 | *DPYSL2* | 62 kDa | 0.68 | 0.1674911 | 0.959463613 | -0.0597 |
| **588** | Protein kinase C substrate 80K-H OS=Canis lupus familiaris OX=9615 GN=PRKCSH PE=4 SV=1 | A0A8I3S0P1 | *PRKCSH* | 60 kDa | 0.68 | 0.1674911 | 1.591072968 | 0.67 |
| **589** | Glutathione-disulfide reductase OS=Canis lupus familiaris OX=9615 GN=GSR PE=3 SV=1 | A0A8I3NVE9 | *GSR* | 53 kDa | 0.69 | 0.1611509 | 0.530343871 | -0.915 |
| **590** | Microtubule associated protein 1A OS=Canis lupus familiaris OX=9615 GN=MAP1A PE=4 SV=1 | A0A8I3PLI8 | *MAP1A* | 329 kDa | 0.69 | 0.1611509 | 0.66296288 | -0.593 |
| **591** | UV excision repair protein RAD23 OS=Canis lupus familiaris OX=9615 GN=RAD23B PE=3 SV=1 | A0A8I3NK06 | *RAD23B* | 43 kDa | 0.69 | 0.1611509 | 0.707106781 | -0.5 |
| **592** | Thimet oligopeptidase 1 OS=Canis lupus familiaris OX=9615 GN=THOP1 PE=3 SV=1 | A0A8I3N7K6 | *THOP1* | 78 kDa | 0.69 | 0.1611509 | 0.707106781 | -0.5 |
| **593** | Septin OS=Canis lupus familiaris OX=9615 GN=SEPTIN7 PE=3 SV=1 | A0A8I3Q048 | *SEPTIN7* | 47 kDa | 0.69 | 0.1611509 | 0.707106781 | -0.5 |
| **594** | Alanine--tRNA ligase OS=Canis lupus familiaris OX=9615 GN=AARS1 PE=3 SV=1 | A0A8I3NAD6 | *AARS1* | 106 kDa | 0.69 | 0.1611509 | 0.720964436 | -0.472 |
| **595** | Vasorin OS=Canis lupus familiaris OX=9615 GN=VASN PE=4 SV=1 | A0A8I3MK79 | *VASN* | 71 kDa | 0.69 | 0.1611509 | 0.757333158 | -0.401 |
| **596** | inorganic diphosphatase OS=Canis lupus familiaris OX=9615 GN=PPA1 PE=3 SV=1 | A0A8I3MKA2 | *PPA1* | 35 kDa | 0.69 | 0.1611509 | 0.795536484 | -0.33 |

| | | | | | | | | |
|---|---|---|---|---|---|---|---|---|
| **597** | CSD domain-containing protein OS=Canis lupus familiaris OX=9615 PE=4 SV=1 | A0A8I3PF62 | | 36 kDa | 0.69 | 0.1611509 | 0.875998315 | -0.191 |
| **598** | Calumenin OS=Canis lupus familiaris OX=9615 GN=CALU PE=4 SV=1 | A0A8I3PU96 | *CALU* | 37 kDa | 0.69 | 0.1611509 | 0.921464186 | -0.118 |
| **599** | Phosphoglucomutase 2 OS=Canis lupus familiaris OX=9615 GN=PGM2 PE=3 SV=1 | A0A8I3MSC7 | *PGM2* | 68 kDa | 0.69 | 0.1611509 | 1.0604672 | 0.0847 |
| **600** | Fructose-bisphosphate aldolase OS=Canis lupus familiaris OX=9615 GN=ALDOA PE=3 SV=1 | A0A8I3MJP4 | *ALDOA* | 45 kDa | 0.69 | 0.1611509 | 1.100378609 | 0.138 |
| **601** | Phosphoglycerate kinase OS=Canis lupus familiaris OX=9615 GN=PGK1 PE=3 SV=1 | A0A8I3P9Y9 | *PGK1* | 44 kDa | 0.69 | 0.1611509 | 1.199139914 | 0.262 |
| **602** | Cadherin 13 OS=Canis lupus familiaris OX=9615 GN=CDH13 PE=4 SV=1 | A0A8I3RZN1 | *CDH13* | 74 kDa | 0.69 | 0.1611509 | 1.414213562 | 0.5 |
| **603** | SUMO-activating enzyme subunit 2 OS=Canis lupus familiaris OX=9615 GN=UBA2 PE=3 SV=1 | A0A8I3MRU4 | *UBA2* | 65 kDa | 0.7 | 0.154902 | 0.318640157 | -1.65 |
| **604** | Annexin OS=Canis lupus familiaris OX=9615 GN=ANXA6 PE=3 SV=1 | A0A8I3N098 | *ANXA6* | 76 kDa | 0.7 | 0.154902 | 0.378929142 | -1.4 |
| **605** | Olfactomedin like 2B OS=Canis lupus familiaris OX=9615 GN=OLFML2B PE=4 SV=1 | A0A8I3PU13 | *OLFML2B* | 78 kDa | 0.7 | 0.154902 | 0.530343871 | -0.915 |
| **606** | Protein tyrosine kinase 7 (inactive) OS=Canis lupus familiaris OX=9615 GN=PTK7 PE=4 SV=1 | A0A8I3N574 | *PTK7* | 118 kDa | 0.7 | 0.154902 | 0.725476104 | -0.463 |
| **607** | Regulator of chromosome condensation 2 OS=Canis lupus familiaris OX=9615 GN=RCC2 PE=4 SV=1 | A0A8I3MWB8 | *RCC2* | 56 kDa | 0.7 | 0.154902 | 0.795536484 | -0.33 |
| **608** | T-complex protein 1 subunit gamma OS=Canis lupus familiaris OX=9615 GN=CCT3 PE=3 SV=1 | A0A8I3N2L4 | *CCT3* | 57 kDa | 0.7 | 0.154902 | 0.90000193 | -0.152 |
| **609** | glutamate dehydrogenase [NAD(P)(+)] OS=Canis lupus familiaris OX=9615 GN=GLUD1 PE=3 SV=1 | A0A8I3N1X6 | *GLUD1* | 61 kDa | 0.7 | 0.154902 | 1.0604672 | 0.0847 |
| **610** | Periostin OS=Canis lupus familiaris OX=9615 GN=POSTN PE=4 SV=1 | A0A8I3PVD8 | *POSTN* | 93 kDa | 0.71 | 0.1487417 | 0.466516496 | -1.1 |
| **611** | cysteine--tRNA ligase OS=Canis lupus familiaris OX=9615 GN=CARS1 PE=3 SV=1 | A0A8I3NCB2 | *CARS1* | 95 kDa | 0.71 | 0.1487417 | 0.652477474 | -0.616 |
| **612** | Nudix hydrolase 5 OS=Canis lupus familiaris OX=9615 GN=NUDT5 PE=3 SV=1 | A0A8I3MAJ3 | *NUDT5* | 24 kDa | 0.71 | 0.1487417 | 0.66296288 | -0.593 |
| **613** | Cathepsin D OS=Canis lupus familiaris OX=9615 GN=CTSD PE=3 SV=1 | A0A8I3N9F5 | *CTSD* | 43 kDa | 0.71 | 0.1487417 | 0.757333158 | -0.401 |
| **614** | Small ribosomal subunit protein uS3 OS=Canis lupus familiaris OX=9615 GN=RPS3 PE=1 SV=1 | E2RH47 | *RPS3* | 27 kDa | 0.71 | 0.1487417 | 0.795536484 | -0.33 |
| **615** | Eukaryotic translation initiation factor 3 subunit L OS=Canis lupus familiaris OX=9615 GN=EIF3L PE=3 SV=1 | A0A8I3NBV0 | *EIF3L* | 61 kDa | 0.71 | 0.1487417 | 0.795536484 | -0.33 |
| **616** | UPAR/Ly6 domain-containing protein OS=Canis lupus familiaris OX=9615 PE=4 SV=1 | A0A8I3N922 | | 32 kDa | 0.71 | 0.1487417 | 0.829894586 | -0.269 |
| **617** | F-actin-capping protein subunit beta OS=Canis lupus familiaris OX=9615 GN=CAPZB PE=3 SV=1 | A0A8I3MQR3 | *CAPZB* | 31 kDa | 0.71 | 0.1487417 | 0.837406488 | -0.256 |
| **618** | IFI30 lysosomal thiol reductase OS=Canis lupus familiaris OX=9615 GN=IFI30 PE=3 SV=1 | A0A8I3PSI0 | *IFI30* | 28 kDa | 0.71 | 0.1487417 | 0.908778116 | -0.138 |
| **619** | Peptidase D OS=Canis lupus familiaris OX=9615 GN=PEPD PE=3 SV=1 | A0A8I3RNY0 | *PEPD* | 55 kDa | 0.71 | 0.1487417 | 0.927873476 | -0.108 |
| **620** | Uncharacterized protein OS=Canis lupus familiaris OX=9615 GN=FLNA PE=3 SV=1 | A0A8I3Q522 | *FLNA* | 277 kDa | 0.71 | 0.1487417 | 0.927873476 | -0.108 |

| | | | | | | | | |
|---|---|---|---|---|---|---|---|---|
| **621** | Polypyrimidine tract-binding protein 1 OS=Canis lupus familiaris OX=9615 GN=PTBP1 PE=4 SV=1 | A0A8I3NY06 | *PTBP1* | 57 kDa | 0.71 | 0.1487417 | 0.94258851 | -0.0853 |
| **622** | Actinin alpha 1 OS=Canis lupus familiaris OX=9615 GN=ACTN1 PE=3 SV=1 | A0A8I3MVW9 | *ACTN1* | 105 kDa | 0.71 | 0.1487417 | 0.974206828 | -0.0377 |
| **623** | Galactokinase 1 OS=Canis lupus familiaris OX=9615 GN=H3-3B PE=3 SV=1 | A0A8I3PKD4 | *H3-3B* | 47 kDa | 0.71 | 0.1487417 | 0.998054152 | -0.00281 |
| **624** | Peptidyl-prolyl cis-trans isomerase OS=Canis lupus familiaris OX=9615 PE=3 SV=1 | A0A8I3P0E7 | | 16 kDa | 0.71 | 0.1487417 | 1.012203943 | 0.0175 |
| **625** | phosphopyruvate hydratase OS=Canis lupus familiaris OX=9615 PE=3 SV=1 | A0A8I3N1E7 | | 41 kDa | 0.71 | 0.1487417 | 1.037851477 | 0.0536 |
| **626** | WD repeat domain 1 OS=Canis lupus familiaris OX=9615 GN=WDR1 PE=4 SV=1 | A0A8I3MMG9 | *WDR1* | 66 kDa | 0.71 | 0.1487417 | 1.040805146 | 0.0577 |
| **627** | Density-regulated protein OS=Canis lupus familiaris OX=9615 GN=DENR PE=3 SV=1 | A0A8I3S4F4 | *DENR* | 29 kDa | 0.72 | 0.1426675 | 0.530343871 | -0.915 |
| **628** | deoxyribonuclease II OS=Canis lupus familiaris OX=9615 GN=DNASE2 PE=3 SV=1 | A0A8I3P5U3 | *DNASE2* | 40 kDa | 0.72 | 0.1426675 | 0.636397468 | -0.652 |
| **629** | UDP-glucose 6-dehydrogenase OS=Canis lupus familiaris OX=9615 GN=UGDH PE=3 SV=1 | A0A8I3MN63 | *UGDH* | 48 kDa | 0.72 | 0.1426675 | 0.636397468 | -0.652 |
| **630** | Cytochrome b5 type A (microsomal) OS=Canis lupus familiaris OX=9615 GN=CYB5A PE=3 SV=1 | A0A8I3RR60 | *CYB5A* | 14 kDa | 0.72 | 0.1426675 | 0.795536484 | -0.33 |
| **631** | Proteasome 20S subunit alpha 7 OS=Canis lupus familiaris OX=9615 PE=4 SV=1 | A0A8I3NBV3 | | 19 kDa | 0.72 | 0.1426675 | 0.848507902 | -0.237 |
| **632** | Annexin OS=Canis lupus familiaris OX=9615 GN=ANXA5 PE=2 SV=1 | A0A0F7RNT6 | *ANXA5* | 36 kDa | 0.72 | 0.1426675 | 0.873572896 | -0.195 |
| **633** | AE binding protein 1 OS=Canis lupus familiaris OX=9615 GN=AEBP1 PE=3 SV=1 | A0A8I3MT76 | *AEBP1* | 149 kDa | 0.72 | 0.1426675 | 0.908778116 | -0.138 |
| **634** | Heat shock protein 90 beta family member 1 OS=Canis lupus familiaris OX=9615 GN=HSP90B1 PE=3 SV=1 | A0A8I3NHC4 | *HSP90B1* | 94 kDa | 0.72 | 0.1426675 | 0.94881584 | -0.0758 |
| **635** | protein disulfide-isomerase OS=Canis lupus familiaris OX=9615 GN=P4HB PE=3 SV=1 | A0A8I3NBL0 | *P4HB* | 53 kDa | 0.72 | 0.1426675 | 1.100378609 | 0.138 |
| **636** | UBC core domain-containing protein OS=Canis lupus familiaris OX=9615 PE=4 SV=1 | A0A8I3PBI6 | | 14 kDa | 0.72 | 0.1426675 | 1.272794935 | 0.348 |
| **637** | Drebrin like OS=Canis lupus familiaris OX=9615 GN=DBNL PE=3 SV=1 | A0A8I3N0H1 | *DBNL* | 43 kDa | 0.72 | 0.1426675 | 1.591072968 | 0.67 |
| **638** | Alpha-1,4 glucan phosphorylase OS=Canis lupus familiaris OX=9615 GN=PYGL PE=3 SV=1 | A0A8I3N518 | *PYGL* | 103 kDa | 0.73 | 0.1366771 | 0.453759578 | -1.14 |
| **639** | Protein kinase cAMP-dependent type I regulatory subunit alpha OS=Canis lupus familiaris OX=9615 GN=PRKAR1A PE=3 SV=1 | A0A8I3Q3C0 | *PRKAR1A* | 42 kDa | 0.73 | 0.1366771 | 0.66296288 | -0.593 |
| **640** | Leucine rich repeat containing 59 OS=Canis lupus familiaris OX=9615 GN=LRRC59 PE=4 SV=1 | A0A8I3NJR1 | *LRRC59* | 35 kDa | 0.73 | 0.1366771 | 0.681601304 | -0.553 |
| **641** | Charged multivesicular body protein 4B OS=Canis lupus familiaris OX=9615 GN=CHMP4B PE=3 SV=1 | A0A8I3PLN8 | *CHMP4B* | 25 kDa | 0.73 | 0.1366771 | 0.848507902 | -0.237 |
| **642** | Serpin H1 OS=Canis lupus familiaris OX=9615 GN=SERPINH1 PE=3 SV=1 | A0A8I3NIH9 | *SERPINH1* | 54 kDa | 0.73 | 0.1366771 | 0.902500727 | -0.148 |
| **643** | thioredoxin-disulfide reductase OS=Canis lupus familiaris OX=9615 GN=TXNRD1 PE=3 SV=1 | A0A8I3RUW0 | *TXNRD1* | 71 kDa | 0.73 | 0.1366771 | 1.0604672 | 0.0847 |
| **644** | Galectin-3-binding protein OS=Canis lupus familiaris OX=9615 GN=CANT1 PE=3 SV=1 | A0A8I3NHK6 | *CANT1* | 79 kDa | 0.73 | 0.1366771 | 1.292352831 | 0.37 |

| | | | | | | | | |
|---|---|---|---|---|---|---|---|---|
| **645** | Small ribosomal subunit protein uS4 OS=Canis lupus familiaris OX=9615 GN=RPS9 PE=3 SV=1 | A0A8I3RS69 | *RPS9* | 23 kDa | 0.74 | 0.1307683 | 0.397768242 | -1.33 |
| **646** | Glutaredoxin 3 OS=Canis lupus familiaris OX=9615 GN=GLRX3 PE=4 SV=1 | A0A8I3SBI2 | *GLRX3* | 38 kDa | 0.74 | 0.1307683 | 0.636397468 | -0.652 |
| **647** | 60S ribosomal protein L7 OS=Canis lupus familiaris OX=9615 PE=3 SV=1 | A0A8I3PX95 | | 22 kDa | 0.74 | 0.1307683 | 0.707106781 | -0.5 |
| **648** | Cytidine/uridine monophosphate kinase 1 OS=Canis lupus familiaris OX=9615 GN=CMPK1 PE=3 SV=1 | A0A8I3NZ20 | *CMPK1* | 20 kDa | 0.74 | 0.1307683 | 0.707106781 | -0.5 |
| **649** | Tropomodulin 3 OS=Canis lupus familiaris OX=9615 GN=TMOD2 PE=4 SV=1 | A0A8I3S5X2 | *TMOD2* | 40 kDa | 0.74 | 0.1307683 | 0.734075318 | -0.446 |
| **650** | Small ribosomal subunit protein RACK1 OS=Canis lupus familiaris OX=9615 GN=RACK1 PE=3 SV=1 | A0A8I3RSQ2 | *RACK1* | 35 kDa | 0.74 | 0.1307683 | 0.767905135 | -0.381 |
| **651** | Uncharacterized protein OS=Canis lupus familiaris OX=9615 GN=PPP2R1A PE=4 SV=1 | A0A8I3MLL5 | *PPP2R1A* | 65 kDa | 0.74 | 0.1307683 | 0.777546036 | -0.363 |
| **652** | Uncharacterized protein OS=Canis lupus familiaris OX=9615 GN=KPNB1 PE=3 SV=1 | A0A8I3NZP6 | *KPNB1* | 97 kDa | 0.74 | 0.1307683 | 0.795536484 | -0.33 |
| **653** | Parathymosin OS=Canis lupus familiaris OX=9615 GN=PTMS PE=3 SV=1 | A0A8I3Q2X8 | *PTMS* | 12 kDa | 0.74 | 0.1307683 | 0.848507902 | -0.237 |
| **654** | LIM and SH3 domain protein 1 OS=Canis lupus familiaris OX=9615 GN=LASP1 PE=4 SV=1 | A0A8I3RXT2 | *LASP1* | 36 kDa | 0.74 | 0.1307683 | 0.908778116 | -0.138 |
| **655** | Ribosomal protein OS=Canis lupus familiaris OX=9615 GN=RPL10A PE=3 SV=1 | A0A8I3MZL8 | *RPL10A* | 25 kDa | 0.74 | 0.1307683 | 0.927873476 | -0.108 |
| **656** | Protein disulfide-isomerase A4 OS=Canis lupus familiaris OX=9615 GN=PDIA4 PE=3 SV=1 | A0A8I3RYV6 | *PDIA4* | 72 kDa | 0.74 | 0.1307683 | 0.989725257 | -0.0149 |
| **657** | 60S ribosomal protein L7a OS=Canis lupus familiaris OX=9615 GN=RPL7A PE=3 SV=1 | A0A8I3NP99 | *RPL7A* | 30 kDa | 0.74 | 0.1307683 | 1.009961291 | 0.0143 |
| **658** | REVERSE_A0A8I3MQ67 | REVERSE_A0A8I3MQ67 | | | 0.74 | 0.1307683 | 1.0604672 | 0.0847 |
| **659** | Secreted frizzled-related protein 2 OS=Canis lupus familiaris OX=9615 GN=SFRP2 PE=2 SV=1 | Q863H1 | *SFRP2* | 33 kDa | 0.74 | 0.1307683 | 1.16634937 | 0.222 |
| **660** | Myosin heavy chain 9 OS=Canis lupus familiaris OX=9615 GN=MYH9 PE=3 SV=1 | A0A8I3N9N7 | *MYH9* | 226 kDa | 0.74 | 0.1307683 | 1.180992661 | 0.24 |
| **661** | Parvin alpha OS=Canis lupus familiaris OX=9615 GN=PARVA PE=3 SV=1 | A0A8I3P5Q8 | *PARVA* | 38 kDa | 0.75 | 0.1249387 | 0.469761375 | -1.09 |
| **662** | Apolipoprotein B OS=Canis lupus familiaris OX=9615 GN=APOB PE=4 SV=1 | A0A8I3S4D1 | *APOB* | 504 kDa | 0.75 | 0.1249387 | 0.530343871 | -0.915 |
| **663** | Farnesyl pyrophosphate synthase OS=Canis lupus familiaris OX=9615 GN=FDPS PE=3 SV=1 | A0A8I3NF82 | *FDPS* | 48 kDa | 0.75 | 0.1249387 | 0.636397468 | -0.652 |
| **664** | Ferritin heavy chain OS=Canis lupus familiaris OX=9615 GN=FTH1 PE=2 SV=3 | Q95MP7 | *FTH1* | 21 kDa | 0.75 | 0.1249387 | 0.777546036 | -0.363 |
| **665** | Nucleolin OS=Canis lupus familiaris OX=9615 GN=NCL PE=4 SV=1 | A0A8I3PNX7 | *NCL* | 77 kDa | 0.75 | 0.1249387 | 0.812815602 | -0.299 |
| **666** | Insulin-like growth factor-binding protein 5 OS=Canis lupus familiaris OX=9615 GN=IGFBP5 PE=4 SV=1 | A0A8I3PP66 | *IGFBP5* | 30 kDa | 0.75 | 0.1249387 | 0.946844883 | -0.0788 |
| **667** | Glucose-6-phosphate isomerase OS=Canis lupus familiaris OX=9615 GN=GPI PE=3 SV=1 | A0A8I3RRW0 | *GPI* | 64 kDa | 0.75 | 0.1249387 | 1.0604672 | 0.0847 |
| **668** | Plectin OS=Canis lupus familiaris OX=9615 GN=PLEC PE=4 SV=1 | A0A8I3RTC1 | *PLEC* | 536 kDa | 0.75 | 0.1249387 | 1.076986376 | 0.107 |

| | | | | | | | | |
|---|---|---|---|---|---|---|---|---|
| **669** | Macrophage colony-stimulating factor 1 OS=Canis lupus familiaris OX=9615 GN=CSF1 PE=4 SV=1 | A0A8I3MKU0 | *CSF1* | 60 kDa | 0.75 | 0.1249387 | 1.193335743 | 0.255 |
| **670** | Platelet-activating factor acetylhydrolase IB subunit alpha OS=Canis lupus familiaris OX=9615 GN=PAFAH1B1 PE=3 SV=1 | A0A8I3NR37 | *PAFAH1B1* | 43 kDa | 0.76 | 0.1191864 | 0.571173123 | -0.808 |
| **671** | Eukaryotic translation initiation factor 4 gamma 1 OS=Canis lupus familiaris OX=9615 GN=EIF4G1 PE=3 SV=1 | A0A8I3SB22 | *EIF4G1* | 177 kDa | 0.76 | 0.1191864 | 0.707106781 | -0.5 |
| **672** | Eukaryotic peptide chain release factor subunit 1 OS=Canis lupus familiaris OX=9615 GN=ETF1 PE=3 SV=1 | A0A8I3PCV3 | *ETF1* | 49 kDa | 0.76 | 0.1191864 | 0.707106781 | -0.5 |
| **673** | NPC intracellular cholesterol transporter 2 OS=Canis lupus familiaris OX=9615 GN=NPC2 PE=3 SV=1 | A0A8I3NVN8 | *NPC2* | 16 kDa | 0.76 | 0.1191864 | 0.811127156 | -0.302 |
| **674** | Pyridoxal kinase OS=Canis lupus familiaris OX=9615 GN=PDXK PE=3 SV=1 | A0A8I3NWJ0 | *PDXK* | 32 kDa | 0.76 | 0.1191864 | 0.848507902 | -0.237 |
| **675** | AE binding protein 1 OS=Canis lupus familiaris OX=9615 GN=AEBP1 PE=3 SV=1 | A0A8I3N3T6 | *AEBP1* | 145 kDa | 0.76 | 0.1191864 | 0.87175824 | -0.198 |
| **676** | Caldesmon 1 OS=Canis lupus familiaris OX=9615 GN=CALD1 PE=4 SV=1 | A0A8I3RXG5 | *CALD1* | 87 kDa | 0.76 | 0.1191864 | 0.908778116 | -0.138 |
| **677** | Ribosomal protein S14 OS=Canis lupus familiaris OX=9615 GN=RPS14 PE=3 SV=1 | A0A8I3N3W6 | *RPS14* | 17 kDa | 0.76 | 0.1191864 | 0.927873476 | -0.108 |
| **678** | Filamin B OS=Canis lupus familiaris OX=9615 GN=FLNB PE=3 SV=1 | A0A8I3S4J9 | *FLNB* | 261 kDa | 0.76 | 0.1191864 | 0.96989468 | -0.0441 |
| **679** | Eukaryotic translation initiation factor 2 subunit 1 OS=Canis lupus familiaris OX=9615 GN=EIF2S1 PE=3 SV=1 | A0A8I3MV19 | *EIF2S1* | 36 kDa | 0.76 | 0.1191864 | 1.0604672 | 0.0847 |
| **680** | Talin 2 OS=Canis lupus familiaris OX=9615 GN=TLN2 PE=4 SV=1 | A0A8I3S166 | *TLN2* | 272 kDa | 0.76 | 0.1191864 | 1.219255094 | 0.286 |
| **681** | Nuclear autoantigenic sperm protein OS=Canis lupus familiaris OX=9615 GN=NASP PE=3 SV=1 | A0A8I3PMD4 | *NASP* | 113 kDa | 0.77 | 0.1135093 | 0.707106781 | -0.5 |
| **682** | Glutathione synthetase OS=Canis lupus familiaris OX=9615 GN=GSS PE=3 SV=1 | A0A8I3NS50 | *GSS* | 52 kDa | 0.77 | 0.1135093 | 0.795536484 | -0.33 |
| **683** | Heterogeneous nuclear ribonucleoprotein D OS=Canis lupus familiaris OX=9615 GN=HNRNPD PE=4 SV=1 | A0A8I3P1F3 | *HNRNPD* | 38 kDa | 0.77 | 0.1135093 | 0.848507902 | -0.237 |
| **684** | Nidogen 1 OS=Canis lupus familiaris OX=9615 GN=NID1 PE=4 SV=1 | A0A8I3MND5 | *NID1* | 122 kDa | 0.77 | 0.1135093 | 0.992748375 | -0.0105 |
| **685** | Coactosin like F-actin binding protein 1 OS=Canis lupus familiaris OX=9615 GN=COTL1 PE=4 SV=1 | A0A8I3RTP3 | *COTL1* | 25 kDa | 0.77 | 0.1135093 | 1.0604672 | 0.0847 |
| **686** | Collagen type XI alpha 1 chain OS=Canis lupus familiaris OX=9615 GN=COL11A1 PE=4 SV=1 | A0A8I3NGT1 | *COL11A1* | 167 kDa | 0.77 | 0.1135093 | 1.70408819 | 0.769 |
| **687** | Matrix remodeling-associated protein 8 OS=Canis lupus familiaris OX=9615 GN=MXRA8 PE=4 SV=1 | A0A8I3S4Y1 | *MXRA8* | 38 kDa | 0.78 | 0.1079054 | 0.530343871 | -0.915 |
| **688** | Uncharacterized protein OS=Canis lupus familiaris OX=9615 GN=LOC100686930 PE=3 SV=1 | A0A8I3S7B2 | *LOC100686930* | 48 kDa | 0.78 | 0.1079054 | 0.707106781 | -0.5 |
| **689** | Serpin family G member 1 OS=Canis lupus familiaris OX=9615 GN=SERPING1 PE=3 SV=1 | A0A8I3S1V7 | *SERPING1* | 56 kDa | 0.78 | 0.1079054 | 0.707106781 | -0.5 |
| **690** | Uncharacterized protein OS=Canis lupus familiaris OX=9615 GN=RANBP1 PE=4 SV=1 | A0A8I3PTM3 | *RANBP1* | 32 kDa | 0.78 | 0.1079054 | 0.795536484 | -0.33 |
| **691** | peptidylprolyl isomerase OS=Canis lupus familiaris OX=9615 GN=FKBP4 PE=2 SV=2 | E2QWF5 | *FKBP4* | 52 kDa | 0.78 | 0.1079054 | 0.848507902 | -0.237 |
| **692** | Elastin microfibril interfacer 1 OS=Canis lupus familiaris OX=9615 GN=EMILIN1 PE=4 SV=1 | A0A8I3RT07 | *EMILIN1* | 107 kDa | 0.78 | 0.1079054 | 0.877821798 | -0.188 |

| | | | | | | | | |
|---|---|---|---|---|---|---|---|---|
| **693** | Ribosomal protein L3 OS=Canis lupus familiaris OX=9615 GN=RPL3 PE=3 SV=1 | A0A8I3PMB8 | *RPL3* | 46 kDa | 0.78 | 0.1079054 | 0.908778116 | -0.138 |
| **694** | Uncharacterized protein OS=Canis lupus familiaris OX=9615 GN=FUBP1 PE=4 SV=1 | A0A8I3MQX9 | *FUBP1* | 65 kDa | 0.78 | 0.1079054 | 0.954422527 | -0.0673 |
| **695** | Cofilin 2 OS=Canis lupus familiaris OX=9615 GN=CFL2 PE=3 SV=1 | A0A8I3MEH0 | *CFL2* | 19 kDa | 0.78 | 0.1079054 | 1.018044059 | 0.0258 |
| **696** | hexokinase OS=Canis lupus familiaris OX=9615 GN=HK1 PE=3 SV=1 | A0A8I3MTA6 | *HK1* | 104 kDa | 0.78 | 0.1079054 | 1.0604672 | 0.0847 |
| **697** | Sushi, von Willebrand factor type A, EGF and pentraxin domain containing 1 OS=Canis lupus familiaris OX=9615 GN=SVEP1 PE=4 SV=1 | A0A8I3NS86 | *SVEP1* | 389 kDa | 0.79 | 0.1023729 | 0.47963206 | -1.06 |
| **698** | Lysine--tRNA ligase OS=Canis lupus familiaris OX=9615 GN=KARS1 PE=3 SV=1 | A0A8I3PI46 | *KARS1* | 72 kDa | 0.79 | 0.1023729 | 0.561360711 | -0.833 |
| **699** | T-complex protein 1 subunit alpha OS=Canis lupus familiaris OX=9615 GN=TCP1 PE=3 SV=1 | A0A8I3MJ68 | *TCP1* | 59 kDa | 0.79 | 0.1023729 | 0.596667872 | -0.745 |
| **700** | N-acetylneuraminate synthase OS=Canis lupus familiaris OX=9615 GN=NANS PE=4 SV=1 | A0A8I3N2X1 | *NANS* | 44 kDa | 0.79 | 0.1023729 | 0.848507902 | -0.237 |
| **701** | Protein S100 OS=Canis lupus familiaris OX=9615 GN=S100A4 PE=3 SV=1 | A0A8I3MM14 | *S100A4* | 14 kDa | 0.79 | 0.1023729 | 0.927873476 | -0.108 |
| **702** | 40S ribosomal protein S26 OS=Canis lupus familiaris OX=9615 GN=RPS26 PE=3 SV=1 | A0A8I3MXT9 | *RPS26* | 14 kDa | 0.79 | 0.1023729 | 0.927873476 | -0.108 |
| **703** | Filamin C OS=Canis lupus familiaris OX=9615 GN=FLNC PE=3 SV=1 | A0A8I3PJ64 | *FLNC* | 290 kDa | 0.79 | 0.1023729 | 1.008701984 | 0.0125 |
| **704** | Transketolase OS=Canis lupus familiaris OX=9615 GN=TKT PE=4 SV=1 | A0A8I3Q4Y1 | *TKT* | 73 kDa | 0.79 | 0.1023729 | 1.028327639 | 0.0403 |
| **705** | Out at first protein homolog OS=Canis lupus familiaris OX=9615 GN=OAF PE=3 SV=1 | A0A8I3P258 | *OAF* | 37 kDa | 0.79 | 0.1023729 | 1.0604672 | 0.0847 |
| **706** | Histone H4 OS=Canis lupus familiaris OX=9615 PE=3 SV=1 | A0A8I3PWE4 | | 10 kDa | 0.79 | 0.1023729 | 1.088242442 | 0.122 |
| **707** | Thioredoxin like 1 OS=Canis lupus familiaris OX=9615 GN=TXNL1 PE=4 SV=1 | A0A8I3MF49 | *TXNL1* | 28 kDa | 0.8 | 0.09691 | 0.453759578 | -1.14 |
| **708** | Retinoic acid receptor responder protein 2 OS=Canis lupus familiaris OX=9615 GN=RARRES2 PE=4 SV=1 | A0A8I3NN14 | *RARRES2* | 19 kDa | 0.8 | 0.09691 | 0.578344092 | -0.79 |
| **709** | Translation initiation factor IF2/IF5 domain-containing protein OS=Canis lupus familiaris OX=9615 GN=LOC612587 PE=4 SV=1 | A0A8I3S6C4 | *LOC612587* | 34 kDa | 0.8 | 0.09691 | 0.795536484 | -0.33 |
| **710** | glutathione transferase OS=Canis lupus familiaris OX=9615 GN=LOC479912 PE=3 SV=1 | A0A8I3RSA3 | *LOC479912* | 33 kDa | 0.8 | 0.09691 | 0.795536484 | -0.33 |
| **711** | Coatomer subunit beta' OS=Canis lupus familiaris OX=9615 GN=COPB2 PE=3 SV=1 | A0A8I3S666 | *COPB2* | 102 kDa | 0.8 | 0.09691 | 0.883927531 | -0.178 |
| **712** | Uncharacterized protein OS=Canis lupus familiaris OX=9615 GN=RAB11B PE=4 SV=1 | A0A8I3N1M4 | *RAB11B* | 20 kDa | 0.8 | 0.09691 | 1.148698355 | 0.2 |
| **713** | Calpain small subunit 1 OS=Canis lupus familiaris OX=9615 GN=CAPNS1 PE=4 SV=1 | A0A8I3MEZ7 | *CAPNS1* | 29 kDa | 0.8 | 0.09691 | 1.32592576 | 0.407 |
| **714** | cAMP-dependent protein kinase OS=Canis lupus familiaris OX=9615 GN=PRKACB PE=3 SV=1 | A0A8I3MHE8 | *PRKACB* | 39 kDa | 0.81 | 0.091515 | 0.453759578 | -1.14 |
| **715** | ABI family member 3 binding protein OS=Canis lupus familiaris OX=9615 GN=ABI3BP PE=4 SV=1 | A0A8I3NYT0 | *ABI3BP* | 129 kDa | 0.81 | 0.091515 | 0.66296288 | -0.593 |

| | | | | | | | | |
|---|---|---|---|---|---|---|---|---|
| **716** | Calponin OS=Canis lupus familiaris OX=9615 GN=CNN2 PE=3 SV=1 | A0A8I3NT50 | *CNN2* | 29 kDa | 0.81 | 0.091515 | 0.707106781 | -0.5 |
| **717** | Clathrin heavy chain OS=Canis lupus familiaris OX=9615 GN=CLTC PE=4 SV=1 | A0A8I3PUT4 | *CLTC* | 71 kDa | 0.81 | 0.091515 | 0.742261785 | -0.43 |
| **718** | Proteasome subunit alpha type OS=Canis lupus familiaris OX=9615 GN=PSMA4 PE=3 SV=1 | A0A8I3NP66 | *PSMA4* | 29 kDa | 0.81 | 0.091515 | 0.771105413 | -0.375 |
| **719** | Proliferation-associated 2G4 OS=Canis lupus familiaris OX=9615 GN=PA2G4 PE=3 SV=1 | A0A8I3MYE2 | *PA2G4* | 44 kDa | 0.81 | 0.091515 | 0.777546036 | -0.363 |
| **720** | Proteasome subunit beta OS=Canis lupus familiaris OX=9615 GN=PSMB7 PE=3 SV=1 | A0A8I3N5F1 | *PSMB7* | 34 kDa | 0.81 | 0.091515 | 0.867538687 | -0.205 |
| **721** | Annexin OS=Canis lupus familiaris OX=9615 GN=ANXA1 PE=3 SV=1 | A0A8I3M9P4 | *ANXA1* | 39 kDa | 0.81 | 0.091515 | 0.883927531 | -0.178 |
| **722** | Cadherin 2 OS=Canis lupus familiaris OX=9615 GN=CDH2 PE=4 SV=1 | A0A8I3NAG2 | *CDH2* | 100 kDa | 0.81 | 0.091515 | 0.922103118 | -0.117 |
| **723** | Myosin light chain 12A OS=Canis lupus familiaris OX=9615 GN=MYL12A PE=4 SV=1 | A0A8I3MIU0 | *MYL12A* | 20 kDa | 0.81 | 0.091515 | 1.0604672 | 0.0847 |
| **724** | Alpha-crystallin A chain OS=Canis lupus familiaris OX=9615 GN=HSPB6 PE=3 SV=1 | A0A8I3RRG1 | *HSPB6* | 17 kDa | 0.82 | 0.0861861 | 0.530343871 | -0.915 |
| **725** | Heterogeneous nuclear ribonucleoprotein D like OS=Canis lupus familiaris OX=9615 GN=HNRNPDL PE=4 SV=1 | A0A8I3S3S3 | *HNRNPDL* | 40 kDa | 0.82 | 0.0861861 | 0.795536484 | -0.33 |
| **726** | Heterogeneous nuclear ribonucleoprotein K OS=Canis lupus familiaris OX=9615 PE=4 SV=1 | A0A8I3S1J8 | | 48 kDa | 0.82 | 0.0861861 | 0.807760778 | -0.308 |
| **727** | Microtubule associated protein 1B OS=Canis lupus familiaris OX=9615 GN=MAP1B PE=4 SV=1 | A0A8I3RQP4 | *MAP1B* | 281 kDa | 0.82 | 0.0861861 | 0.927873476 | -0.108 |
| **728** | Prefoldin subunit 2 OS=Canis lupus familiaris OX=9615 GN=PFDN2 PE=3 SV=1 | A0A8I3Q7K8 | *PFDN2* | 17 kDa | 0.82 | 0.0861861 | 0.94258851 | -0.0853 |
| **729** | Latent transforming growth factor beta binding protein 2 OS=Canis lupus familiaris OX=9615 GN=LTBP2 PE=4 SV=1 | A0A8I3P2C0 | *LTBP2* | 195 kDa | 0.82 | 0.0861861 | 1.168777249 | 0.225 |
| **730** | C4a anaphylatoxin OS=Canis lupus familiaris OX=9615 GN=LOC481722 PE=4 SV=1 | A0A8I3NH20 | *LOC481722* | 195 kDa | 0.83 | 0.0809219 | 0.742261785 | -0.43 |
| **731** | Acetyl-CoA acetyltransferase 2 OS=Canis lupus familiaris OX=9615 GN=ACAT2 PE=3 SV=1 | A0A8I3N041 | *ACAT2* | 41 kDa | 0.83 | 0.0809219 | 0.848507902 | -0.237 |
| **732** | RAB1A, member RAS onco family OS=Canis lupus familiaris OX=9615 GN=RAB1A PE=4 SV=1 | A0A8I3PBQ1 | *RAB1A* | 22 kDa | 0.83 | 0.0809219 | 0.897510051 | -0.156 |
| **733** | 40S ribosomal protein S12 OS=Canis lupus familiaris OX=9615 GN=RPS12 PE=3 SV=1 | A0A8I3RTT6 | *RPS12* | 15 kDa | 0.83 | 0.0809219 | 0.897510051 | -0.156 |
| **734** | Calumenin OS=Canis lupus familiaris OX=9615 GN=CALU PE=4 SV=1 | A0A8I3S7P9 | *CALU* | 38 kDa | 0.83 | 0.0809219 | 0.908778116 | -0.138 |
| **735** | Phosphoglycerate mutase OS=Canis lupus familiaris OX=9615 GN=PGAM1 PE=3 SV=1 | A0A8I3SC19 | *PGAM1* | 29 kDa | 0.83 | 0.0809219 | 1.022570279 | 0.0322 |
| **736** | Small ribosomal subunit protein RACK1 OS=Canis lupus familiaris OX=9615 GN=RACK1 PE=3 SV=1 | A0A8I3MTL8 | *RACK1* | 9 kDa | 0.83 | 0.0809219 | 1.0604672 | 0.0847 |
| **737** | Ras suppressor protein 1 OS=Canis lupus familiaris OX=9615 GN=RSU1 PE=4 SV=1 | A0A8I3NS95 | *RSU1* | 29 kDa | 0.83 | 0.0809219 | 1.0604672 | 0.0847 |
| **738** | Peroxiredoxin 4 OS=Canis lupus familiaris OX=9615 GN=PRDX4 PE=4 SV=1 | A0A8I3QAR9 | *PRDX4* | 30 kDa | 0.84 | 0.0757207 | 0.469761375 | -1.09 |
| **739** | Exportin 1 OS=Canis lupus familiaris OX=9615 GN=XPO1 PE=3 SV=1 | A0A8I3NNH1 | *XPO1* | 123 kDa | 0.84 | 0.0757207 | 0.530343871 | -0.915 |

| | | | | | | | | |
|---|---|---|---|---|---|---|---|---|
| **740** | Proteasome subunit alpha type OS=Canis lupus familiaris OX=9615 GN=LOC119879604 PE=3 SV=1 | A0A8I3NQH6 | *LOC119879604* | 26 kDa | 0.84 | 0.0757207 | 0.729004689 | -0.456 |
| **741** | Eukaryotic translation initiation factor 3 subunit C OS=Canis lupus familiaris OX=9615 GN=LOC479795 PE=3 SV=1 | A0A8I3MQ25 | *LOC479795* | 106 kDa | 0.84 | 0.0757207 | 0.815637493 | -0.294 |
| **742** | Protein transport protein SEC23 OS=Canis lupus familiaris OX=9615 GN=SEC23A PE=3 SV=1 | A0A8I3N4T5 | *SEC23A* | 83 kDa | 0.84 | 0.0757207 | 0.873572896 | -0.195 |
| **743** | Uncharacterized protein OS=Canis lupus familiaris OX=9615 PE=3 SV=1 | A0A8I3PLT9 | | 28 kDa | 0.84 | 0.0757207 | 0.883927531 | -0.178 |
| **744** | Activator of HSP90 ATPase activity 1 OS=Canis lupus familiaris OX=9615 GN=AHSA1 PE=3 SV=1 | A0A8I3N859 | *AHSA1* | 38 kDa | 0.84 | 0.0757207 | 0.908778116 | -0.138 |
| **745** | Profilin OS=Canis lupus familiaris OX=9615 GN=PFN1 PE=3 SV=1 | A0A8I3MSY8 | *PFN1* | 15 kDa | 0.84 | 0.0757207 | 0.984661667 | -0.0223 |
| **746** | Uncharacterized protein OS=Canis lupus familiaris OX=9615 GN=MSN PE=4 SV=1 | A0A8I3Q023 | *MSN* | 68 kDa | 0.84 | 0.0757207 | 0.990686158 | -0.0135 |
| **747** | Insulin-like growth factor-binding protein 2 OS=Canis lupus familiaris OX=9615 GN=IGFBP2 PE=4 SV=1 | A0A8I3PX33 | *IGFBP2* | 34 kDa | 0.84 | 0.0757207 | 1.101141598 | 0.139 |
| **748** | HMA domain-containing protein OS=Canis lupus familiaris OX=9615 PE=4 SV=1 | A0A8I3PQ84 | | 8 kDa | 0.85 | 0.0705811 | 0.636397468 | -0.652 |
| **749** | Ribosomal protein S16 OS=Canis lupus familiaris OX=9615 GN=RPS16 PE=3 SV=1 | A0A8I3N9D0 | *RPS16* | 16 kDa | 0.85 | 0.0705811 | 0.795536484 | -0.33 |
| **750** | Coenzyme Q10B OS=Canis lupus familiaris OX=9615 GN=COQ10B PE=3 SV=1 | A0A8I3PT43 | *COQ10B* | 5 kDa | 0.85 | 0.0705811 | 0.848507902 | -0.237 |
| **751** | Amyloid-beta A4 protein OS=Canis lupus familiaris OX=9615 GN=APP PE=3 SV=1 | A0A8I3RZM5 | *APP* | 85 kDa | 0.85 | 0.0705811 | 1.007444246 | 0.0107 |
| **752** | Arp2/3 complex 34 kDa subunit OS=Canis lupus familiaris OX=9615 GN=ARPC2 PE=3 SV=1 | A0A8I3PPQ5 | *ARPC2* | 38 kDa | 0.85 | 0.0705811 | 1.0604672 | 0.0847 |
| **753** | 60S ribosomal protein L36a OS=Canis lupus familiaris OX=9615 GN=LOC119868317 PE=3 SV=1 | A0A8I3P5R4 | *LOC119868317* | 16 kDa | 0.85 | 0.0705811 | 1.0604672 | 0.0847 |
| **754** | CD248 molecule OS=Canis lupus familiaris OX=9615 GN=CD248 PE=4 SV=1 | A0A8I3NR22 | *CD248* | 83 kDa | 0.85 | 0.0705811 | 1.108800644 | 0.149 |
| **755** | Superoxide dismutase [Cu-Zn] OS=Canis lupus familiaris OX=9615 GN=SOD3 PE=3 SV=1 | A0A8I3MW81 | *SOD3* | 28 kDa | 0.85 | 0.0705811 | 1.458009379 | 0.544 |
| **756** | Protein-L-isoaspartate O-methyltransferase OS=Canis lupus familiaris OX=9615 GN=PCMT1 PE=3 SV=1 | A0A8I3S3U0 | *PCMT1* | 29 kDa | 0.85 | 0.0705811 | 1.697015803 | 0.763 |
| **757** | Small ribosomal subunit protein uS4 OS=Canis lupus familiaris OX=9615 GN=RPS9 PE=3 SV=1 | A0A8I3RQP7 | *RPS9* | 17 kDa | 0.86 | 0.0655015 | 0.777546036 | -0.363 |
| **758** | Uncharacterized protein OS=Canis lupus familiaris OX=9615 PE=3 SV=1 | A0A8I3RWN5 | | 18 kDa | 0.86 | 0.0655015 | 0.883927531 | -0.178 |
| **759** | Gelsolin OS=Canis lupus familiaris OX=9615 GN=GSN PE=3 SV=1 | A0A8I3P8B2 | *GSN* | 85 kDa | 0.86 | 0.0655015 | 0.894404902 | -0.161 |
| **760** | Fibulin 2 OS=Canis lupus familiaris OX=9615 GN=FBLN2 PE=3 SV=1 | A0A8I3S3J1 | *FBLN2* | 126 kDa | 0.86 | 0.0655015 | 0.920187651 | -0.12 |
| **761** | Uncharacterized protein OS=Canis lupus familiaris OX=9615 GN=AHCY PE=3 SV=1 | A0A8I3NY33 | *AHCY* | 48 kDa | 0.86 | 0.0655015 | 0.927873476 | -0.108 |
| **762** | Eukaryotic translation initiation factor 3 subunit E OS=Canis lupus familiaris OX=9615 GN=EIF3E PE=3 SV=1 | A0A8I3NHG8 | *EIF3E* | 52 kDa | 0.86 | 0.0655015 | 0.94258851 | -0.0853 |
| **763** | Actin-related protein 2/3 complex subunit 3 OS=Canis lupus familiaris OX=9615 PE=3 SV=1 | A0A8I3N5B8 | | 18 kDa | 0.86 | 0.0655015 | 0.954422527 | -0.0673 |

| | | | | | | | | |
|---|---|---|---|---|---|---|---|---|
| **764** | S-adenosyl-L-homocysteine hydrolase NAD binding domain-containing protein OS=Canis lupus familiaris OX=9615 PE=3 SV=1 | A0A8I3NL75 | | 33 kDa | 0.86 | 0.0655015 | 0.954422527 | -0.0673 |
| **765** | Purine nucleoside phosphorylase OS=Canis lupus familiaris OX=9615 GN=PNP PE=3 SV=1 | A0A8I3PKI7 | *PNP* | 32 kDa | 0.86 | 0.0655015 | 0.968215436 | -0.0466 |
| **766** | Uncharacterized protein OS=Canis lupus familiaris OX=9615 GN=PPIA PE=4 SV=1 | A0A8I3N9K7 | *PPIA* | 33 kDa | 0.86 | 0.0655015 | 0.968215436 | -0.0466 |
| **767** | ST13 Hsp70 interacting protein OS=Canis lupus familiaris OX=9615 GN=ST13 PE=4 SV=1 | A0A8I3PA49 | *ST13* | 38 kDa | 0.86 | 0.0655015 | 0.994160026 | -0.00845 |
| **768** | Proteasome 26S subunit, non-ATPase 6 OS=Canis lupus familiaris OX=9615 GN=PSMD6 PE=4 SV=1 | A0A8I3RXK2 | *PSMD6* | 42 kDa | 0.86 | 0.0655015 | 1.0604672 | 0.0847 |
| **769** | Rab GDP dissociation inhibitor OS=Canis lupus familiaris OX=9615 PE=3 SV=1 | A0A8I3PPL6 | | 42 kDa | 0.86 | 0.0655015 | 1.0604672 | 0.0847 |
| **770** | T-complex protein 1 subunit theta OS=Canis lupus familiaris OX=9615 GN=CCT8 PE=3 SV=1 | A0A8I3P3D6 | *CCT8* | 52 kDa | 0.86 | 0.0655015 | 1.142346247 | 0.192 |
| **771** | Cysteine rich protein 1 OS=Canis lupus familiaris OX=9615 GN=CRIP1 PE=4 SV=1 | A0A8I3PT20 | *CRIP1* | 9 kDa | 0.86 | 0.0655015 | 1.378405153 | 0.463 |
| **772** | C1q and TNF related 5 OS=Canis lupus familiaris OX=9615 GN=C1QTNF5 PE=4 SV=1 | A0A8I3NXU5 | *C1QTNF5* | 25 kDa | 0.87 | 0.0604807 | 0.530343871 | -0.915 |
| **773** | Ribosomal protein S5 OS=Canis lupus familiaris OX=9615 GN=RPS5 PE=3 SV=1 | A0A8I3RQY4 | *RPS5* | 27 kDa | 0.87 | 0.0604807 | 0.848507902 | -0.237 |
| **774** | Uncharacterized protein OS=Canis lupus familiaris OX=9615 GN=H1-5 PE=3 SV=1 | A0A8I3P6L4 | *H1-5* | 23 kDa | 0.87 | 0.0604807 | 0.90000193 | -0.152 |
| **775** | Fibrillin 1 OS=Canis lupus familiaris OX=9615 GN=FBN1 PE=3 SV=1 | A0A8I3PI20 | *FBN1* | 312 kDa | 0.87 | 0.0604807 | 0.904379378 | -0.145 |
| **776** | Laminin subunit beta 1 OS=Canis lupus familiaris OX=9615 GN=LAMB1 PE=4 SV=1 | A0A8I3PKU7 | *LAMB1* | 197 kDa | 0.87 | 0.0604807 | 0.926588062 | -0.11 |
| **777** | T-complex protein 1 subunit beta OS=Canis lupus familiaris OX=9615 GN=CCT2 PE=3 SV=1 | A0A8I3S011 | *CCT2* | 53 kDa | 0.87 | 0.0604807 | 0.927873476 | -0.108 |
| **778** | Elongation factor 1-alpha OS=Canis lupus familiaris OX=9615 PE=3 SV=1 | A0A8I3S0N5 | | 50 kDa | 0.87 | 0.0604807 | 0.994160026 | -0.00845 |
| **779** | Lactate dehydrogenase C OS=Canis lupus familiaris OX=9615 PE=3 SV=1 | A0A8I3Q8U0 | | 28 kDa | 0.87 | 0.0604807 | 1.091263877 | 0.126 |
| **780** | Septin OS=Canis lupus familiaris OX=9615 GN=SEPTIN11 PE=3 SV=1 | A0A8I3NZJ3 | *SEPTIN11* | 50 kDa | 0.88 | 0.0555173 | 0.636397468 | -0.652 |
| **781** | Cathepsin B OS=Canis lupus familiaris OX=9615 GN=CTSB PE=3 SV=1 | A0A8I3S0M3 | *CTSB* | 38 kDa | 0.88 | 0.0555173 | 0.774855931 | -0.368 |
| **782** | Isochorismatase domain containing 1 OS=Canis lupus familiaris OX=9615 GN=ISOC1 PE=3 SV=1 | A0A8I3RV78 | *ISOC1* | 33 kDa | 0.88 | 0.0555173 | 0.824733549 | -0.278 |
| **783** | Chordin like 1 OS=Canis lupus familiaris OX=9615 GN=CHRDL1 PE=4 SV=1 | A0A8I3PGT6 | *CHRDL1* | 52 kDa | 0.88 | 0.0555173 | 0.873572896 | -0.195 |
| **784** | Uncharacterized protein OS=Canis lupus familiaris OX=9615 GN=SERPINB6 PE=3 SV=1 | A0A8I3P230 | *SERPINB6* | 42 kDa | 0.88 | 0.0555173 | 0.964063446 | -0.0528 |
| **785** | Brain abundant membrane attached signal protein 1 OS=Canis lupus familiaris OX=9615 PE=3 SV=1 | A0A8I3MP32 | | 27 kDa | 0.88 | 0.0555173 | 1.0604672 | 0.0847 |
| **786** | REVERSE_A0A8I3PCF7 | REVERSE_A0A8I3PCF7 | | | 0.88 | 0.0555173 | 1.0604672 | 0.0847 |
| **787** | Proteasome 20S subunit alpha 2 OS=Canis lupus familiaris OX=9615 GN=PSMA2 PE=3 SV=1 | A0A8I3NUD7 | *PSMA2* | 19 kDa | 0.88 | 0.0555173 | 1.0604672 | 0.0847 |

| | | | | | | | | |
|---|---|---|---|---|---|---|---|---|
| **788** | Laminin subunit gamma 1 OS=Canis lupus familiaris OX=9615 GN=LAMC1 PE=4 SV=1 | A0A8I3MTJ9 | *LAMC1* | 178 kDa | 0.88 | 0.0555173 | 1.0604672 | 0.0847 |
| **789** | Serine/threonine-protein phosphatase 2A 55 kDa regulatory subunit B OS=Canis lupus familiaris OX=9615 GN=PPP2R2A PE=3 SV=1 | A0A8I3RXQ1 | *PPP2R2A* | 60 kDa | 0.88 | 0.0555173 | 1.0604672 | 0.0847 |
| **790** | CDV3 homolog OS=Canis lupus familiaris OX=9615 GN=CDV3 PE=3 SV=1 | A0A8I3S7B6 | *CDV3* | 27 kDa | 0.88 | 0.0555173 | 1.32592576 | 0.407 |
| **791** | Heterogeneous nuclear ribonucleoprotein L OS=Canis lupus familiaris OX=9615 GN=HNRNPL PE=4 SV=1 | A0A8I3MVT0 | *HNRNPL* | 64 kDa | 0.88 | 0.0555173 | 1.591072968 | 0.67 |
| **792** | DExD-box helicase 39B OS=Canis lupus familiaris OX=9615 GN=DDX39B PE=4 SV=1 | A0A8I3NE94 | *DDX39B* | 49 kDa | 0.89 | 0.05061 | 0.453759578 | -1.14 |
| **793** | 26S proteasome non-ATPase regulatory subunit 4 OS=Canis lupus familiaris OX=9615 GN=PSMD4 PE=3 SV=1 | A0A8I3PB85 | *PSMD4* | 42 kDa | 0.89 | 0.05061 | 0.530343871 | -0.915 |
| **794** | H(+)-transporting two-sector ATPase OS=Canis lupus familiaris OX=9615 GN=ATP6V1A PE=3 SV=1 | A0A8I3Q434 | *ATP6V1A* | 65 kDa | 0.89 | 0.05061 | 0.636397468 | -0.652 |
| **795** | RAP1B, member of RAS oncogene family OS=Canis lupus familiaris OX=9615 GN=RAP1B PE=4 SV=1 | A0A8I3NP25 | *RAP1B* | 19 kDa | 0.89 | 0.05061 | 0.848507902 | -0.237 |
| **796** | Annexin A2 OS=Canis lupus familiaris OX=9615 GN=ANXA2 PE=1 SV=1 | Q6TEQ7 | *ANXA2* | 39 kDa | 0.89 | 0.05061 | 0.962260891 | -0.0555 |
| **797** | Tyrosine 3-monooxygenase/tryptophan 5-monooxygenase activation protein eta OS=Canis lupus familiaris OX=9615 GN=YWHAH PE=3 SV=1 | A0A8I3S659 | *YWHAH* | 28 kDa | 0.89 | 0.05061 | 0.972048363 | -0.0409 |
| **798** | Peroxiredoxin 1 OS=Canis lupus familiaris OX=9615 GN=PRDX1 PE=4 SV=1 | A0A8I3S3A0 | *PRDX1* | 31 kDa | 0.89 | 0.05061 | 0.986027644 | -0.0203 |
| **799** | U1 small nuclear ribonucleoprotein A OS=Canis lupus familiaris OX=9615 GN=SNRPA PE=3 SV=1 | A0A8I3MNF1 | *SNRPA* | 31 kDa | 0.89 | 0.05061 | 1.0604672 | 0.0847 |
| **800** | Small ribosomal subunit protein uS13 OS=Canis lupus familiaris OX=9615 GN=RPS18 PE=1 SV=3 | Q5TJE9 | *RPS18* | 18 kDa | 0.89 | 0.05061 | 1.0604672 | 0.0847 |
| **801** | Uncharacterized protein OS=Canis lupus familiaris OX=9615 PE=3 SV=1 | A0A8I3S7N3 | | 18 kDa | 0.89 | 0.05061 | 1.32592576 | 0.407 |
| **802** | Nucleosome assembly protein 1 like 4 OS=Canis lupus familiaris OX=9615 GN=NAP1L4 PE=3 SV=1 | A0A8I3NFR7 | *NAP1L4* | 44 kDa | 0.9 | 0.0457575 | 0.423372656 | -1.24 |
| **803** | Ribosomal protein S10 OS=Canis lupus familiaris OX=9615 GN=RPS10 PE=3 SV=1 | A0A8I3PQS5 | *RPS10* | 19 kDa | 0.9 | 0.0457575 | 0.530343871 | -0.915 |
| **804** | Heterogeneous nuclear ribonucleoprotein K OS=Canis lupus familiaris OX=9615 GN=HNRNPK PE=4 SV=1 | A0A8I3MXC5 | *HNRNPK* | 51 kDa | 0.9 | 0.0457575 | 0.636397468 | -0.652 |
| **805** | S-adenosylmethionine synthase OS=Canis lupus familiaris OX=9615 GN=MAT2A PE=3 SV=1 | A0A8I3NU09 | *MAT2A* | 44 kDa | 0.9 | 0.0457575 | 0.707106781 | -0.5 |
| **806** | Actin related protein 1A OS=Canis lupus familiaris OX=9615 GN=ACTR1A PE=3 SV=1 | A0A8I3S4Z6 | *ACTR1A* | 45 kDa | 0.9 | 0.0457575 | 0.707106781 | -0.5 |
| **807** | Tyrosine 3-monooxygenase/tryptophan 5-monooxygenase activation protein gamma OS=Canis lupus familiaris OX=9615 GN=YWHAG PE=3 SV=1 | A0A8I3MT06 | *YWHAG* | 28 kDa | 0.9 | 0.0457575 | 0.707106781 | -0.5 |
| **808** | Actin alpha 1, skeletal muscle OS=Canis lupus familiaris OX=9615 GN=ACTA1 PE=3 SV=1 | A0A8I3MHG9 | *ACTA1* | 46 kDa | 0.9 | 0.0457575 | 0.720964436 | -0.472 |
| **809** | serine--tRNA ligase OS=Canis lupus familiaris OX=9615 GN=SARS1 PE=3 SV=1 | A0A8I3MRY7 | *SARS1* | 61 kDa | 0.9 | 0.0457575 | 0.742261785 | -0.43 |
| **810** | Platelet-activating factor acetylhydrolase OS=Canis lupus familiaris OX=9615 GN=PLA2G7 PE=2 SV=1 | Q28262 | *PLA2G7* | 50 kDa | 0.9 | 0.0457575 | 0.795536484 | -0.33 |

| | | | | | | | | |
|---|---|---|---|---|---|---|---|---|
| **811** | Vacuolar protein sorting-associated protein 35 OS=Canis lupus familiaris OX=9615 GN=VPS35 PE=3 SV=1 | A0A8I3NTA9 | *VPS35* | 83 kDa | 0.9 | 0.0457575 | 0.86154616 | -0.215 |
| **812** | Beta-hexosaminidase OS=Canis lupus familiaris OX=9615 GN=HEXB PE=3 SV=1 | A0A8I3MY15 | *HEXB* | 59 kDa | 0.9 | 0.0457575 | 0.867538687 | -0.205 |
| **813** | Collagen type VI alpha 1 chain OS=Canis lupus familiaris OX=9615 GN=COL6A1 PE=4 SV=1 | A0A8I3S7Z3 | *COL6A1* | 109 kDa | 0.9 | 0.0457575 | 0.962127503 | -0.0557 |
| **814** | Poly(A) binding protein cytoplasmic 1 like OS=Canis lupus familiaris OX=9615 GN=PABPC1L PE=3 SV=1 | A0A8I3NSL5 | *PABPC1L* | 30 kDa | 0.9 | 0.0457575 | 1.001519145 | 0.00219 |
| **815** | Collagen type XV alpha 1 chain OS=Canis lupus familiaris OX=9615 GN=COL15A1 PE=4 SV=1 | A0A8I3N1V6 | *COL15A1* | 140 kDa | 0.9 | 0.0457575 | 1.0604672 | 0.0847 |
| **816** | Tropomyosin 3 OS=Canis lupus familiaris OX=9615 GN=TPM3 PE=3 SV=1 | A0A8I3NRI7 | *TPM3* | 31 kDa | 0.9 | 0.0457575 | 1.087488391 | 0.121 |
| **817** | Histone H2A OS=Canis lupus familiaris OX=9615 PE=3 SV=1 | A0A8I3NZV6 | | 20 kDa | 0.9 | 0.0457575 | 1.119612889 | 0.163 |
| **818** | Insulin like growth factor binding protein 6 OS=Canis lupus familiaris OX=9615 GN=IGFBP6 PE=4 SV=1 | A0A8I3PEI1 | *IGFBP6* | 28 kDa | 0.9 | 0.0457575 | 1.148698355 | 0.2 |
| **819** | ADP-ribosylation factor OS=Canis lupus familiaris OX=9615 GN=ARF3 PE=3 SV=1 | A0A8I3PDW2 | *ARF3* | 20 kDa | 0.9 | 0.0457575 | 1.414213562 | 0.5 |
| **820** | Aspartate--tRNA ligase, cytoplasmic OS=Canis lupus familiaris OX=9615 GN=DARS1 PE=3 SV=1 | A0A8I3NDL0 | *DARS1* | 55 kDa | 0.91 | 0.0409586 | 0.742261785 | -0.43 |
| **821** | Uncharacterized protein OS=Canis lupus familiaris OX=9615 PE=3 SV=1 | A0A8I3Q483 | | 15 kDa | 0.91 | 0.0409586 | 0.795536484 | -0.33 |
| **822** | Small ribosomal subunit protein eS28 OS=Canis lupus familiaris OX=9615 GN=RPS28 PE=3 SV=1 | A0A8I3NCS3 | *RPS28* | 8 kDa | 0.91 | 0.0409586 | 0.824733549 | -0.278 |
| **823** | ADF-H domain-containing protein OS=Canis lupus familiaris OX=9615 PE=3 SV=1 | A0A8I3NVG1 | | 17 kDa | 0.91 | 0.0409586 | 0.867538687 | -0.205 |
| **824** | ADF-H domain-containing protein OS=Canis lupus familiaris OX=9615 GN=DSTN PE=3 SV=1 | A0A8I3S0S0 | *DSTN* | 40 kDa | 0.91 | 0.0409586 | 0.908778116 | -0.138 |
| **825** | 60S ribosomal protein L13 OS=Canis lupus familiaris OX=9615 GN=RPL13 PE=3 SV=1 | A0A8I3MMQ3 | *RPL13* | 19 kDa | 0.91 | 0.0409586 | 0.908778116 | -0.138 |
| **826** | Tubulin alpha chain OS=Canis lupus familiaris OX=9615 GN=TUBA1C PE=3 SV=1 | A0A8I3P9E1 | *TUBA1C* | 54 kDa | 0.91 | 0.0409586 | 1.018044059 | 0.0258 |
| **827** | Calsyntenin 1 OS=Canis lupus familiaris OX=9615 GN=LOC479600 PE=3 SV=1 | A0A8I3Q599 | *LOC479600* | 108 kDa | 0.91 | 0.0409586 | 1.0604672 | 0.0847 |
| **828** | Proteasome subunit alpha type OS=Canis lupus familiaris OX=9615 GN=PSMA6 PE=3 SV=1 | A0A8I3MET1 | *PSMA6* | 23 kDa | 0.92 | 0.0362122 | 0.795536484 | -0.33 |
| **829** | Uncharacterized protein OS=Canis lupus familiaris OX=9615 GN=AK2 PE=3 SV=1 | A0A8I3MNC5 | *AK2* | 18 kDa | 0.92 | 0.0362122 | 0.795536484 | -0.33 |
| **830** | Stathmin OS=Canis lupus familiaris OX=9615 GN=STMN1 PE=3 SV=1 | A0A8I3P045 | *STMN1* | 17 kDa | 0.92 | 0.0362122 | 0.815637493 | -0.294 |
| **831** | Glucosidase II alpha subunit OS=Canis lupus familiaris OX=9615 GN=GANAB PE=3 SV=1 | A0A8I3NM74 | *GANAB* | 109 kDa | 0.92 | 0.0362122 | 0.94258851 | -0.0853 |
| **832** | Galectin 3 OS=Canis lupus familiaris OX=9615 GN=LGALS3 PE=4 SV=1 | A0A8I3NWF7 | *LGALS3* | 31 kDa | 0.92 | 0.0362122 | 0.945467649 | -0.0809 |
| **833** | Lactate/malate dehydrogenase C-terminal domain-containing protein OS=Canis lupus familiaris OX=9615 PE=4 SV=1 | A0A8I3NU98 | | 7 kDa | 0.92 | 0.0362122 | 0.964063446 | -0.0528 |
| **834** | Nucleophosmin OS=Canis lupus familiaris OX=9615 PE=4 SV=1 | A0A8I3PQH9 | | 19 kDa | 0.92 | 0.0362122 | 0.989725257 | -0.0149 |

| | | | | | | | | |
|---|---|---|---|---|---|---|---|---|
| **835** | Tyrosine 3-monooxygenase/tryptophan 5-monooxygenase activation protein zeta OS=Canis lupus familiaris OX=9615 GN=YWHAZ PE=3 SV=1 | A0A8I3Q214 | *YWHAZ* | 28 kDa | 0.92 | 0.0362122 | 0.991991731 | -0.0116 |
| **836** | Pyruvate kinase OS=Canis lupus familiaris OX=9615 GN=PKM PE=3 SV=1 | A0A8I3PPF8 | *PKM* | 58 kDa | 0.92 | 0.0362122 | 1.04181564 | 0.0591 |
| **837** | Protein disulfide-isomerase OS=Canis lupus familiaris OX=9615 GN=PDIA3 PE=3 SV=1 | A0A8I3S5M8 | *PDIA3* | 57 kDa | 0.92 | 0.0362122 | 1.0604672 | 0.0847 |
| **838** | Peroxiredoxin 2 OS=Canis lupus familiaris OX=9615 GN=PRDX2 PE=3 SV=1 | A0A8I3NZ15 | *PRDX2* | 22 kDa | 0.92 | 0.0362122 | 1.095811766 | 0.132 |
| **839** | EH domain containing 1 OS=Canis lupus familiaris OX=9615 GN=EHD1 PE=4 SV=1 | A0A8I3Q4C4 | *EHD1* | 69 kDa | 0.92 | 0.0362122 | 1.193335743 | 0.255 |
| **840** | Splicing factor 1 OS=Canis lupus familiaris OX=9615 GN=SF1 PE=3 SV=1 | A0A8I3Q776 | *SF1* | 68 kDa | 0.93 | 0.0315171 | 0.453759578 | -1.14 |
| **841** | Uncharacterized protein OS=Canis lupus familiaris OX=9615 GN=PAICS PE=3 SV=1 | A0A8I3RXP6 | *PAICS* | 50 kDa | 0.93 | 0.0315171 | 0.795536484 | -0.33 |
| **842** | Actinin alpha 4 OS=Canis lupus familiaris OX=9615 GN=LGALS7B PE=3 SV=1 | A0A8I3MWJ9 | *LGALS7B* | 103 kDa | 0.93 | 0.0315171 | 0.795536484 | -0.33 |
| **843** | 40S ribosomal protein S4 OS=Canis lupus familiaris OX=9615 GN=RPS4X PE=3 SV=1 | A0A8I3PBZ1 | *RPS4X* | 30 kDa | 0.93 | 0.0315171 | 0.824733549 | -0.278 |
| **844** | Matrix metallopeptidase 2 OS=Canis lupus familiaris OX=9615 GN=MMP2 PE=3 SV=1 | A0A8I3MD29 | *MMP2* | 69 kDa | 0.93 | 0.0315171 | 0.833353207 | -0.263 |
| **845** | Cystatin B OS=Canis lupus familiaris OX=9615 GN=CSTB PE=3 SV=1 | A0A8I3NW54 | *CSTB* | 11 kDa | 0.93 | 0.0315171 | 0.925304428 | -0.112 |
| **846** | 14-3-3 domain-containing protein OS=Canis lupus familiaris OX=9615 GN=YWHAE PE=3 SV=1 | A0A8I3S0K8 | *YWHAE* | 25 kDa | 0.93 | 0.0315171 | 1.0604672 | 0.0847 |
| **847** | threonine--tRNA ligase OS=Canis lupus familiaris OX=9615 GN=TARS1 PE=3 SV=1 | A0A8I3MZD1 | *TARS1* | 84 kDa | 0.93 | 0.0315171 | 1.0604672 | 0.0847 |
| **848** | Histidine--tRNA ligase, cytoplasmic OS=Canis lupus familiaris OX=9615 GN=HARS1 PE=4 SV=1 | A0A8I3RQK9 | *HARS1* | 53 kDa | 0.93 | 0.0315171 | 1.0604672 | 0.0847 |
| **849** | Peroxiredoxin-6 OS=Canis lupus familiaris OX=9615 GN=PRDX6 PE=3 SV=1 | A0A8I3NWQ3 | *PRDX6* | 25 kDa | 0.93 | 0.0315171 | 1.118061851 | 0.161 |
| **850** | Catenin alpha 1 OS=Canis lupus familiaris OX=9615 GN=CTNNA1 PE=3 SV=1 | A0A8I3PEF2 | *CTNNA1* | 92 kDa | 0.93 | 0.0315171 | 1.484523571 | 0.57 |
| **851** | Procathepsin L OS=Canis lupus familiaris OX=9615 GN=CTSL PE=2 SV=1 | Q9GL24 | *CTSL* | 37 kDa | 0.94 | 0.0268721 | 0.795536484 | -0.33 |
| **852** | Coatomer subunit alpha OS=Canis lupus familiaris OX=9615 GN=COPA PE=4 SV=1 | A0A8I3Q9Z2 | *COPA* | 138 kDa | 0.94 | 0.0268721 | 0.802737389 | -0.317 |
| **853** | Glucose-6-phosphate 1-dehydrogenase OS=Canis lupus familiaris OX=9615 GN=G6PD PE=3 SV=1 | A0A8I3Q8F7 | *G6PD* | 68 kDa | 0.94 | 0.0268721 | 0.803293997 | -0.316 |
| **854** | Malate dehydrogenase 1 OS=Canis lupus familiaris OX=9615 GN=MDH1 PE=3 SV=1 | A0A8I3P822 | *MDH1* | 39 kDa | 0.94 | 0.0268721 | 0.815637493 | -0.294 |
| **855** | Uncharacterized protein OS=Canis lupus familiaris OX=9615 PE=3 SV=1 | A0A8I3MTG8 | | 6 kDa | 0.94 | 0.0268721 | 0.848507902 | -0.237 |
| **856** | Proteasome subunit beta OS=Canis lupus familiaris OX=9615 GN=PSMB3 PE=3 SV=1 | A0A8I3RXV3 | *PSMB3* | 23 kDa | 0.94 | 0.0268721 | 0.848507902 | -0.237 |
| **857** | Small ribosomal subunit protein eS1 OS=Canis lupus familiaris OX=9615 GN=RPS3A PE=3 SV=1 | A0A8I3SAX6 | *RPS3A* | 30 kDa | 0.94 | 0.0268721 | 0.897510051 | -0.156 |
| **858** | Thrombospondin 2 OS=Canis lupus familiaris OX=9615 GN=THBS2 PE=2 SV=1 | D5IGC9 | *THBS2* | 129 kDa | 0.94 | 0.0268721 | 0.938741601 | -0.0912 |

| | | | | | | | | |
|---|---|---|---|---|---|---|---|---|
| **859** | 78 kDa glucose-regulated protein OS=Canis lupus familiaris OX=9615 GN=HSPA5 PE=3 SV=1 | A0A8I3NCJ5 | *HSPA5* | 72 kDa | 0.94 | 0.0268721 | 1.012695187 | 0.0182 |
| **860** | Clathrin heavy chain OS=Canis lupus familiaris OX=9615 GN=CLTCL1 PE=3 SV=1 | A0A8I3Q303 | *CLTCL1* | 180 kDa | 0.94 | 0.0268721 | 1.0604672 | 0.0847 |
| **861** | Vesicular integral-membrane protein VIP36 OS=Canis lupus familiaris OX=9615 GN=LMAN2 PE=1 SV=1 | P49256 | *LMAN2* | 40 kDa | 0.94 | 0.0268721 | 1.414213562 | 0.5 |
| **862** | DNA damage-binding protein 1 OS=Canis lupus familiaris OX=9615 GN=DDB1 PE=3 SV=1 | A0A8I3RZS2 | *DDB1* | 121 kDa | 0.95 | 0.0222764 | 0.496546248 | -1.01 |
| **863** | Golgi membrane protein 1 OS=Canis lupus familiaris OX=9615 GN=GOLM1 PE=3 SV=1 | A0A8I3MVA9 | *GOLM1* | 47 kDa | 0.95 | 0.0222764 | 0.837406488 | -0.256 |
| **864** | Histidine--tRNA ligase, cytoplasmic OS=Canis lupus familiaris OX=9615 GN=HARS1 PE=3 SV=1 | A0A8I3MMJ2 | *HARS1* | 44 kDa | 0.95 | 0.0222764 | 0.848507902 | -0.237 |
| **865** | Integrin beta OS=Canis lupus familiaris OX=9615 GN=ITGB1 PE=3 SV=1 | A0A8I3MMP8 | *ITGB1* | 89 kDa | 0.95 | 0.0222764 | 0.927873476 | -0.108 |
| **866** | Zyxin OS=Canis lupus familiaris OX=9615 GN=ZYX PE=4 SV=1 | A0A8I3P876 | *ZYX* | 63 kDa | 0.95 | 0.0222764 | 0.957802429 | -0.0622 |
| **867** | Reticulocalbin 1 OS=Canis lupus familiaris OX=9615 GN=RCN1 PE=4 SV=1 | A0A8I3Q2T3 | *RCN1* | 45 kDa | 0.95 | 0.0222764 | 1.0604672 | 0.0847 |
| **868** | Arginine--tRNA ligase, cytoplasmic OS=Canis lupus familiaris OX=9615 GN=RARS1 PE=3 SV=1 | A0A8I3MH91 | *RARS1* | 74 kDa | 0.95 | 0.0222764 | 1.0604672 | 0.0847 |
| **869** | U1 small nuclear ribonucleoprotein 70 kDa OS=Canis lupus familiaris OX=9615 GN=SNRNP70 PE=4 SV=1 | A0A8I3MF21 | *SNRNP70* | 52 kDa | 0.96 | 0.0177288 | 0.707106781 | -0.5 |
| **870** | Complement C3 OS=Canis lupus familiaris OX=9615 GN=C3 PE=4 SV=1 | A0A8I3NQA0 | *C3* | 192 kDa | 0.96 | 0.0177288 | 0.749499801 | -0.416 |
| **871** | Phosphoglucomutase 1 OS=Canis lupus familiaris OX=9615 GN=PGM1 PE=3 SV=1 | A0A8I3MMJ8 | *PGM1* | 60 kDa | 0.96 | 0.0177288 | 0.848507902 | -0.237 |
| **872** | Calpastatin OS=Canis lupus familiaris OX=9615 GN=CAST PE=3 SV=1 | A0A8I3MIR1 | *CAST* | 76 kDa | 0.96 | 0.0177288 | 0.86154616 | -0.215 |
| **873** | Uncharacterized protein OS=Canis lupus familiaris OX=9615 GN=FABP3 PE=3 SV=1 | A0A8I3N3X1 | *FABP3* | 15 kDa | 0.96 | 0.0177288 | 0.959463613 | -0.0597 |
| **874** | Transforming growth factor beta induced OS=Canis lupus familiaris OX=9615 GN=TGFBI PE=4 SV=1 | A0A8I3P2C5 | *TGFBI* | 89 kDa | 0.96 | 0.0177288 | 1.004620069 | 0.00665 |
| **875** | 60S ribosomal protein L13 OS=Canis lupus familiaris OX=9615 GN=RPL13 PE=3 SV=1 | A0A8I3MST1 | *RPL13* | 24 kDa | 0.96 | 0.0177288 | 1.0604672 | 0.0847 |
| **876** | Nuclear transport factor 2 OS=Canis lupus familiaris OX=9615 GN=NUTF2 PE=4 SV=1 | A0A8I3N375 | *NUTF2* | 14 kDa | 0.96 | 0.0177288 | 1.0604672 | 0.0847 |
| **877** | Cullin associated and neddylation dissociated 1 OS=Canis lupus familiaris OX=9615 GN=CAND1 PE=3 SV=1 | A0A8I3NT24 | *CAND1* | 136 kDa | 0.97 | 0.0132283 | 0.652477474 | -0.616 |
| **878** | Complement C1s OS=Canis lupus familiaris OX=9615 GN=C1S PE=4 SV=1 | A0A8I3PUL5 | *C1S* | 78 kDa | 0.97 | 0.0132283 | 0.671751713 | -0.574 |
| **879** | GTP-binding nuclear protein Ran OS=Canis lupus familiaris OX=9615 GN=RAN PE=3 SV=1 | A0A8I3Q1D6 | *RAN* | 51 kDa | 0.97 | 0.0132283 | 0.815637493 | -0.294 |
| **880** | Eukaryotic translation initiation factor 3 subunit A OS=Canis lupus familiaris OX=9615 GN=EIF3A PE=3 SV=1 | A0A8I3NXD1 | *EIF3A* | 163 kDa | 0.97 | 0.0132283 | 0.858565436 | -0.22 |
| **881** | Ribosomal protein L18 OS=Canis lupus familiaris OX=9615 GN=RPL18 PE=3 SV=1 | A0A8I3MGA2 | *RPL18* | 18 kDa | 0.97 | 0.0132283 | 0.858565436 | -0.22 |
| **882** | Myosin light chain 6B OS=Canis lupus familiaris OX=9615 GN=MYL6 PE=4 SV=1 | A0A8I3RUW5 | *MYL6* | 18 kDa | 0.97 | 0.0132283 | 1.0604672 | 0.0847 |

| | | | | | | | | |
|---|---|---|---|---|---|---|---|---|
| **883** | Carnosine dipeptidase 2 OS=Canis lupus familiaris OX=9615 GN=CNDP2 PE=3 SV=1 | A0A8I3MEU4 | *CNDP2* | 53 kDa | 0.97 | 0.0132283 | 1.0604672 | 0.0847 |
| **884** | Importin 5 OS=Canis lupus familiaris OX=9615 GN=IPO5 PE=4 SV=1 | A0A8I3PAU2 | *IPO5* | 126 kDa | 0.97 | 0.0132283 | 1.0604672 | 0.0847 |
| **885** | Tropomyosin 2 OS=Canis lupus familiaris OX=9615 GN=TPM2 PE=3 SV=1 | A0A8I3N770 | *TPM2* | 32 kDa | 0.97 | 0.0132283 | 1.086734863 | 0.12 |
| **886** | Calponin OS=Canis lupus familiaris OX=9615 GN=CNN1 PE=3 SV=1 | A0A8I3S0C2 | *CNN1* | 35 kDa | 0.98 | 0.0087739 | 0.530343871 | -0.915 |
| **887** | Small RNA binding exonuclease protection factor La OS=Canis lupus familiaris OX=9615 GN=SSB PE=4 SV=1 | A0A8I3PZQ3 | *SSB* | 47 kDa | 0.98 | 0.0087739 | 0.530343871 | -0.915 |
| **888** | Inosine-5'-monophosphate dehydrogenase OS=Canis lupus familiaris OX=9615 GN=IMPDH2 PE=3 SV=1 | A0A8I3Q7R1 | *IMPDH2* | 56 kDa | 0.98 | 0.0087739 | 0.795536484 | -0.33 |
| **889** | Transaldolase OS=Canis lupus familiaris OX=9615 GN=TALDO1 PE=3 SV=1 | A0A8I3P237 | *TALDO1* | 39 kDa | 0.98 | 0.0087739 | 0.901250463 | -0.15 |
| **890** | Laminin subunit alpha 4 OS=Canis lupus familiaris OX=9615 GN=LAMA4 PE=4 SV=1 | A0A8I3PFE5 | *LAMA4* | 199 kDa | 0.98 | 0.0087739 | 1.0604672 | 0.0847 |
| **891** | EGF like repeats and discoidin domains 3 OS=Canis lupus familiaris OX=9615 GN=EDIL3 PE=4 SV=1 | A0A8I3P5P2 | *EDIL3* | 53 kDa | 0.98 | 0.0087739 | 1.295940965 | 0.374 |
| **892** | Hsp90 co-chaperone Cdc37 OS=Canis lupus familiaris OX=9615 GN=CDC37 PE=3 SV=1 | A0A8I3PXI0 | *CDC37* | 45 kDa | 0.98 | 0.0087739 | 1.32592576 | 0.407 |
| **893** | Coatomer subunit beta OS=Canis lupus familiaris OX=9615 GN=COPB1 PE=4 SV=1 | A0A8I3PHH7 | *COPB1* | 102 kDa | 0.99 | 0.0043648 | 0.636397468 | -0.652 |
| **894** | Elongation factor 1-beta OS=Canis lupus familiaris OX=9615 GN=EEF1B2 PE=3 SV=1 | A0A8I3PJ02 | *EEF1B2* | 25 kDa | 0.99 | 0.0043648 | 0.86154616 | -0.215 |
| **895** | Retinoic acid receptor responder 1 OS=Canis lupus familiaris OX=9615 GN=RARRES1 PE=3 SV=1 | A0A8I3PSZ2 | *RARRES1* | 32 kDa | 1 | 0 | 0.530343871 | -0.915 |
| **896** | Glucosamine-6-phosphate isomerase OS=Canis lupus familiaris OX=9615 GN=GNPDA1 PE=3 SV=1 | A0A8I3NTY8 | *GNPDA1* | 33 kDa | 1 | 0 | 0.636397468 | -0.652 |
| **897** | Pirin OS=Canis lupus familiaris OX=9615 GN=PIR PE=3 SV=1 | A0A8I3QK41 | *PIR* | 32 kDa | 1 | 0 | 1.004620069 | 0.00665 |
| **898** | COP9 signalosome subunit 2 OS=Canis lupus familiaris OX=9615 GN=COPS2 PE=4 SV=1 | A0A8I3PM77 | *COPS2* | 52 kDa | 1 | 0 | 1.0604672 | 0.0847 |
| **899** | Latent transforming growth factor beta binding protein 4 OS=Canis lupus familiaris OX=9615 GN=LTBP4 PE=4 SV=1 | A0A8I3MV94 | *LTBP4* | 173 kDa | 1 | 0 | 0.040666933 | -4.62 |
| **900** | Suprabasin OS=Canis lupus familiaris OX=9615 GN=SBSN PE=4 SV=1 | A0A8I3ML53 | *SBSN* | 77 kDa | 1 | 0 | 0.070805243 | -3.82 |
| **901** | Olfactomedin like 3 OS=Canis lupus familiaris OX=9615 GN=OLFML3 PE=4 SV=1 | A0A8I3PPB1 | *OLFML3* | 46 kDa | 1 | 0 | 0.081333866 | -3.62 |
| **902** | Ectonucleotide pyrophosphatase/phosphodiesterase 2 OS=Canis lupus familiaris OX=9615 GN=ENPP2 PE=4 SV=1 | A0A8I3N587 | *ENPP2* | 52 kDa | 1 | 0 | 0.081333866 | -3.62 |
| **903** | D-3-phosphoglycerate dehydrogenase OS=Canis lupus familiaris OX=9615 GN=LOC119863969 PE=3 SV=1 | A0A8I3N4A3 | *LOC119863969* | 57 kDa | 1 | 0 | 0.117440344 | -3.09 |
| **904** | Adenosine deaminase OS=Canis lupus familiaris OX=9615 GN=ADA PE=4 SV=1 | A0A8I3P1B4 | *ADA* | 38 kDa | 1 | 0 | 0.117440344 | -3.09 |
| **905** | Proliferating cell nuclear antigen OS=Canis lupus familiaris OX=9615 GN=PCNA PE=3 SV=1 | A0A8I3QKL0 | *PCNA* | 29 kDa | 1 | 0 | 0.132127255 | -2.92 |
| **906** | Crystallin zeta OS=Canis lupus familiaris OX=9615 GN=CRYZ PE=3 SV=1 | A0A8I3MSU3 | *CRYZ* | 32 kDa | 1 | 0 | 0.151774361 | -2.72 |

| | | | | | | | | | |
|---|---|---|---|---|---|---|---|---|---|
| **907** | Neuromodulin OS=Canis lupus familiaris OX=9615 GN=GAP43 PE=3 SV=1 | A0A8I3NKU6 | *GAP43* | 25 kDa | 1 | 0 | 0.151774361 | -2.72 |
| **908** | NSFL1 cofactor p47 OS=Canis lupus familiaris OX=9615 GN=NSFL1C PE=4 SV=1 | A0A8I3P8N0 | *NSFL1C* | 37 kDa | 1 | 0 | 0.151774361 | -2.72 |
| **909** | H1.3 linker histone, cluster member OS=Canis lupus familiaris OX=9615 GN=H1-3 PE=3 SV=1 | A0A8I3PXE6 | *H1-3* | 22 kDa | 1 | 0 | 0.151774361 | -2.72 |
| **910** | Collagen type XIV alpha 1 chain OS=Canis lupus familiaris OX=9615 GN=COL14A1 PE=4 SV=1 | A0A8I3MY18 | *COL14A1* | 175 kDa | 1 | 0 | 0.176776695 | -2.5 |
| **911** | Myosin heavy chain 7B OS=Canis lupus familiaris OX=9615 GN=MYH7B PE=3 SV=1 | A0A8I3PB38 | *MYH7B* | 214 kDa | 1 | 0 | 0.176776695 | -2.5 |
| **912** | Proteasome subunit alpha type OS=Canis lupus familiaris OX=9615 GN=PSMA2 PE=3 SV=1 | A0A8I3PCE5 | *PSMA2* | 26 kDa | 1 | 0 | 0.176776695 | -2.5 |
| **913** | Splicing factor 3b subunit 3 OS=Canis lupus familiaris OX=9615 GN=SF3B3 PE=4 SV=1 | A0A8I3MQG0 | *SF3B3* | 136 kDa | 1 | 0 | 0.176776695 | -2.5 |
| **914** | Uncharacterized protein OS=Canis lupus familiaris OX=9615 GN=LOC482977 PE=4 SV=1 | A0A8I3S6K3 | *LOC482977* | 32 kDa | 1 | 0 | 0.176776695 | -2.5 |
| **915** | Splicing factor 3a subunit 1 OS=Canis lupus familiaris OX=9615 GN=SF3A1 PE=4 SV=1 | A0A8I3PDS4 | *SF3A1* | 89 kDa | 1 | 0 | 0.176776695 | -2.5 |
| **916** | Platelet-derived growth factor receptor beta OS=Canis lupus familiaris OX=9615 GN=PDGFRB PE=3 SV=1 | A0A8I3N488 | *PDGFRB* | 126 kDa | 1 | 0 | 0.176776695 | -2.5 |
| **917** | Four and a half LIM domains 1 OS=Canis lupus familiaris OX=9615 GN=FHL1 PE=4 SV=1 | A0A8I3Q1V7 | *FHL1* | 22 kDa | 1 | 0 | 0.193445624 | -2.37 |
| **918** | Tripeptidyl-peptidase 1 OS=Canis lupus familiaris OX=9615 GN=TPP1 PE=4 SV=1 | A0A8I3NCG4 | *TPP1* | 65 kDa | 1 | 0 | 0.193445624 | -2.37 |
| **919** | Stathmin OS=Canis lupus familiaris OX=9615 GN=STMN1 PE=3 SV=1 | A0A8I3NR64 | *STMN1* | 21 kDa | 1 | 0 | 0.211686328 | -2.24 |
| **920** | Exportin-2 OS=Canis lupus familiaris OX=9615 GN=CSE1L PE=3 SV=1 | A0A8I3PFQ8 | *CSE1L* | 111 kDa | 1 | 0 | 0.211686328 | -2.24 |
| **921** | Inositol-1-monophosphatase OS=Canis lupus familiaris OX=9615 GN=IMPA1 PE=3 SV=1 | A0A8I3P0D5 | *IMPA1* | 22 kDa | 1 | 0 | 0.211686328 | -2.24 |
| **922** | Eukaryotic translation initiation factor 5 OS=Canis lupus familiaris OX=9615 GN=EIF5 PE=3 SV=1 | A0A8I3S343 | *EIF5* | 49 kDa | 1 | 0 | 0.211686328 | -2.24 |
| **923** | Small ribosomal subunit protein uS5 OS=Canis lupus familiaris OX=9615 GN=LOC608162 PE=3 SV=1 | A0A8I3N9X5 | *LOC608162* | 30 kDa | 1 | 0 | 0.211686328 | -2.24 |
| **924** | Collagen type XII alpha 1 chain OS=Canis lupus familiaris OX=9615 GN=COL12A1 PE=4 SV=1 | A0A8I3NL35 | *COL12A1* | 333 kDa | 1 | 0 | 0.211686328 | -2.24 |
| **925** | Periostin OS=Canis lupus familiaris OX=9615 GN=POSTN PE=4 SV=1 | A0A8I3QCL8 | *POSTN* | 90 kDa | 1 | 0 | 0.211686328 | -2.24 |
| **926** | Fibulin 5 OS=Canis lupus familiaris OX=9615 GN=FBLN5 PE=4 SV=1 | A0A8I3PA99 | *FBLN5* | 49 kDa | 1 | 0 | 0.211686328 | -2.24 |
| **927** | Mannose receptor C type 2 OS=Canis lupus familiaris OX=9615 GN=MRC2 PE=4 SV=1 | A0A8I3NWU4 | *MRC2* | 166 kDa | 1 | 0 | 0.244855074 | -2.03 |
| **928** | Collagen type VI alpha 3 chain OS=Canis lupus familiaris OX=9615 GN=COL6A3 PE=4 SV=1 | A0A8I3PY01 | *COL6A3* | 135 kDa | 1 | 0 | 0.25 | -2 |
| **929** | Large ribosomal subunit protein eL20 OS=Canis lupus familiaris OX=9615 GN=LOC119864697 PE=3 SV=1 | A0A8I3MZC4 | *LOC119864697* | 16 kDa | 1 | 0 | 0.26425451 | -1.92 |
| **930** | 3'(2'), 5'-bisphosphate nucleotidase 1 OS=Canis lupus familiaris OX=9615 GN=BPNT1 PE=3 SV=1 | A0A8I3Q582 | *BPNT1* | 35 kDa | 1 | 0 | 0.26425451 | -1.92 |

| | | | | | | | | |
|---|---|---|---|---|---|---|---|---|
| **931** | ADP ribosylation factor 4 OS=Canis lupus familiaris OX=9615 GN=ARF4 PE=3 SV=1 | A0A8I3P9K0 | *ARF4* | 18 kDa | 1 | 0 | 0.26425451 | -1.92 |
| **932** | Endoribonuclease LACTB2 OS=Canis lupus familiaris OX=9615 GN=LACTB2 PE=3 SV=1 | A0A8I3PYE9 | *LACTB2* | 33 kDa | 1 | 0 | 0.26425451 | -1.92 |
| **933** | Protein arginine methyltransferase 1 OS=Canis lupus familiaris OX=9615 GN=PRMT1 PE=4 SV=1 | A0A8I3PGA1 | *PRMT1* | 33 kDa | 1 | 0 | 0.26425451 | -1.92 |
| **934** | G protein pathway suppressor 1 OS=Canis lupus familiaris OX=9615 GN=GPS1 PE=4 SV=1 | A0A8I3NIR2 | *GPS1* | 74 kDa | 1 | 0 | 0.26425451 | -1.92 |
| **935** | ADP ribosylation factor like GTPase 3 OS=Canis lupus familiaris OX=9615 GN=ARL3 PE=3 SV=1 | A0A8I3PU32 | *ARL3* | 19 kDa | 1 | 0 | 0.26425451 | -1.92 |
| **936** | Microfibril associated protein 2 OS=Canis lupus familiaris OX=9615 GN=MFAP2 PE=3 SV=1 | A0A8I3MLA0 | *MFAP2* | 21 kDa | 1 | 0 | 0.26425451 | -1.92 |
| **937** | Creatine kinase M-type OS=Canis lupus familiaris OX=9615 GN=CKM PE=1 SV=3 | P05123 | *CKM* | 43 kDa | 1 | 0 | 0.26425451 | -1.92 |
| **938** | Amino acid transporter OS=Canis lupus familiaris OX=9615 GN=SLC1A5 PE=3 SV=1 | A0A8I3MQQ4 | *SLC1A5* | 57 kDa | 1 | 0 | 0.353553391 | -1.5 |
| **939** | Cation-independent mannose-6-phosphate/insulin-like growth factor 2 receptor protein OS=Canis lupus familiaris OX=9615 GN=CI-MPR/IGF2R PE=2 SV=1 | B1H0W0 | *CI-MPR/IGF2R* | 275 kDa | 1 | 0 | 0.353553391 | -1.5 |
| **940** | Dynactin subunit 2 OS=Canis lupus familiaris OX=9615 GN=DCTN2 PE=3 SV=1 | A0A8I3NX29 | *DCTN2* | 45 kDa | 1 | 0 | 0.353553391 | -1.5 |
| **941** | Vacuolar protein sorting-associated protein 29 OS=Canis lupus familiaris OX=9615 GN=VPS29 PE=3 SV=1 | A0A8I3S6P0 | *VPS29* | 21 kDa | 1 | 0 | 0.353553391 | -1.5 |
| **942** | Splicing factor 3b subunit 2 OS=Canis lupus familiaris OX=9615 GN=SF3B2 PE=4 SV=1 | A0A8I3NV69 | *SF3B2* | 100 kDa | 1 | 0 | 0.353553391 | -1.5 |
| **943** | 26S proteasome non-ATPase regulatory subunit 1 OS=Canis lupus familiaris OX=9615 GN=PSMD1 PE=3 SV=1 | A0A8I3PPD3 | *PSMD1* | 102 kDa | 1 | 0 | 0.353553391 | -1.5 |
| **944** | Myosin light chain 11 OS=Canis lupus familiaris OX=9615 GN=MYL11 PE=4 SV=1 | A0A8I3P221 | *MYL11* | 17 kDa | 1 | 0 | 0.353553391 | -1.5 |
| **945** | Trifunctional purine biosynthetic protein adenosine-3 OS=Canis lupus familiaris OX=9615 GN=GART PE=3 SV=1 | A0A8I3PRU8 | *GART* | 108 kDa | 1 | 0 | 0.353553391 | -1.5 |
| **946** | Phospholipase B-like OS=Canis lupus familiaris OX=9615 GN=PLBD2 PE=3 SV=1 | A0A8I3S7X3 | *PLBD2* | 62 kDa | 1 | 0 | 0.353553391 | -1.5 |
| **947** | Angiopoietin like 1 OS=Canis lupus familiaris OX=9615 GN=ANGPTL1 PE=4 SV=1 | A0A8I3N7B9 | *ANGPTL1* | 57 kDa | 1 | 0 | 0.353553391 | -1.5 |
| **948** | Latent transforming growth factor beta binding protein 4 OS=Canis lupus familiaris OX=9615 GN=LTBP4 PE=4 SV=1 | A0A8I3RSW0 | *LTBP4* | 175 kDa | 1 | 0 | 0.353553391 | -1.5 |
| **949** | Inter-alpha-trypsin inhibitor heavy chain 5 OS=Canis lupus familiaris OX=9615 GN=ITIH5 PE=4 SV=1 | A0A8I3MIG0 | *ITIH5* | 93 kDa | 1 | 0 | 0.386891248 | -1.37 |
| **950** | Uncharacterized protein OS=Canis lupus familiaris OX=9615 GN=ALDH9A1 PE=3 SV=1 | A0A8I3S4S9 | *ALDH9A1* | 56 kDa | 1 | 0 | 0.423372656 | -1.24 |
| **951** | Galactose mutarotase OS=Canis lupus familiaris OX=9615 GN=GALM PE=4 SV=1 | A0A8I3S2B9 | *GALM* | 43 kDa | 1 | 0 | 0.423372656 | -1.24 |
| **952** | WAP, follistatin/kazal, immunoglobulin, kunitz and netrin domain containing 1 OS=Canis lupus familiaris OX=9615 GN=WFIKKN1 PE=3 SV=1 | A0A8I3QAQ1 | *WFIKKN1* | 66 kDa | 1 | 0 | 0.423372656 | -1.24 |
| **953** | Peptidase domain containing associated with muscle regeneration 1 OS=Canis lupus familiaris OX=9615 GN=PAMR1 PE=4 SV=1 | A0A8I3PCJ1 | *PAMR1* | 67 kDa | 1 | 0 | 0.453759578 | -1.14 |

| | | | | | | | | |
|---|---|---|---|---|---|---|---|---|
| **954** | phosphoserine phosphatase OS=Canis lupus familiaris OX=9615 GN=PSPH PE=3 SV=1 | A0A8I3NC12 | *PSPH* | 25 kDa | 1 | 0 | 0.453759578 | -1.14 |
| **955** | Obg like ATPase 1 OS=Canis lupus familiaris OX=9615 GN=OLA1 PE=4 SV=1 | A0A8I3Q3S6 | *OLA1* | 31 kDa | 1 | 0 | 0.453759578 | -1.14 |
| **956** | Uncharacterized protein OS=Canis lupus familiaris OX=9615 GN=DPP7 PE=3 SV=1 | A0A8I3PJI1 | *DPP7* | 60 kDa | 1 | 0 | 0.453759578 | -1.14 |
| **957** | Sodium/potassium-transporting ATPase subunit alpha OS=Canis lupus familiaris OX=9615 GN=ATP1A1 PE=3 SV=1 | A0A8I3QHE9 | *ATP1A1* | 113 kDa | 1 | 0 | 0.530343871 | -0.915 |
| **958** | Matrix metalloproteinase-14 OS=Canis lupus familiaris OX=9615 GN=MMP14 PE=3 SV=1 | A0A8I3MND9 | *MMP14* | 66 kDa | 1 | 0 | 0.530343871 | -0.915 |
| **959** | Tankyrase 1 binding protein 1 OS=Canis lupus familiaris OX=9615 GN=TNKS1BP1 PE=4 SV=1 | A0A8I3S5F5 | *TNKS1BP1* | 181 kDa | 1 | 0 | 0.530343871 | -0.915 |
| **960** | Myosin light chain 1 OS=Canis lupus familiaris OX=9615 GN=MYL1 PE=4 SV=1 | A0A8I3P4G4 | *MYL1* | 26 kDa | 1 | 0 | 0.530343871 | -0.915 |
| **961** | Eukaryotic translation initiation factor 3 subunit M OS=Canis lupus familiaris OX=9615 GN=EIF3M PE=3 SV=1 | A0A8I3PRI3 | *EIF3M* | 28 kDa | 1 | 0 | 0.530343871 | -0.915 |
| **962** | SEC24 homolog D, COPII coat complex component OS=Canis lupus familiaris OX=9615 GN=SEC24D PE=4 SV=1 | A0A8I3QW97 | *SEC24D* | 44 kDa | 1 | 0 | 0.530343871 | -0.915 |
| **963** | Sorting nexin 5 OS=Canis lupus familiaris OX=9615 GN=SNX5 PE=3 SV=1 | A0A8I3S2I8 | *SNX5* | 51 kDa | 1 | 0 | 0.530343871 | -0.915 |
| **964** | ATP binding cassette subfamily E member 1 OS=Canis lupus familiaris OX=9615 GN=ABCE1 PE=4 SV=1 | A0A8I3NJV9 | *ABCE1* | 65 kDa | 1 | 0 | 0.530343871 | -0.915 |
| **965** | Valine--tRNA ligase OS=Canis lupus familiaris OX=9615 GN=VARS1 PE=3 SV=1 | A0A8I3NMY0 | *VARS1* | 140 kDa | 1 | 0 | 0.530343871 | -0.915 |
| **966** | Glutamyl-prolyl-tRNA synthetase 1 OS=Canis lupus familiaris OX=9615 GN=EPRS1 PE=3 SV=1 | A0A8I3SBI9 | *EPRS1* | 168 kDa | 1 | 0 | 0.530343871 | -0.915 |
| **967** | Serum amyloid A protein OS=Canis lupus familiaris OX=9615 GN=LOC403585 PE=3 SV=1 | A0A8I3NX87 | *LOC403585* | 14 kDa | 1 | 0 | 0.530343871 | -0.915 |
| **968** | Immunity related GTPase Q OS=Canis lupus familiaris OX=9615 GN=IRGQ PE=3 SV=1 | A0A8I3M9Y7 | *IRGQ* | 65 kDa | 1 | 0 | 0.530343871 | -0.915 |
| **969** | Neural cell adhesion molecule 1 OS=Canis lupus familiaris OX=9615 GN=NCAM1 PE=4 SV=1 | A0A8I3MN47 | *NCAM1* | 119 kDa | 1 | 0 | 0.530343871 | -0.915 |
| **970** | N-acyl-aliphatic-L-amino acid amidohydrolase OS=Canis lupus familiaris OX=9615 GN=ACY1 PE=3 SV=1 | A0A8I3QUB8 | *ACY1* | 77 kDa | 1 | 0 | 0.530343871 | -0.915 |
| **971** | Heat shock 70 kDa protein 13 OS=Canis lupus familiaris OX=9615 GN=HSPA13 PE=2 SV=1 | J9NU25 | *HSPA13* | 52 kDa | 1 | 0 | 0.530343871 | -0.915 |
| **972** | Small ubiquitin like modifier 1 OS=Canis lupus familiaris OX=9615 GN=SUMO1 PE=3 SV=1 | A0A8I3NZS0 | *SUMO1* | 29 kDa | 1 | 0 | 0.530343871 | -0.915 |
| **973** | dimethylargininase OS=Canis lupus familiaris OX=9615 GN=DDAH1 PE=3 SV=1 | A0A8I3MHL9 | *DDAH1* | 21 kDa | 1 | 0 | 0.530343871 | -0.915 |
| **974** | Pro-adrenomedullin OS=Canis lupus familiaris OX=9615 GN=ADM PE=3 SV=1 | A0A8I3RY48 | *ADM* | 21 kDa | 1 | 0 | 0.530343871 | -0.915 |
| **975** | CXXC motif containing zinc binding protein OS=Canis lupus familiaris OX=9615 GN=CZIB PE=3 SV=1 | A0A8I3P3G1 | *CZIB* | 24 kDa | 1 | 0 | 0.530343871 | -0.915 |
| **976** | ADAM metallopeptidase domain 12 OS=Canis lupus familiaris OX=9615 GN=ADAM12 PE=4 SV=1 | A0A8I3PTF2 | *ADAM12* | 99 kDa | 1 | 0 | 0.530343871 | -0.915 |
| **977** | Immunoglobulin superfamily containing leucine rich repeat OS=Canis lupus familiaris OX=9615 GN=ISLR PE=4 SV=1 | A0A8I3Q1S9 | *ISLR* | 46 kDa | 1 | 0 | 0.530343871 | -0.915 |

| | | | | | | | | |
|---|---|---|---|---|---|---|---|---|
| **978** | Peptidase domain containing associated with muscle regeneration 1 OS=Canis lupus familiaris OX=9615 GN=PAMR1 PE=4 SV=1 | A0A8I3S4T0 | *PAMR1* | 81 kDa | 1 | 0 | 0.530343871 | -0.915 |
| **979** | Alpha-L-fucosidase OS=Canis lupus familiaris OX=9615 GN=FUCA1 PE=3 SV=1 | A0A8I3P3M7 | *FUCA1* | 51 kDa | 1 | 0 | 0.530343871 | -0.915 |
| **980** | Transforming growth factor beta receptor 3 OS=Canis lupus familiaris OX=9615 GN=TGFBR3 PE=4 SV=1 | A0A8I3RZ63 | *TGFBR3* | 94 kDa | 1 | 0 | 0.596667872 | -0.745 |
| **981** | CRK proto-oncogene, adaptor protein OS=Canis lupus familiaris OX=9615 GN=CRK PE=3 SV=1 | A0A8I3NGQ0 | *CRK* | 46 kDa | 1 | 0 | 0.636397468 | -0.652 |
| **982** | Prefoldin subunit 6 OS=Canis lupus familiaris OX=9615 GN=PFDN6 PE=3 SV=1 | A0A8I3PCA3 | *PFDN6* | 11 kDa | 1 | 0 | 0.707106781 | -0.5 |
| **983** | Malate dehydrogenase OS=Canis lupus familiaris OX=9615 PE=3 SV=1 | A0A8I3S3J3 | | 36 kDa | 1 | 0 | 0.707106781 | -0.5 |
| **984** | Prothymosin alpha OS=Canis lupus familiaris OX=9615 GN=PTMA PE=3 SV=1 | A0A8I3PVA7 | *PTMA* | 14 kDa | 1 | 0 | 0.707106781 | -0.5 |
| **985** | Nuclear mitotic apparatus protein 1 OS=Canis lupus familiaris OX=9615 GN=NUMA1 PE=4 SV=1 | A0A8I3N6X9 | *NUMA1* | 238 kDa | 1 | 0 | 0.707106781 | -0.5 |
| **986** | CutA divalent cation tolerance homolog OS=Canis lupus familiaris OX=9615 GN=CUTA PE=3 SV=1 | A0A8I3PMV7 | *CUTA* | 17 kDa | 1 | 0 | 0.707106781 | -0.5 |
| **987** | alpha-1,2-Mannosidase OS=Canis lupus familiaris OX=9615 GN=MAN1A1 PE=3 SV=1 | A0A8I3N7J6 | *MAN1A1* | 73 kDa | 1 | 0 | 0.707106781 | -0.5 |
| **988** | Uncharacterized protein OS=Canis lupus familiaris OX=9615 PE=4 SV=1 | A0A8I3RR07 | | 29 kDa | 1 | 0 | 0.795536484 | -0.33 |
| **989** | Carboxypeptidase X, M14 family member 1 OS=Canis lupus familiaris OX=9615 GN=CPXM1 PE=3 SV=1 | A0A8I3NVC8 | *CPXM1* | 82 kDa | 1 | 0 | 0.795536484 | -0.33 |
| **990** | Ubiquitin conjugating enzyme E2 N OS=Canis lupus familiaris OX=9615 GN=UBE2N PE=3 SV=1 | A0A8I3P6I7 | *UBE2N* | 17 kDa | 1 | 0 | 1.0604672 | 0.0847 |
| **991** | Large ribosomal subunit protein uL2 OS=Canis lupus familiaris OX=9615 GN=RPL8 PE=3 SV=1 | A0A8I3RWX4 | *RPL8* | 28 kDa | 1 | 0 | 1.0604672 | 0.0847 |
| **992** | Large ribosomal subunit protein uL13 OS=Canis lupus familiaris OX=9615 GN=RPL13A PE=3 SV=1 | A0A8I3PH30 | *RPL13A* | 24 kDa | 1 | 0 | 1.0604672 | 0.0847 |
| **993** | RuvB-like helicase OS=Canis lupus familiaris OX=9615 GN=RUVBL2 PE=3 SV=1 | A0A8I3MBF5 | *RUVBL2* | 51 kDa | 1 | 0 | 1.0604672 | 0.0847 |
| **994** | High mobility group box 2 OS=Canis lupus familiaris OX=9615 GN=HMGB2 PE=3 SV=1 | A0A8I3P9Q5 | *HMGB2* | 24 kDa | 1 | 0 | 1.0604672 | 0.0847 |
| **995** | Uncharacterized protein OS=Canis lupus familiaris OX=9615 GN=PAFAH1B3 PE=4 SV=1 | A0A8I3MH35 | *PAFAH1B3* | 26 kDa | 1 | 0 | 1.0604672 | 0.0847 |
| **996** | T-complex protein 1 subunit gamma OS=Canis lupus familiaris OX=9615 GN=CCT3 PE=3 SV=1 | A0A8I3RV88 | *CCT3* | 61 kDa | 1 | 0 | 1.0604672 | 0.0847 |
| **997** | Protein kinase C and casein kinase substrate in neurons 2 OS=Canis lupus familiaris OX=9615 GN=PACSIN2 PE=3 SV=1 | A0A8I3NUA9 | *PACSIN2* | 51 kDa | 1 | 0 | 1.0604672 | 0.0847 |
| **998** | Eukaryotic translation initiation factor 5B OS=Canis lupus familiaris OX=9615 GN=EIF5B PE=3 SV=1 | A0A8I3PAX1 | *EIF5B* | 139 kDa | 1 | 0 | 1.0604672 | 0.0847 |
| **999** | Coagulation factor V OS=Canis lupus familiaris OX=9615 GN=F5 PE=3 SV=1 | A0A8I3MSW8 | *F5* | 248 kDa | 1 | 0 | 1.0604672 | 0.0847 |
| **1000** | Prefoldin subunit 3 OS=Canis lupus familiaris OX=9615 GN=VBP1 PE=3 SV=1 | A0A8I3P4S3 | *VBP1* | 22 kDa | 1 | 0 | 1.0604672 | 0.0847 |

| | | | | | | | | |
|---|---|---|---|---|---|---|---|---|
| **1001** | Uncharacterized protein OS=Canis lupus familiaris OX=9615 PE=4 SV=1 | A0A8I3PJM3 | | 84 kDa | 1 | 0 | 1.0604672 | 0.0847 |
| **1002** | Biotinidase OS=Canis lupus familiaris OX=9615 GN=BTD PE=3 SV=1 | A0A8I3S835 | *BTD* | 60 kDa | 1 | 0 | 1.0604672 | 0.0847 |
| **1003** | SEC24 homolog D, COPII coat complex component OS=Canis lupus familiaris OX=9615 GN=SEC24D PE=3 SV=1 | A0A8I3SCF9 | *SEC24D* | 113 kDa | 1 | 0 | 1.0604672 | 0.0847 |
| **1004** | Xaa-Arg dipeptidase OS=Canis lupus familiaris OX=9615 GN=PM20D2 PE=3 SV=1 | A0A8I3N3F7 | *PM20D2* | 47 kDa | 1 | 0 | 1.0604672 | 0.0847 |
| **1005** | 40S ribosomal protein S15a OS=Canis lupus familiaris OX=9615 GN=RPS15A PE=3 SV=1 | A0A8I3MV29 | *RPS15A* | 11 kDa | 1 | 0 | 1.0604672 | 0.0847 |
| **1006** | COP9 signalosome subunit 7A OS=Canis lupus familiaris OX=9615 GN=COPS7A PE=3 SV=1 | A0A8I3QDI2 | *COPS7A* | 29 kDa | 1 | 0 | 1.0604672 | 0.0847 |
| **1007** | Vacuolar proton pump subunit B OS=Canis lupus familiaris OX=9615 GN=ATP6V1B2 PE=3 SV=1 | A0A8I3Q2N9 | *ATP6V1B2* | 57 kDa | 1 | 0 | 1.0604672 | 0.0847 |
| **1008** | Signal recognition particle subunit SRP68 OS=Canis lupus familiaris OX=9615 GN=SRP68 PE=3 SV=1 | A0A8I3PK44 | *srp.68* | 70 kDa | 1 | 0 | 1.0604672 | 0.0847 |
| **1009** | Adenine phosphoribosyltransferase OS=Canis lupus familiaris OX=9615 GN=APRT PE=3 SV=1 | A0A8I3MH54 | *APRT* | 20 kDa | 1 | 0 | 1.0604672 | 0.0847 |
| **1010** | Beta-1,4-glucuronyltransferase 1 OS=Canis lupus familiaris OX=9615 GN=B4GAT1 PE=3 SV=1 | A0A8I3NME9 | *B4GAT1* | 70 kDa | 1 | 0 | 1.0604672 | 0.0847 |
| **1011** | Follistatin like 3 OS=Canis lupus familiaris OX=9615 GN=FSTL3 PE=4 SV=1 | A0A8I3NY94 | *FSTL3* | 28 kDa | 1 | 0 | 1.0604672 | 0.0847 |
| **1012** | AP-1 complex subunit gamma OS=Canis lupus familiaris OX=9615 GN=AP1G1 PE=3 SV=1 | A0A8I3N275 | *AP1G1* | 92 kDa | 1 | 0 | 1.0604672 | 0.0847 |
| **1013** | Acidic leucine-rich nuclear phosphoprotein 32 family member OS=Canis lupus familiaris OX=9615 GN=ANP32A PE=3 SV=1 | A0A8I3S455 | *ANP32A* | 29 kDa | 1 | 0 | 1.0604672 | 0.0847 |
| **1014** | Extracellular matrix protein 2 OS=Canis lupus familiaris OX=9615 GN=ECM2 PE=4 SV=1 | A0A8I3PNB4 | *ECM2* | 73 kDa | 1 | 0 | 1.0604672 | 0.0847 |
| **1015** | Scavenger receptor cysteine rich family member with 5 domains OS=Canis lupus familiaris OX=9615 GN=SSC5D PE=4 SV=1 | A0A8I3MBL4 | *SSC5D* | 161 kDa | 1 | 0 | 1.0604672 | 0.0847 |
| **1016** | Gremlin 1, DAN family BMP antagonist OS=Canis lupus familiaris OX=9615 PE=3 SV=1 | A0A8I3SCY1 | | 26 kDa | 1 | 0 | 1.32592576 | 0.407 |
| **1017** | Heterogeneous nuclear ribonucleoprotein R OS=Canis lupus familiaris OX=9615 GN=HNRNPR PE=4 SV=1 | A0A8I3NWS4 | *HNRNPR* | 71 kDa | 1 | 0 | 1.591072968 | 0.67 |
| **1018** | Interleukin 18 receptor 1 OS=Canis lupus familiaris OX=9615 GN=IL1RL1 PE=3 SV=1 | A0A8I3RXZ8 | *IL1RL1* | 60 kDa | 1 | 0 | 1.591072968 | 0.67 |
| **1019** | HGF activator OS=Canis lupus familiaris OX=9615 GN=HGFAC PE=4 SV=1 | A0A8I3NC74 | *HGFAC* | 70 kDa | 1 | 0 | 2.114036081 | 1.08 |
| **1020** | Ribosomal protein L7 OS=Canis lupus familiaris OX=9615 GN=RPL7 PE=3 SV=1 | A0A8I3Q3I5 | *RPL7* | 30 kDa | 1 | 0 | 2.114036081 | 1.08 |
| **1021** | Ribosomal protein L26 OS=Canis lupus familiaris OX=9615 GN=RPL26 PE=3 SV=1 | A0A8I3MIA0 | *RPL26* | 13 kDa | 1 | 0 | 2.114036081 | 1.08 |
| **1022** | Stress-70 protein, mitochondrial OS=Canis lupus familiaris OX=9615 GN=HSPA9 PE=3 SV=1 | A0A8I3PQ37 | *HSPA9* | 74 kDa | 1 | 0 | 2.114036081 | 1.08 |
| **1023** | Solute carrier family 3 member 2 OS=Canis lupus familiaris OX=9615 GN=SLC3A2 PE=4 SV=1 | A0A8I3MWJ8 | *SLC3A2* | 58 kDa | 1 | 0 | 2.114036081 | 1.08 |

| | | | | | | | | |
|---|---|---|---|---|---|---|---|---|
| **1024** | Adipogenesis regulatory factor OS=Canis lupus familiaris OX=9615 GN=ADIRF PE=4 SV=1 | A0A8I3RTQ4 | *ADIRF* | 8 kDa | 1 | 0 | 2.114036081 | 1.08 |
| **1025** | Calpain 1 OS=Canis lupus familiaris OX=9615 GN=CAPN1 PE=3 SV=1 | A0A8I3PQP3 | *CAPN1* | 78 kDa | 1 | 0 | 2.114036081 | 1.08 |
| **1026** | RuvB-like helicase OS=Canis lupus familiaris OX=9615 GN=RUVBL1 PE=3 SV=1 | A0A8I3P6G1 | *RUVBL1* | 50 kDa | 1 | 0 | 2.114036081 | 1.08 |
| **1027** | Uncharacterized protein OS=Canis lupus familiaris OX=9615 GN=LOC607874 PE=4 SV=1 | A0A8I3QNP3 | *LOC607874* | 16 kDa | 1 | 0 | 2.114036081 | 1.08 |
| **1028** | Calponin OS=Canis lupus familiaris OX=9615 GN=CNN3 PE=3 SV=1 | A0A8I3NQX1 | *CNN3* | 36 kDa | 1 | 0 | 2.114036081 | 1.08 |
| **1029** | Matrin 3 OS=Canis lupus familiaris OX=9615 GN=MATR3 PE=4 SV=1 | A0A8I3PJD9 | *MATR3* | 93 kDa | 1 | 0 | 2.114036081 | 1.08 |
| **1030** | NSF attachment protein alpha OS=Canis lupus familiaris OX=9615 GN=NAPA PE=3 SV=1 | A0A8I3MLQ0 | *NAPA* | 33 kDa | 1 | 0 | 2.114036081 | 1.08 |
| **1031** | Glucose-6-phosphate 1-dehydrogenase OS=Canis lupus familiaris OX=9615 GN=G6PD PE=3 SV=1 | A0A8I3Q7C5 | *G6PD* | 70 kDa | 1 | 0 | 2.114036081 | 1.08 |
| **1032** | Procollagen C-endopeptidase enhancer 2 OS=Canis lupus familiaris OX=9615 GN=PCOLCE2 PE=4 SV=1 | A0A8I3Q5D9 | *PCOLCE2* | 44 kDa | 1 | 0 | 2.114036081 | 1.08 |
| **1033** | RNA transcription, translation and transport factor protein OS=Canis lupus familiaris OX=9615 GN=RTRAF PE=3 SV=1 | A0A8I3MKM6 | *RTRAF* | 28 kDa | 1 | 0 | 2.114036081 | 1.08 |
| **1034** | Tubulin tyrosine ligase like 12 OS=Canis lupus familiaris OX=9615 GN=TTLL12 PE=4 SV=1 | A0A8I3NI92 | *TTLL12* | 92 kDa | 1 | 0 | 2.114036081 | 1.08 |
| **1035** | W2 domain-containing protein OS=Canis lupus familiaris OX=9615 PE=3 SV=1 | A0A8I3Q7M7 | | 30 kDa | 1 | 0 | 2.114036081 | 1.08 |
| **1036** | LDL receptor related protein 8 OS=Canis lupus familiaris OX=9615 GN=LRP8 PE=4 SV=1 | A0A8I3P1F0 | *LRP8* | 97 kDa | 1 | 0 | 2.114036081 | 1.08 |
| **1037** | High density lipoprotein binding protein OS=Canis lupus familiaris OX=9615 GN=HDLBP PE=4 SV=1 | A0A8I3PSM2 | *HDLBP* | 142 kDa | 1 | 0 | 2.114036081 | 1.08 |
| **1038** | Four and a half LIM domains 2 OS=Canis lupus familiaris OX=9615 GN=FHL2 PE=4 SV=1 | A0A8I3NEJ6 | *FHL2* | 32 kDa | 1 | 0 | 2.114036081 | 1.08 |
| **1039** | Serine/threonine-protein phosphatase 2A activator OS=Canis lupus familiaris OX=9615 GN=PTPA PE=3 SV=1 | A0A8I3PL17 | *PTPA* | 32 kDa | 1 | 0 | 2.114036081 | 1.08 |
| **1040** | ADAM metallopeptidase domain 10 OS=Canis lupus familiaris OX=9615 GN=ADAM10 PE=4 SV=1 | A0A8I3P0M4 | *ADAM10* | 84 kDa | 1 | 0 | 2.114036081 | 1.08 |
| **1041** | Nidogen 1 OS=Canis lupus familiaris OX=9615 GN=NID1 PE=4 SV=1 | A0A8I3MUT8 | *NID1* | 136 kDa | 1 | 0 | 2.114036081 | 1.08 |
| **1042** | Integrin subunit alpha 5 OS=Canis lupus familiaris OX=9615 GN=ITGA5 PE=3 SV=1 | A0A8I3S2B2 | *ITGA5* | 114 kDa | 1 | 0 | 2.657371628 | 1.41 |
| **1043** | Prothrombin OS=Canis lupus familiaris OX=9615 GN=F2 PE=3 SV=1 | A0A8I3PUT1 | *F2* | 70 kDa | 1 | 0 | 3.182145935 | 1.67 |
| **1044** | Serine/threonine-protein phosphatase OS=Canis lupus familiaris OX=9615 GN=PPP1CB PE=3 SV=1 | A0A8I3NGF5 | *PPP1CB* | 37 kDa | 1 | 0 | 3.182145935 | 1.67 |
| **1045** | Heat shock protein 105 kDa OS=Canis lupus familiaris OX=9615 GN=HSPH1 PE=3 SV=1 | A0A8I3PBN0 | *HSPH1* | 92 kDa | 1 | 0 | 3.182145935 | 1.67 |
| **1046** | PML-RARA regulated adaptor molecule 1 OS=Canis lupus familiaris OX=9615 GN=PRAM1 PE=4 SV=1 | A0A8I3RUW9 | *PRAM1* | 66 kDa | 1 | 0 | 3.182145935 | 1.67 |
| **1047** | Glutathione S-transferase omega OS=Canis lupus familiaris OX=9615 GN=GSTO1 PE=3 SV=1 | A0A8I3Q1F5 | *GSTO1* | 24 kDa | 1 | 0 | 3.182145935 | 1.67 |

| | | | | | | | | |
|---|---|---|---|---|---|---|---|---|
| **1048** | Complement C7 OS=Canis lupus familiaris OX=9615 GN=C7 PE=3 SV=1 | A0A8I3MQ72 | *C7* | 95 kDa | 1 | 0 | 3.182145935 | 1.67 |
| **1049** | Proteasome 26S subunit, non-ATPase 3 OS=Canis lupus familiaris OX=9615 GN=PSMD3 PE=3 SV=1 | A0A8I3NH42 | *PSMD3* | 61 kDa | 1 | 0 | 3.182145935 | 1.67 |
| **1050** | Reticulocalbin 3 OS=Canis lupus familiaris OX=9615 GN=RCN3 PE=4 SV=1 | A0A8I3PNJ3 | *RCN3* | 38 kDa | 1 | 0 | 3.182145935 | 1.67 |
| **1051** | Malignant T-cell-amplified sequence OS=Canis lupus familiaris OX=9615 GN=MCTS1 PE=3 SV=1 | A0A8I3PNX2 | *MCTS1* | 21 kDa | 1 | 0 | 3.182145935 | 1.67 |
| **1052** | Proline and arginine rich end leucine rich repeat protein OS=Canis lupus familiaris OX=9615 GN=PRELP PE=4 SV=1 | A0A8I3S110 | *PRELP* | 43 kDa | 1 | 0 | 3.182145935 | 1.67 |
| **1053** | Uncharacterized protein OS=Canis lupus familiaris OX=9615 GN=COPS8 PE=4 SV=1 | A0A8I3Q3Z1 | *COPS8* | 19 kDa | 1 | 0 | 3.182145935 | 1.67 |
| **1054** | Bridging integrator 1 OS=Canis lupus familiaris OX=9615 GN=BIN1 PE=4 SV=1 | A0A8I3PFM7 | *BIN1* | 76 kDa | 1 | 0 | 3.182145935 | 1.67 |
| **1055** | Sorcin OS=Canis lupus familiaris OX=9615 GN=SRI PE=4 SV=1 | A0A8I3RY81 | *SRI* | 22 kDa | 1 | 0 | 3.182145935 | 1.67 |
| **1056** | Poly(A) binding protein interacting protein 1 OS=Canis lupus familiaris OX=9615 GN=PAIP1 PE=4 SV=1 | A0A8I3MEB1 | *PAIP1* | 40 kDa | 1 | 0 | 3.182145935 | 1.67 |
| **1057** | Prostaglandin E synthase 3 OS=Canis lupus familiaris OX=9615 GN=ATP5F1B PE=3 SV=1 | A0A8I3NAU5 | *ATP5F1B* | 37 kDa | 1 | 0 | 4.228072162 | 2.08 |
| **1058** | Cadherin 13 OS=Canis lupus familiaris OX=9615 GN=CDH13 PE=4 SV=1 | A0A8I3P1Q7 | *CDH13* | 78 kDa | 1 | 0 | 4.228072162 | 2.08 |
| **1059** | Endothelial cell specific molecule 1 OS=Canis lupus familiaris OX=9615 GN=ESM1 PE=4 SV=1 | A0A8I3NHV3 | *ESM1* | 20 kDa | 1 | 0 | 4.228072162 | 2.08 |
| **1060** | Semaphorin 7A (John Milton Hagen blood group) OS=Canis lupus familiaris OX=9615 GN=SEMA7A PE=3 SV=1 | A0A8I3Q300 | *SEMA7A* | 73 kDa | 1 | 0 | 4.228072162 | 2.08 |
| **1061** | Caveolae associated protein 3 OS=Canis lupus familiaris OX=9615 GN=CAVIN3 PE=3 SV=1 | A0A8I3N1D8 | *CAVIN3* | 40 kDa | 1 | 0 | 4.228072162 | 2.08 |
| **1062** | Aminopeptidase like 1 OS=Canis lupus familiaris OX=9615 GN=NPEPL1 PE=3 SV=1 | A0A8I3NGL3 | *NPEPL1* | 56 kDa | 1 | 0 | 4.228072162 | 2.08 |
| **1063** | Four and a half LIM domains 3 OS=Canis lupus familiaris OX=9615 GN=FHL3 PE=4 SV=1 | A0A8I3QAY6 | *FHL3* | 32 kDa | 1 | 0 | 4.228072162 | 2.08 |
| **1064** | Uncharacterized protein OS=Canis lupus familiaris OX=9615 PE=4 SV=1 | A0A8I3PVD2 | | 36 kDa | 1 | 0 | 4.228072162 | 2.08 |
| **1065** | Cytochrome c oxidase subunit OS=Canis lupus familiaris OX=9615 GN=LOC612644 PE=3 SV=1 | A0A8I3MRB3 | *LOC612644* | 10 kDa | 1 | 0 | 4.228072162 | 2.08 |
| **1066** | Inter-alpha-trypsin inhibitor heavy chain 1 OS=Canis lupus familiaris OX=9615 GN=ITIH1 PE=3 SV=1 | A0A8I3Q1W7 | *ITIH1* | 100 kDa | 1 | 0 | 5.314743256 | 2.41 |
| **1067** | T-complex protein 1 subunit theta OS=Canis lupus familiaris OX=9615 GN=CCT8 PE=3 SV=1 | A0A8I3S0W8 | *CCT8* | 77 kDa | 1 | 0 | 6.36429187 | 2.67 |
| **1068** | Collagen type XVIII alpha 1 chain OS=Canis lupus familiaris OX=9615 GN=COL18A1 PE=4 SV=1 | A0A8I3S342 | *COL18A1* | 133 kDa | 1 | 0 | 6.91629785 | 2.79 |
| **1069** | Matrix remodeling associated 5 OS=Canis lupus familiaris OX=9615 GN=MXRA5 PE=4 SV=1 | A0A8I3PLF3 | *MXRA5* | 305 kDa | -- | | #VALUE! | Missing Value |
| **1070** | Myosin heavy chain 4 OS=Canis lupus familiaris OX=9615 GN=MYH4 PE=3 SV=1 | A0A8I3RQQ3 | *MYH4* | 223 kDa | -- | | #VALUE! | Missing Value |
| **1071** | Prostaglandin F2 receptor inhibitor OS=Canis lupus familiaris OX=9615 GN=PTGFRN PE=4 SV=1 | A0A8I3Q6X7 | *PTGFRN* | 100 kDa | -- | | #VALUE! | Missing Value |

| | | | | | | | | |
|---|---|---|---|---|---|---|---|---|
| **1072** | Phosphoglucomutase 5 OS=Canis lupus familiaris OX=9615 GN=PGM5 PE=3 SV=1 | A0A8I3MPS0 | *PGM5* | 62 kDa | -- | | #VALUE! | Missing Value |
| **1073** | Rac family small GTPase 1 OS=Canis lupus familiaris OX=9615 GN=RAC1 PE=4 SV=1 | A0A8I3N6F6 | *RAC1* | 22 kDa | -- | | #VALUE! | Missing Value |
| **1074** | Sequestosome 1 OS=Canis lupus familiaris OX=9615 GN=SQSTM1 PE=4 SV=1 | A0A8I3N2A1 | *SQSTM1* | 50 kDa | -- | | #VALUE! | Missing Value |
| **1075** | Arginyl aminopeptidase OS=Canis lupus familiaris OX=9615 GN=RNPEP PE=3 SV=1 | A0A8I3NZM9 | *RNPEP* | 68 kDa | -- | | #VALUE! | Missing Value |
| **1076** | Tubulin beta chain OS=Canis lupus familiaris OX=9615 GN=TUBB6 PE=3 SV=1 | A0A8I3MYG1 | *TUBB6* | 50 kDa | -- | | #VALUE! | Missing Value |
| **1077** | Eukaryotic translation initiation factor 3 subunit I OS=Canis lupus familiaris OX=9615 GN=EIF3I PE=3 SV=1 | A0A8I3MMU7 | *EIF3I* | 36 kDa | -- | | #VALUE! | Missing Value |
| **1078** | TPD52 like 2 OS=Canis lupus familiaris OX=9615 GN=TPD52L2 PE=3 SV=1 | A0A8I3S471 | *TPD52L2* | 22 kDa | -- | | #VALUE! | Missing Value |
| **1079** | 2-iminobutanoate/2-iminopropanoate deaminase OS=Canis lupus familiaris OX=9615 GN=RIDA PE=3 SV=1 | A0A8I3PQP9 | *RIDA* | 14 kDa | -- | | #VALUE! | Missing Value |
| **1080** | UDP-glucose glycoprotein glucosyltransferase 1 OS=Canis lupus familiaris OX=9615 GN=UGGT1 PE=3 SV=1 | A0A8I3P9Z2 | *UGGT1* | 178 kDa | -- | | #VALUE! | Missing Value |
| **1081** | Myosin heavy chain 1 OS=Canis lupus familiaris OX=9615 GN=MYH1 PE=3 SV=1 | A0A8I3MN85 | *MYH1* | 225 kDa | -- | | #VALUE! | Missing Value |
| **1082** | Myosin heavy chain 8 OS=Canis lupus familiaris OX=9615 GN=MYH8 PE=3 SV=1 | A0A8I3RQG0 | *MYH8* | 222 kDa | -- | | #VALUE! | Missing Value |
| **1083** | Erythrocyte membrane protein band 4.1 like 2 OS=Canis lupus familiaris OX=9615 GN=EPB41L2 PE=4 SV=1 | A0A8I3MLL9 | *EPB41L2* | 120 kDa | -- | | #VALUE! | Missing Value |
| **1084** | S-(hydroxymethyl)glutathione dehydrogenase OS=Canis lupus familiaris OX=9615 GN=ADH5 PE=3 SV=1 | A0A8I3PVI9 | *ADH5* | 40 kDa | -- | | #VALUE! | Missing Value |
| **1085** | Carbonyl reductase (NADPH) OS=Canis lupus familiaris OX=9615 GN=CBR1 PE=3 SV=1 | A0A8I3S669 | *CBR1* | 33 kDa | -- | | #VALUE! | Missing Value |
| **1086** | Fibronectin type III domain containing 1 OS=Canis lupus familiaris OX=9615 GN=FNDC1 PE=4 SV=1 | A0A8I3MGH3 | *FNDC1* | 192 kDa | -- | | #VALUE! | Missing Value |
| **1087** | Latexin OS=Canis lupus familiaris OX=9615 GN=LXN PE=3 SV=1 | A0A8I3PT76 | *LXN* | 25 kDa | -- | | #VALUE! | Missing Value |
| **1088** | Tubulin folding cofactor B OS=Canis lupus familiaris OX=9615 GN=TBCB PE=3 SV=1 | A0A8I3MF18 | *TBCB* | 27 kDa | -- | | #VALUE! | Missing Value |
| **1089** | Myosin heavy chain 8 OS=Canis lupus familiaris OX=9615 GN=MYH8 PE=3 SV=1 | A0A8I3MLD2 | *MYH8* | 223 kDa | -- | | #VALUE! | Missing Value |
| **1090** | N-acetyl-alpha-glucosaminidase OS=Canis lupus familiaris OX=9615 GN=NAGLU PE=4 SV=1 | A0A8I3NA03 | *NAGLU* | 83 kDa | -- | | #VALUE! | Missing Value |
| **1091** | Eukaryotic translation initiation factor 4E OS=Canis lupus familiaris OX=9615 GN=LOC119866572 PE=3 SV=1 | A0A8I3QYF5 | *LOC119866572* | 21 kDa | -- | | #VALUE! | Missing Value |
| **1092** | 14 kDa phosphohistidine phosphatase OS=Canis lupus familiaris OX=9615 GN=PHPT1 PE=3 SV=1 | A0A8I3MSR7 | *PHPT1* | 14 kDa | -- | | #VALUE! | Missing Value |
| **1093** | Beta-galactosidase OS=Canis lupus familiaris OX=9615 GN=GLB1 PE=3 SV=1 | A0A8I3PJJ8 | *GLB1* | 75 kDa | -- | | #VALUE! | Missing Value |
| **1094** | Uncharacterized protein OS=Canis lupus familiaris OX=9615 PE=4 SV=1 | A0A8I3S2R9 | | 14 kDa | -- | | #VALUE! | Missing Value |
| **1095** | Apoptosis inducing factor mitochondria associated 1 OS=Canis lupus familiaris OX=9615 GN=AIFM1 PE=3 SV=1 | A0A8I3S7X8 | *AIFM1* | 63 kDa | -- | | #VALUE! | Missing Value |

| | | | | | | | | |
|---|---|---|---|---|---|---|---|---|
| **1096** | DnaJ heat shock protein family (Hsp40) member C8 OS=Canis lupus familiaris OX=9615 GN=DNAJC8 PE=4 SV=1 | A0A8I3NB10 | *DNAJC8* | 30 kDa | -- | | #VALUE! | Missing Value |
| **1097** | Hydroxyacylglutathione hydrolase OS=Canis lupus familiaris OX=9615 GN=HAGH PE=3 SV=1 | A0A8I3P3Q9 | *HAGH* | 47 kDa | -- | | #VALUE! | Missing Value |
| **1098** | ATP synthase subunit beta OS=Canis lupus familiaris OX=9615 GN=ATP5F1B PE=3 SV=1 | A0A8I3NCH2 | *ATP5F1B* | 54 kDa | -- | | #VALUE! | Missing Value |
| **1099** | Myosin-2 OS=Canis lupus familiaris OX=9615 GN=MYH2 PE=3 SV=1 | Q076A7 | *MYH2* | 223 kDa | -- | | #VALUE! | Missing Value |
| **1100** | CLN5 intracellular trafficking protein OS=Canis lupus familiaris OX=9615 GN=CLN5 PE=3 SV=1 | A0A8I3PXR4 | *CLN5* | 22 kDa | -- | | #VALUE! | Missing Value |
| **1101** | Adenylosuccinate lyase OS=Canis lupus familiaris OX=9615 GN=ADSL PE=3 SV=1 | A0A8I3P3A3 | *ADSL* | 55 kDa | -- | | #VALUE! | Missing Value |
| **1102** | TNF alpha induced protein 6 OS=Canis lupus familiaris OX=9615 GN=TNFAIP6 PE=4 SV=1 | A0A8I3N7F9 | *TNFAIP6* | 31 kDa | -- | | #VALUE! | Missing Value |
| **1103** | Protein kinase cAMP-dependent type II regulatory subunit alpha OS=Canis lupus familiaris OX=9615 GN=PRKAR2A PE=3 SV=1 | A0A8I3QLN7 | *PRKAR2A* | 45 kDa | -- | | #VALUE! | Missing Value |
| **1104** | Serine hydroxymethyltransferase OS=Canis lupus familiaris OX=9615 GN=SHMT2 PE=3 SV=1 | A0A8I3N480 | *SHMT2* | 56 kDa | -- | | #VALUE! | Missing Value |
| **1105** | ADAM metallopeptidase with thrombospondin type 1 motif 1 OS=Canis lupus familiaris OX=9615 GN=ADAMTS1 PE=4 SV=1 | A0A8I3P1X0 | *ADAMTS1* | 102 kDa | -- | | #VALUE! | Missing Value |
| **1106** | Polypeptide N-acetylgalactosaminyltransferase OS=Canis lupus familiaris OX=9615 GN=GALNT2 PE=3 SV=1 | A0A8I3MY69 | *GALNT2* | 65 kDa | -- | | #VALUE! | Missing Value |
| **1107** | Ribonuclease T2 OS=Canis lupus familiaris OX=9615 GN=RNASET2 PE=3 SV=1 | A0A8I3MXP5 | *RNASET2* | 27 kDa | -- | | #VALUE! | Missing Value |
| **1108** | Dihydrolipoyllysine-residue succinyltransferase component of 2-oxoglutarate dehydrogenase complex, mitochondrial OS=Canis lupus familiaris OX=9615 GN=DLST PE=3 SV=1 | A0A8I3NVG0 | *DLST* | 65 kDa | -- | | #VALUE! | Missing Value |
| **1109** | Endoplasmic reticulum protein 44 OS=Canis lupus familiaris OX=9615 GN=ERP44 PE=4 SV=1 | A0A8I3N2H6 | *ERP44* | 54 kDa | -- | | #VALUE! | Missing Value |
| **1110** | ENAH actin regulator OS=Canis lupus familiaris OX=9615 GN=ENAH PE=3 SV=1 | A0A8I3NAJ3 | *ENAH* | 69 kDa | -- | | #VALUE! | Missing Value |
| **1111** | Platelet-derived growth factor receptor-like protein OS=Canis lupus familiaris OX=9615 GN=PDGFRL PE=4 SV=1 | A0A8I3NZC5 | *PDGFRL* | 42 kDa | -- | | #VALUE! | Missing Value |
| **1112** | Uncharacterized protein OS=Canis lupus familiaris OX=9615 GN=BSG PE=4 SV=1 | A0A8I3P4K7 | *BSG* | 35 kDa | -- | | #VALUE! | Missing Value |
| **1113** | Cystathionine gamma-lyase OS=Canis lupus familiaris OX=9615 GN=CTH PE=3 SV=1 | A0A8I3NI74 | *CTH* | 59 kDa | -- | | #VALUE! | Missing Value |
| **1114** | Tripeptidyl-peptidase 2 OS=Canis lupus familiaris OX=9615 GN=TPP2 PE=3 SV=1 | A0A8I3PZT5 | *TPP2* | 130 kDa | -- | | #VALUE! | Missing Value |
| **1115** | Caspase 14 OS=Canis lupus familiaris OX=9615 GN=CASP14 PE=3 SV=1 | A0A8I3NXI7 | *CASP14* | 28 kDa | -- | | #VALUE! | Missing Value |
| **1116** | Cathepsin H OS=Canis lupus familiaris OX=9615 GN=CTSH PE=3 SV=1 | A0A8I3MSD7 | *CTSH* | 37 kDa | -- | | #VALUE! | Missing Value |
| **1117** | Periplakin OS=Canis lupus familiaris OX=9615 GN=PPL PE=4 SV=1 | A0A8I3NRN0 | *PPL* | 191 kDa | -- | | #VALUE! | Missing Value |
| **1118** | Serum amyloid A protein OS=Canis lupus familiaris OX=9615 GN=SAA1 PE=3 SV=1 | A0A8I3NYD5 | *SAA1* | 14 kDa | -- | | #VALUE! | Missing Value |

| | | | | | | | | |
|---|---|---|---|---|---|---|---|---|
| **1119** | REVERSE_A0A8I3RW02 | REVERSE_A0A8I3RW02 | | | -- | | #VALUE! | Missing Value |
| **1120** | Mitogen-activated protein kinase OS=Canis lupus familiaris OX=9615 GN=MAPK3 PE=3 SV=1 | A0A8I3N0N1 | *MAPK3* | 39 kDa | -- | | #VALUE! | Missing Value |
| **1121** | RNA helicase OS=Canis lupus familiaris OX=9615 GN=DDX3X PE=3 SV=1 | A0A8I3PXH6 | *DDX3X* | 73 kDa | -- | | #VALUE! | Missing Value |
| **1122** | ATP synthase F1 subunit delta OS=Canis lupus familiaris OX=9615 GN=ATP5F1D PE=3 SV=1 | A0A8I3NCZ3 | *ATP5F1D* | 18 kDa | -- | | #VALUE! | Missing Value |
| **1123** | Alpha-mannosidase OS=Canis lupus familiaris OX=9615 GN=MAN2B1 PE=3 SV=1 | A0A8I3NS02 | *MAN2B1* | 110 kDa | -- | | #VALUE! | Missing Value |
| **1124** | Amyloid beta precursor like protein 2 OS=Canis lupus familiaris OX=9615 GN=APLP2 PE=3 SV=1 | A0A8I3N111 | *APLP2* | 85 kDa | -- | | #VALUE! | Missing Value |
| **1125** | Collectin subfamily member 12 OS=Canis lupus familiaris OX=9615 GN=COLEC12 PE=4 SV=1 | A0A8I3MJN7 | *COLEC12* | 82 kDa | -- | | #VALUE! | Missing Value |
| **1126** | EMAP like 1 OS=Canis lupus familiaris OX=9615 GN=EML1 PE=3 SV=1 | A0A8I3MVL2 | *EML1* | 92 kDa | -- | | #VALUE! | Missing Value |
| **1127** | Hyaluronidase OS=Canis lupus familiaris OX=9615 GN=HYAL1 PE=3 SV=1 | A0A8I3PI48 | *HYAL1* | 88 kDa | -- | | #VALUE! | Missing Value |
| **1128** | Legumain OS=Canis lupus familiaris OX=9615 GN=LGMN PE=3 SV=1 | A0A8I3PCQ2 | *LGMN* | 46 kDa | -- | | #VALUE! | Missing Value |
| **1129** | Matrix metallopeptidase 12 OS=Canis lupus familiaris OX=9615 GN=MMP12 PE=3 SV=1 | A0A8I3MBX9 | *MMP12* | 53 kDa | -- | | #VALUE! | Missing Value |
| **1130** | Matrix metalloproteinase-9 OS=Canis lupus familiaris OX=9615 GN=MMP9 PE=3 SV=1 | A0A8I3S2Q7 | *MMP9* | 77 kDa | -- | | #VALUE! | Missing Value |
| **1131** | Peptidylglycine alpha-amidating monooxygenase OS=Canis lupus familiaris OX=9615 GN=PAM PE=3 SV=1 | A0A8I3MEC5 | *PAM* | 107 kDa | -- | | #VALUE! | Missing Value |
| **1132** | Plexin domain containing 2 OS=Canis lupus familiaris OX=9615 GN=PLXDC2 PE=4 SV=1 | A0A8I3NAY5 | *PLXDC2* | 50 kDa | -- | | #VALUE! | Missing Value |
| **1133** | Synaptotagmin 9 OS=Canis lupus familiaris OX=9615 GN=OLFML1 PE=4 SV=1 | A0A8I3N4Q3 | *OLFML1* | 97 kDa | -- | | #VALUE! | Missing Value |
| **1134** | Uncharacterized protein OS=Canis lupus familiaris OX=9615 GN=SCARF2 PE=4 SV=1 | A0A8I3QXK3 | *SCARF2* | 91 kDa | -- | | #VALUE! | Missing Value |
| **1135** | Globin family profile domain-containing protein OS=Canis lupus familiaris OX=9615 GN=HBQ1 PE=3 SV=1 | A0A8I3MCD6 | *HBQ1* | 22 kDa | -- | | #VALUE! | Reference Missing |
| **1136** | Lactotransferrin OS=Canis lupus familiaris OX=9615 GN=LTF PE=3 SV=1 | A0A8I3NKN6 | *LTF* | 66 kDa | -- | | #VALUE! | Reference Missing |
| **1137** | AP-2 complex subunit alpha OS=Canis lupus familiaris OX=9615 GN=AP2A2 PE=3 SV=1 | A0A8I3N7P9 | *AP2A2* | 106 kDa | -- | | #VALUE! | Reference Missing |
| **1138** | Complement C5 OS=Canis lupus familiaris OX=9615 GN=C5 PE=4 SV=1 | A0A8I3NVC1 | *C5* | 188 kDa | -- | | #VALUE! | Reference Missing |
| **1139** | Carbonic anhydrase OS=Canis lupus familiaris OX=9615 GN=CA2 PE=3 SV=1 | A0A8I3P3N6 | *CA2* | 29 kDa | -- | | #VALUE! | Reference Missing |
| **1140** | Transglutaminase 2 OS=Canis lupus familiaris OX=9615 GN=TGM2 PE=3 SV=1 | A0A8I3Q5I7 | *TGM2* | 70 kDa | -- | | #VALUE! | Reference Missing |
| **1141** | Serpin family D member 1 OS=Canis lupus familiaris OX=9615 GN=SERPIND1 PE=3 SV=1 | A0A8I3PXZ6 | *SERPIND1* | 62 kDa | -- | | #VALUE! | Reference Missing |
| **1142** | Phosphoinositide phospholipase C OS=Canis lupus familiaris OX=9615 GN=PLCL2 PE=4 SV=1 | A0A8I3PIB6 | *PLCL2* | 126 kDa | -- | | #VALUE! | Reference Missing |

| | | | | | | | | |
|---|---|---|---|---|---|---|---|---|
| **1143** | Serine peptidase inhibitor Kazal type 5 OS=Canis lupus familiaris OX=9615 GN=spink5 PE=2 SV=1 | Q4VYB4 | *spink5* | 121 kDa | -- | | #VALUE! | Reference Missing |
| **1144** | Matrix metallopeptidase 1 OS=Canis lupus familiaris OX=9615 GN=MMP1 PE=3 SV=1 | A0A8I3MGK8 | *MMP1* | 51 kDa | -- | | #VALUE! | Reference Missing |
| **1145** | CD93 molecule OS=Canis lupus familiaris OX=9615 GN=CD93 PE=4 SV=1 | A0A8I3Q0M4 | *CD93* | 68 kDa | -- | | #VALUE! | Reference Missing |
| **1146** | Leucine zipper transcription factor-like protein 1 OS=Canis lupus familiaris OX=9615 GN=LZTFL1 PE=3 SV=1 | A0A8I3RX08 | *LZTFL1* | 35 kDa | -- | | #VALUE! | Reference Missing |
| **1147** | isoleucine--tRNA ligase OS=Canis lupus familiaris OX=9615 GN=IARS1 PE=3 SV=1 | A0A8I3RNX5 | *IARS1* | 131 kDa | -- | | #VALUE! | Reference Missing |
| **1148** | Obg-like ATPase 1 OS=Canis lupus familiaris OX=9615 GN=OLA1 PE=3 SV=1 | A0A8I3SCD6 | *OLA1* | 46 kDa | -- | | #VALUE! | Reference Missing |
| **1149** | Hydroxymethylglutaryl-CoA synthase OS=Canis lupus familiaris OX=9615 GN=HMGCS1 PE=3 SV=1 | A0A8I3MM03 | *HMGCS1* | 57 kDa | -- | | #VALUE! | Reference Missing |
| **1150** | Lysyl oxidase homolog OS=Canis lupus familiaris OX=9615 GN=LOXL3 PE=3 SV=1 | A0A8I3PD96 | *LOXL3* | 67 kDa | -- | | #VALUE! | Reference Missing |
| **1151** | Proteasome 26S subunit, non-ATPase 14 OS=Canis lupus familiaris OX=9615 GN=PSMD14 PE=4 SV=1 | A0A8I3QV78 | *PSMD14* | 35 kDa | -- | | #VALUE! | Reference Missing |
| **1152** | Cadherin 5 OS=Canis lupus familiaris OX=9615 GN=CDH5 PE=4 SV=1 | A0A8I3NHK1 | *CDH5* | 86 kDa | -- | | #VALUE! | Reference Missing |
| **1153** | IF rod domain-containing protein OS=Canis lupus familiaris OX=9615 GN=KRT7 PE=3 SV=1 | A0A8I3PYC8 | *KRT7* | 53 kDa | -- | | #VALUE! | Reference Missing |
| **1154** | SPARC related modular calcium binding 1 OS=Canis lupus familiaris OX=9615 GN=SMOC1 PE=4 SV=1 | A0A8I3MU53 | *SMOC1* | 48 kDa | -- | | #VALUE! | Reference Missing |
| **1155** | EH domain containing 4 OS=Canis lupus familiaris OX=9615 GN=EHD4 PE=4 SV=1 | A0A8I3Q6L5 | *EHD4* | 61 kDa | -- | | #VALUE! | Reference Missing |
| **1156** | Macrophage stimulating 1 OS=Canis lupus familiaris OX=9615 GN=MST1 PE=3 SV=1 | A0A8I3PQK9 | *MST1* | 82 kDa | -- | | #VALUE! | Reference Missing |
| **1157** | Adenylosuccinate synthetase OS=Canis lupus familiaris OX=9615 GN=ADSS2 PE=3 SV=1 | A0A8I3MYZ6 | *ADSS2* | 46 kDa | -- | | #VALUE! | Reference Missing |
| **1158** | Annexin OS=Canis lupus familiaris OX=9615 GN=ANXA8L1 PE=3 SV=1 | A0A8I3N3F1 | *ANXA8L1* | 30 kDa | -- | | #VALUE! | Reference Missing |
| **1159** | S-formylglutathione hydrolase OS=Canis lupus familiaris OX=9615 GN=ESD PE=3 SV=1 | A0A8I3MU76 | *ESD* | 31 kDa | -- | | #VALUE! | Reference Missing |
| **1160** | Neuronal pentraxin 1 OS=Canis lupus familiaris OX=9615 GN=NPTX1 PE=4 SV=1 | A0A8I3NC72 | *NPTX1* | 47 kDa | -- | | #VALUE! | Reference Missing |
| **1161** | Anamorsin OS=Canis lupus familiaris OX=9615 GN=CIAPIN1 PE=3 SV=1 | A0A8I3MPT1 | *CIAPIN1* | 34 kDa | -- | | #VALUE! | Reference Missing |
| **1162** | Transcobalamin 2 OS=Canis lupus familiaris OX=9615 GN=TCN2 PE=3 SV=1 | A0A8I3PAV5 | *TCN2* | 48 kDa | -- | | #VALUE! | Reference Missing |
| **1163** | IF rod domain-containing protein OS=Canis lupus familiaris OX=9615 GN=KRT7 PE=3 SV=1 | A0A8I3S5H0 | *KRT7* | 52 kDa | -- | | #VALUE! | Reference Missing |
| **1164** | Stabilin 1 OS=Canis lupus familiaris OX=9615 GN=STAB1 PE=4 SV=1 | A0A8I3Q3R5 | *STAB1* | 276 kDa | -- | | #VALUE! | Reference Missing |
| **1165** | Tissue factor pathway inhibitor OS=Canis lupus familiaris OX=9615 GN=TFPI PE=2 SV=1 | Q28874 | *TFPI* | 44 kDa | -- | | #VALUE! | Reference Missing |
| **1166** | Integrin beta OS=Canis lupus familiaris OX=9615 GN=ITGB1 PE=3 SV=1 | A0A8I3MGZ7 | *ITGB1* | 82 kDa | -- | | #VALUE! | Reference Missing |

| | | | | | | | | |
|---|---|---|---|---|---|---|---|---|
| **1167** | Niban apoptosis regulator 2 OS=Canis lupus familiaris OX=9615 GN=NIBAN2 PE=3 SV=1 | A0A8I3NCX0 | *NIBAN2* | 84 kDa | -- | | #VALUE! | Reference Missing |
| **1168** | IST1 homolog OS=Canis lupus familiaris OX=9615 GN=IST1 PE=3 SV=1 | A0A8I3N3H0 | *IST1* | 41 kDa | -- | | #VALUE! | Reference Missing |
| **1169** | Uncharacterized protein OS=Canis lupus familiaris OX=9615 PE=3 SV=1 | A0A8I3MH53 | | 9 kDa | -- | | #VALUE! | Reference Missing |
| **1170** | RNA-splicing ligase RtcB homolog OS=Canis lupus familiaris OX=9615 GN=RTCB PE=3 SV=1 | A0A8I3N8Q9 | *RTCB* | 54 kDa | -- | | #VALUE! | Reference Missing |
| **1171** | U5 small nuclear ribonucleoprotein 200 kDa helicase OS=Canis lupus familiaris OX=9615 GN=SNRNP200 PE=3 SV=1 | A0A8I3MZW5 | *SNRNP200* | 239 kDa | -- | | #VALUE! | Reference Missing |
| **1172** | MAM domain containing 2 OS=Canis lupus familiaris OX=9615 GN=MAMDC2 PE=4 SV=1 | A0A8I3MFC9 | *MAMDC2* | 78 kDa | -- | | #VALUE! | Reference Missing |
| **1173** | Plasmalemma vesicle associated protein OS=Canis lupus familiaris OX=9615 GN=PLVAP PE=4 SV=1 | A0A8I3PUQ9 | *PLVAP* | 50 kDa | -- | | #VALUE! | Reference Missing |
| **1174** | Platelet and endothelial cell adhesion molecule 1 OS=Canis lupus familiaris OX=9615 GN=PECAM1 PE=4 SV=1 | A0A8I3PLT7 | *PECAM1* | 81 kDa | -- | | #VALUE! | Reference Missing |
| **1175** | Protein C receptor OS=Canis lupus familiaris OX=9615 GN=PROCR PE=4 SV=1 | A0A8I3P760 | *PROCR* | 30 kDa | -- | | #VALUE! | Reference Missing |
| **1176** | Protein phosphatase, Mg2+/Mn2+ dependent 1F OS=Canis lupus familiaris OX=9615 GN=PPM1F PE=3 SV=1 | A0A8I3QAP2 | *PPM1F* | 51 kDa | -- | | #VALUE! | Reference Missing |
| **1177** | Ribonucloprotein OS=Canis lupus familiaris OX=9615 GN=SNU13 PE=3 SV=1 | A0A8I3P775 | *SNU13* | 15 kDa | -- | | #VALUE! | Reference Missing |
| **1178** | Complement component C6 OS=Canis lupus familiaris OX=9615 GN=C6 PE=3 SV=1 | A0A8I3MIG2 | *C6* | 106 kDa | -- | | #VALUE! | Reference Missing |
| **1179** | Septin OS=Canis lupus familiaris OX=9615 GN=SEPTIN10 PE=3 SV=1 | A0A8I3RTH6 | *SEPTIN10* | 55 kDa | -- | | #VALUE! | Reference Missing |
| **1180** | Glutathione transferase OS=Canis lupus familiaris OX=9615 PE=4 SV=1 | A0A8I3S4W3 | | 26 kDa | -- | | #VALUE! | Reference Missing |
| **1181** | Methionine--tRNA ligase, cytoplasmic OS=Canis lupus familiaris OX=9615 GN=MARS1 PE=3 SV=1 | A0A8I3NFF8 | *MARS1* | 101 kDa | -- | | #VALUE! | Reference Missing |
| **1182** | Fumarylacetoacetase OS=Canis lupus familiaris OX=9615 GN=FAH PE=3 SV=1 | A0A8I3MPR1 | *FAH* | 59 kDa | -- | | #VALUE! | Reference Missing |
| **1183** | Serine/threonine-protein phosphatase OS=Canis lupus familiaris OX=9615 GN=PPP3CB PE=3 SV=1 | A0A8I3MW99 | *PPP3CB* | 60 kDa | -- | | #VALUE! | Reference Missing |
| **1184** | Arsenite-resistance protein 2 OS=Canis lupus familiaris OX=9615 GN=SRRT PE=3 SV=1 | A0A8I3MWW9 | *SRRT* | 101 kDa | -- | | #VALUE! | Reference Missing |
| **1185** | MICOS complex subunit MIC60 OS=Canis lupus familiaris OX=9615 GN=IMMT PE=3 SV=1 | A0A8I3NPU5 | *IMMT* | 87 kDa | -- | | #VALUE! | Reference Missing |
| **1186** | Rho GDP dissociation inhibitor beta OS=Canis lupus familiaris OX=9615 GN=ARHGDIB PE=3 SV=1 | A0A8I3P6H9 | *ARHGDIB* | 23 kDa | -- | | #VALUE! | Reference Missing |
| **1187** | Uncharacterized protein OS=Canis lupus familiaris OX=9615 GN=KRT38 PE=3 SV=1 | A0A8I3NYA0 | *KRT38* | 51 kDa | -- | | #VALUE! | Reference Missing |
| | **END OF FILE** | | | | | | | |
