# Supplementary figures and images for: In vitro aging alters the gene expression and secretome composition of canine adipose-derived mesenchymal stem cells
Source: Front Vet Sci. 2024 Mar 28;11:1387174. doi: 10.3389/fvets.2024.1387174 (PMC11006985; doi:10.3389/fvets.2024.1387174)

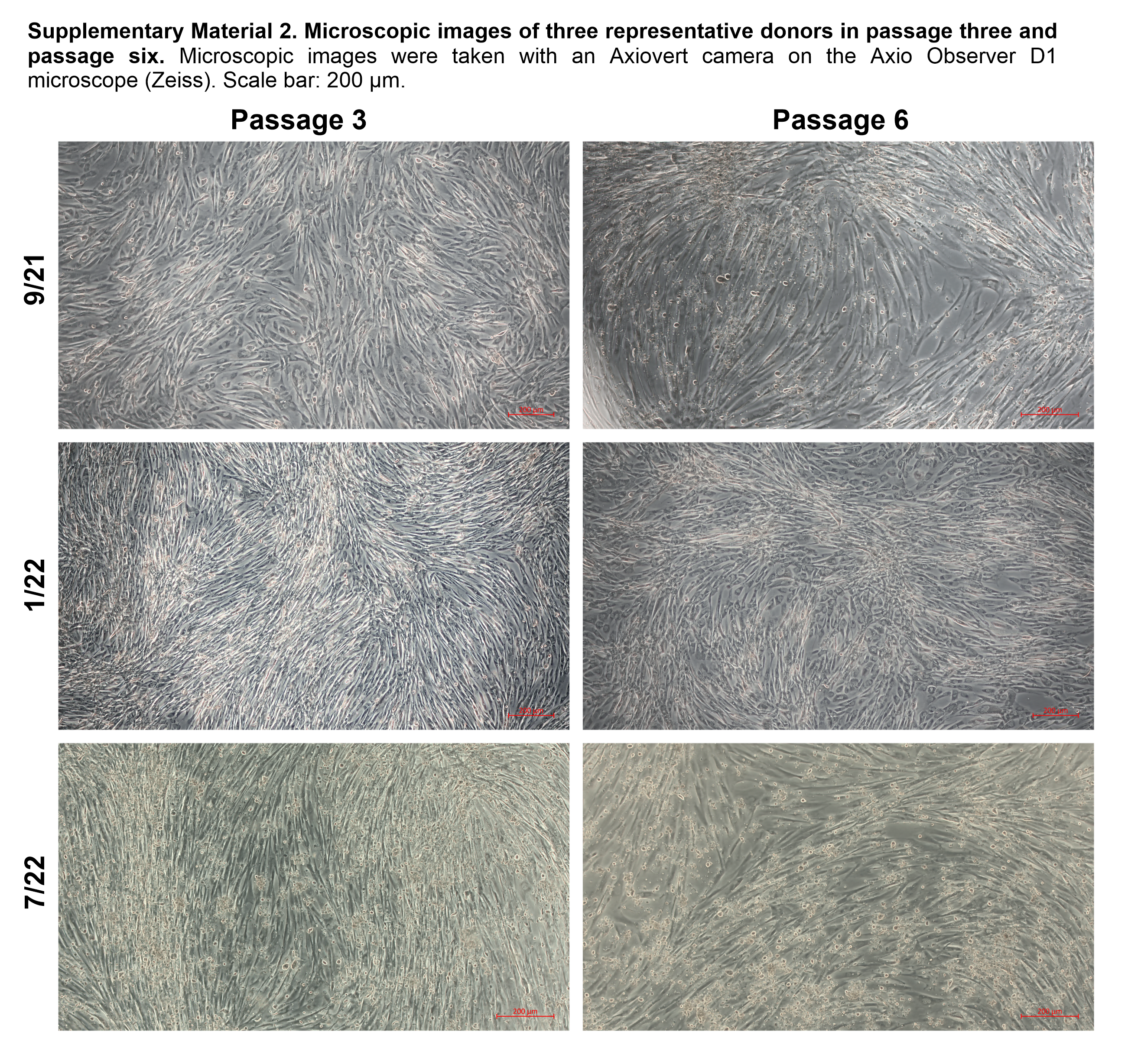

Supplement: Supplementary file 3 [file Image_1.TIF]
